# Synthesis and Characterization of Single Crystal Zircon-Hafnon Zr_(1–*x*)_Hf_(*x*)_SiO_4_ Solid Solutions and the Comparison with the Reaction Products of a TEOS-Based Hydrothermal Route

**DOI:** 10.1021/acsomega.3c06960

**Published:** 2024-03-25

**Authors:** Andreas Neumann, Volker Kahlenberg, Ian Lerche, Stefan Stöber, Herbert Pöllmann

**Affiliations:** † Institute of Geoscience and Geography, Martin-Luther-University Halle-Wittenberg, Von-Seckendorff-Platz 3, D-06120 Halle (Saale), Germany; ‡ Institute of Mineralogy and Petrography, University of Innsbruck, Innrain 52, A-6020 Innsbruck, Austria

## Abstract

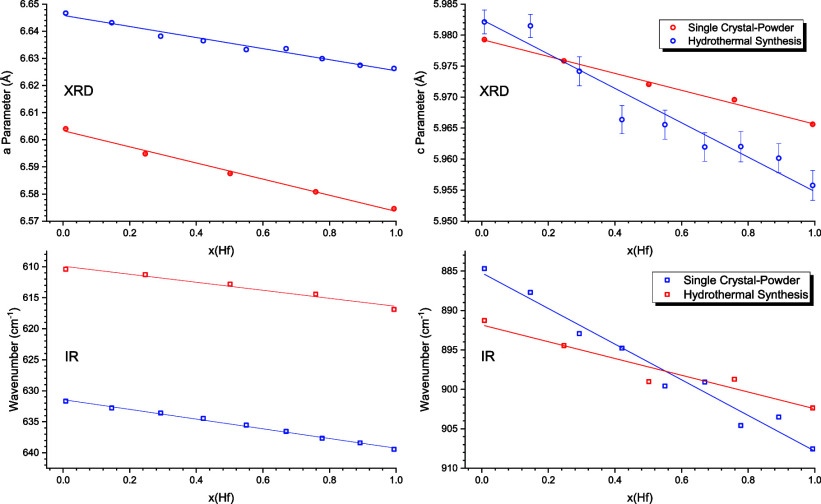

Two synthesis routes of the zircon–hafnon solid solution series were carried out. The high-temperature route uses the growth of single crystals via a flux mixture that has been cooled down slowly from 1400 °C over 4 weeks. The reaction products were colorless and idiomorphic without byproducts. The hydrothermal tetraethoxysilane (TEOS)-based route represents the low-temperature method at 200 °C for approximately 7 days. The hydrothermal route yielded a white powder and scanning electron microscopy analysis thereof did not reveal any specific idiomorphic properties. However, the synthesis also featured some byproducts besides the zircon–hafnon solid solutions. Thermogravimetric analysis coupled with differential scanning calorimetry, and mass spectroscopy indicated, that hydrothermal reaction products feature the presence of organic residues originating from the starting materials. However, a specific dependency on the hafnium content could not be observed due to the data scatter. Infrared (IR) analysis revealed the presence of Zr/Hf-oxides. The structural characterization demonstrated that properties change constantly with the hafnium amount, however, gradual variations of some properties related to the composition of the solid solution series depend in part on the synthesis route, considering the *c*/*a* ratio and IR modes. Furthermore, analyses of the single crystals by Raman spectroscopy and μXRF suggested a nonequilibrated crystal growth based on the starting composition.

## 1 Introduction

Zircon occurs naturally as a mineral. Due to its resistance toward degradation and its ability to host uranium, this material is very important in geological dating [cf. e.g., refs (1,2)]. This stability, which preserves the mineral for billions of years is of interest for technical applications as well. Low thermal expansion and thermal stability (cf. ref (3)) are often a desired property in the ceramic industry. Furthermore, due to its resistance against irradiation synthetic zircon may offer a potential to host radionuclides, which originate from spent nuclear fuel (abbrev. SNF) or from the dismantling of nuclear weapons (cf. refs (4−6)) and hence being an alternative for direct disposal. Such concepts are based on the specific feature of zircon to be isostructural with thorite (ThSiO_4_) and coffinite (USiO_4_). These minerals belong to the space group *I4*_*1*_*amd* (No. 141), hence the incorporation of uranium, thorium, and other specific actinides to form solid solutions is likely. In addition, there are also implications for the safety assessment of nuclear issues (e.g., corium) when research focuses on radiologically very challenging environments, which feature very high radiation doses (cf. refs (7,8)). Respective spectroscopic research on zircon with radiation damage was carried out e.g. by Nasdala et al.,^9^ Titorenkova et al.,^10^ Woodhead et al.,^11^ and Zhang.^12−16^ The outstanding properties of zircon for waste management applications could be extended when hafnium is added as a component of the system to form ZHSS (zircon-hafnon solid solutions). Due to the nearly identical ionic radii (*r*(Zr^4+^) = 0.84 Å, *r*(Hf^4+^) = 0.83 Å^17^ hafnium could easily replace zirconium and hence forms a full solid solution series with zircon. Therefore, according to a previous study,^18^ the hafnium-rich end member is called hafnon, also observed naturally, however, the abundance is rather scarce. Nevertheless, due to this complete miscibility, desired properties can be designed and engineered very specifically, considering the thermal expansion–which is even lower for hafnon–or specific electric properties for high-κ (high dielectric constant kappa) applications.^19^ The special benefit to SNF is that hafnium is a neutron absorbing agent (cf. refs (5,20)) and hence accounts for criticality issues e.g. in a final repository, which has to be concerned about nuclear safety regulations. However, research with respect to the structure is mainly focused on zircon likely due to the higher abundance of zircon. The ICSD database 2018.2^21^ currently lists 33 crystallographic data sets for zircon.^22−37^ However, only two entries exist for hafnon:.^38,39^ Hence, the same pattern seems to be valid for synthesis methods at low temperatures since hafnium behaves chemically in a similar manner to zirconium. Most of these protocols describe the use of Zr-oxychloride, ZIP (Zr(OC_3_H_7_)_4_), and tetraethoxy-silane (TEOS) as starting materials (cf. refs (40−49,34,50)), to which NaF as a mineralizer has been added in some cases. The reported reaction products were calcined and regularly showed the formation of distinct zircon reflections in powder X-ray diffraction (PXRD). In addition to zircon, the temperature-dependent formation of both tetragonal and monoclinic ZrO_2_ (Baddeleyite) and Cristobalite was also observed. However, with a further increase in temperature, these compounds were consumed by solid reactions to form zircon. With respect to a low-temperature synthesis of hafnon, Kanno^51^ described a sol–gel route of reaction products, which were afterward calcined, and the XRD analysis showed the reflections of HfSiO_4_, HfO_2_, and SiO_2_. Research by Estevenon et al.^52^ used Na_2_SiO_3_·5H_2_O and HfCl_4_ as aqueous silicate and hafnium precursors for a low-temperature hydrothermal synthesis of HfSiO_4_. Temperatures were 250 °C or less and different pH were applied to produce successfully pure hafnon. The XRD analysis showed that samples with very anisotropic peak broadening featured the largest values for the *a*- (6.6447(3) Å) and *c*-parameters (5.9801(9) Å). Crystallite sizes (CS) were very small (16(14) nm). Thermal analysis showed a weight loss between ∼6 and 12.5 wt % depending on the synthesis temperature.

The vibrational spectrum characterization of zircon was carried out by Dawson et al.^53^ Further studies comparing zircon and hafnon or on solid solutions thereof are not very widespread. The study of Ramakrishnan et al.^54^ prepared and investigated the lattice parameters of the ZHSS and observed complete solubility. Nicola and Rutt^55^ analyzed the Raman spectra of end-members of this solid solution series and found that rotational and translational modes of the same symmetry can be identified by comparing the spectra. Syme et al.^56^ investigated the spectroscopic properties of zircon and thorite. A comparison between the zircon and thorite results enables an assignment to be made of the Raman spectrum of Hafnon. Hoskin and Rodgers^57^ investigated the full solid solution with Raman spectroscopy and observed a distinct linearity of shifts in the ZHSS. Experimental and computational work by Grüneberger et al.^58^ focused on the Raman spectra of the synthesized full zircon–hafnon solid solution series and lattice parameters of the end-members were reported as well. They observed the zoning and coexistence of Zr–Zr and Hf–Hf external modes of the intermediates. Cota et al.^59^ published a combined experimental and theoretical study of the solid solution series. The experimental part comprised also the synthesis of crystals of the solid solution series, which they analyzed by PXRD, NMR, and first principle calculations. The data exhibited a pronounced negative deviation from Vegard’s law.

This study presents a direct comparison of the zircon–hafnon solid solutions, which were obtained by two different experimental approaches. The authors used a commonly applied flux method (cf. ref (60)) for the synthesis of single crystals and described a simple method for the hydrothermal synthesis. The reaction products from both approaches were mainly characterized by optical and electron microscopy, XRD, Raman, and IR spectroscopy. μXRF was also applied and complemented the Raman spectroscopy of the single crystals. Further characterization of the hydrothermally produced solid solution series was carried out by a combined simultaneous TG, DSC, IR, and MS analysis. Associated Content and data labeled as Figure/Table SXX are in the Supporting Information document.

## 2 Experimental Section

### 2.1 Materials and Synthesis

The major compounds ZrOCl_2_·8H_2_O (99.9%), HfOCl_2_·8H_2_O (98%), and tetraethoxysilane (TEOS, 99.9) were supplied by Alfa Aesar. Molybdenum-VI-oxide 99.9% and lithium carbonate 99.9% were supplied by Merck. For the SiO_2_ component, fluffy Aerosil powder was used. Additional chemicals were used from common suppliers.

A nonstoichiometric ratio (Zr_1–*x*_Hf_*x*_/Si < 1) of the Zr_1–*x*_Hf_*x*_ (with *x* = 0–1) and Si starting compounds were used, i.e., TEOS was supplied in excess in order to account for the hygroscopic behavior of ZrOCl_2_·(8 + *y*)H_2_O (with *y* ∼ 5). The determination of the loss on ignition (LOI) for HfOCl_2_·8H_2_O revealed no excess water. These two compounds and TEOS were mixed with ethanol (approximately 7.8 mL) for the hydrothermal synthesis. Before the autoclave vessel with a Teflon liner (35 mL) was closed approximately 0.27 mL of 65% nitric acid and approximately 1 mL of 10% KOH base were added as well. The base was prepared by dissolution of KOH pellets (p.a.) supplied by Merck. The tightly closed steel autoclaves were put in a programmable Memmert drying oven at 200 °C. After approximately 7 days of heat treatment a cooling ramp was initiated and cooled the autoclaves down to 50 °C within an additional 7 days. The white reaction products were retrieved, washed, dried (at ∼100 °C), and prepared for characterization.

For the synthesis of the single crystals of the zircon–hafnon solid solution series (ZHSS), the preparation followed basically the protocol described by Cherniak et al.^60^ A Li_2_O·3MoO_3_ flux was prepared and mixed with defined ratios of ZrO_2_, HfO_2_, and SiO_2_. Zirconium and hafnium oxide were prepared by thermal decomposition of ZrOCl_2_·8H_2_O and HfOCl_2_·8H_2_O at 1000 °C. To obtain an accurate 1:1 Zr_1–*x*_Hf_*x*_/Si ratio the LOI at 1000 °C of the zirconium/hafnium and silicon precursors were determined. To obtain 0.5 g of Zr_1–*x*_Hf_*x*_SiO_4_ about 15 g Li_2_O·3MoO_3_ were added. The zircon flux mixture was thoroughly homogenized in an agate mortar and transferred to a platinum crucible that was additionally covered for heat treatment. The crucible with the mixture was put into a chamber furnace from Therm-Aix at 1000 °C. Within a few minutes, the system was fired up to 1400 °C to initiate a cooling ramp of 1 K/h for crystal growth. At approximately 900 °C the crystals were extracted from the flux residues, washed with deionized water, and prepared for further analysis.

### 2.2 XRD and XRF Characterization

The single crystal measurements for evaluation of the structure were carried out on a four-circle diffractometer ‘Xcalibur, Ruby, Gemini ultra with an CCD plate detector using a MoKα_1,2_ (λ = 0.71073 Å) tube. Data collection, cell refinement, and data reduction were carried out with CrysAlisPro Version 1.171.40.84a. Further data treatment, i.e. structure solution and refinement were carried out with SHELXS and SHELXL,^61^ using the WinGX suite.^62^ The compositions of the flux-grown single crystals were checked with a micro X-ray fluorescence system (μXRF) M4 Tornado from Bruker featuring a 20 μm X-ray spot for analysis. Samples (cf. Figure S13 in Supporting Information Document) were measured with an Ag tube and two simultaneously recording X-Flash detectors in vacuum mode (i.e., 20 mbar) at 50 kV and 300 μA.

PXRD diffractograms were recorded with two diffractometers from Panalytical:

1. X’Pert Pro Powder diffractometer using a 0.125° divergence slit with a CuKα_1,2_ (λ = 1.54059 Å) tube, being operated at 40 kV and 40 mA. Further device characteristics were a Ni filter, 0.08 rad primary soller slits, a position sensitive detector XCelerator covering 2.118° 2θ. Diffractograms were recorded from 10 to 125° 2θ with a step size of 0.033° and 100.33 s per step.
2. X’Pert Powder diffractometer with 0.25° divergence slit with CuKα_1,2_ (λ = 1.54059 Å) radiation, operated at 45 kV and 40 mA featuring a Ni filter, 0.04 rad primary soller slits with position sensitive detector Pixcel 1D covering 3.347° 2θ. Diffractograms were recorded from 10 to 125° 2θ with a step size of 0.026° and 96.36 s per step.

Powder diffractograms were evaluated with the DiffracPlus EVA-Software by Bruker-AXS and with the Highscore X’Pert Suite by Panalytical. PDF-2 and PDF-4 databases from ICDD were used for the analysis. For anisotropic refinements and quantitative analysis Topas Academic TA6 (cf. refs (63,64)) was employed for the Rietveld method.^65,66^

### 2.3 Spectroscopy Characterization

Infrared (IR) measurements were carried out with Bruker IR systems. For the spectroscopic analysis of solid samples, a Bruker AXS EQUINOX 55 FT-IR spectrometer was used. Spectra were recorded in the range of 4000 to 400 cm^–1^ with a resolution of 2 cm^–1^ in transmission mode. The samples were mixed with KBr and pressed into pellets. The spectral range covered wavenumbers from 7500 to 370 cm^–1^ with a resolution of 4 cm^–1^. A Horiba XPloRA PLUS system was used for Raman measurements from 70 to 4000 cm^–1^ with a spectral resolution of 2 cm^–1^. A 532 nm Laser with 100 mW was applied to record the Raman shift. Slit and hole features had each an aperture of 100 μm. An acquisition time of 5 s with 2 repetitions with a 2400 grading was preselected. Intensity was limited by setting filters appropriately to 10, 25, and 50%, respectively. The recorded intensity was detected through a 50× objective lens. The measured samples are shown in Figure S13.

All measured spectra were analyzed with the OPUS software 7.5 by Bruker. TA6 was used to fit the Raman data.

### 2.4 Thermal Analysis Characterization

Thermal analysis and differential scanning calorimetry (TG/DSC) were carried out with a Netzsch Jupiter F449 system being combined with MS (mass spectroscopy) for the simultaneous analysis of gaseous compounds being released during TG/DSC measurements. A heating rate of 10 K/min was preselected for the heating regime, which was set to a maximum temperature of 1250 °C. The Proteus software 6.1 by Netzsch was used to evaluate the recorded data.

Parallel to the TG/DSC measurement, mass spectra of the gaseous emissions were also simultaneously recorded during heating with a quadrupole mass spectrometer QMS 403 D Aëolos from Netzsch. The maximum atom mass units (amu) of the ion key fragments were set to 100 in the MS operating Quadstar software, i.e. a range from 1 to 100 amu was covered with 0.5 s as detection time for each mass. Hence, 145 cycles were recorded during the 2 h heating regime with a channeltron-type detector featuring a SEM (second electron multiplier) voltage of 1.2 kV.

### 2.5 Microscopy and Imaging

High-magnification images of zircon single crystals, used for Raman spectroscopy, were taken with a 3D digital microscope VHX 5000 system from Keyence. High-resolution scanning electron microscopy (HRSEM) coupled with energy dispersive X-ray spectroscopy (EDS) was carried out with a LEO 1530 VP Gemini system equipped with a field emission cathode and high-efficiency in-lens detector. The secondary electrons (SE) were detected at 15 kV with a working distance of 9 mm. Diagrams were drawn by use of Origin ver. 9.6.

## 3 Results

### 3.1 Microscopy and SEM/EDS

**Figure 1:**
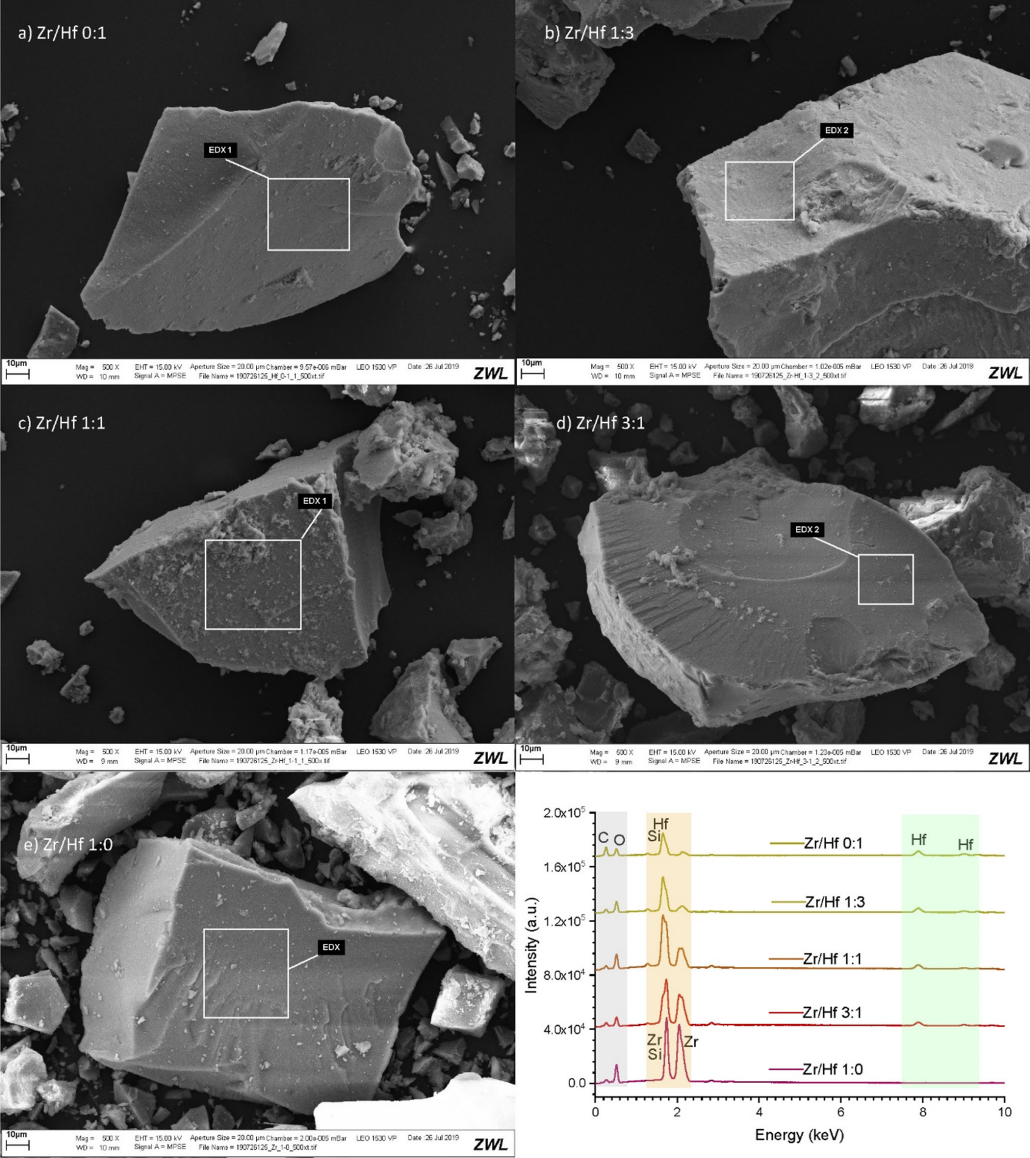
SE micrographs of the hydrothermal synthesized ZHSS. (a) Zr/Hf = 1:0, (b) Zr/Hf = 3:1, (c) Zr/Hf = 1:1, (d) Zr/Hf = 1:3, and (e) Zr/Hf = 0:1. Specific Zr and Hf lines in the EDS spectra are shown in (f) with highlighted ROI (pale orange and green).

The morphology of the hydrothermally synthesized solid solutions series was characterized with SEM. Micrographs of selected compositions are shown in Figure 1a–e. Figure 1f shows the respective EDS spectra. These samples did not feature or reveal any crystallographic faces, although high-resolution SEM was applied. EDS analysis of specific Zr and Hf energies was done semiquantitatively. However, by visual inspection of light, red and green regions of interest ((ROI) in Figure 1f Hf lines gained intensity, whereas Zr lines decreased systematically. Hence, the spectra at the bottom of the stack represent the zirconium-rich end-member and those at the top of the stack represent the hafnium-rich end-member.

### 3.2 X-ray Diffraction

For the crystallographic characterization of the zircon–hafnon solid solution, series single crystal analysis and powder X-ray diffraction (PXRD) were applied. Figure S11a–e shows the crystals which were used for the single crystal analysis. For each Zr/Hf composition, 1:0, 3:1, 1:1, 1:3, and 0:1, a small crystal was chosen and mounted on a glass capillary with a diameter of 0.1 mm. All selected crystals of the solid solution series were transparent and colorless. The results of this analysis are given in Table 1 and Figure 5.

**Table 1:** Results of the Single Crystal Analysis of the Zircon–Hafnon Solid Solution Series

Hf:Zr_ideal_	1:0	3:1	1:1	1:3	0:1
Hf:Zr_ideal_	0.000	0.250	0.500	0.750	1.000
Hf:Zr: measured^a^/weighed	0.008^a^	0.247	0.502	0.759	0.994^a^
Hf:Zr: refined Occ^b^	0.000	0.2018^b^	0.4543^b^	0. 74819^b^	1.000
*a*-parameter/Å	6.5929(6)	6.5828(5)	6. 5816(5)	6.5719 (4)	6.5688 (5)
*c*-parameter/Å	5.9709(8)	5.9642(7)	5. 9665(7)	5.9633 (6)	5.9608 (8)
Zr/Hf–O (II to *h*00)	2.1250(13)	2.1219(15)	2.1185(18)	2.1140(20)	2.1120(30)
Zr/Hf–O (II to 0*k*0)	2.2657(14)	2.2633(17)	2.2636(19)	2.2640(20)	2.2630(30)
Zr/Hf–O (average)	2.1954(14)	2.1926(16)	2.1911(19)	2.1890(20)	2.1875(30)
*c*/*a*	0.90566(15)	0.90603(13)	0.90654(13)	0.90739(11)	0.90744(70)
unit cell volume/Å^3^	259.53(5)	258.45(4)	258.45(4)	257.55(3)	257.20(4)
*y*(O)-position	0.06594(20)	0.06595(23)	0.06553(27)	0.06528(32)	0.06508(49)
*z*(O)-position	0.19543(20)	0.19554(29)	0.19520(32)	0.19539(41)	0.19541(53)
*R*_int_	0.0432	0.0447	0.0501	0.0659	0.0466
*R*_1_	0.0144	0.0128	0.0128	0.0124	0.0137
*wR*_2_	0.0389	0.0338	0.0301	0.0227	0.0339
GOF	1.392	1.151	1.295	1.264	1.542

a Determined by μXRF.

b Site occupancy calculated by Shelxl refinement.

The zircon–hafnon solid solution series had been characterized by single crystal analysis and PXRD. In Table 1, the results of single crystal analyses are listed. In addition to the ideal composition, this table also features the true composition, which has been determined with μXRF for the end-members, and the mixed compositions for the members in between. The latter are based on weighed amounts of the starting materials for the flux synthesis. For the intermediate solid solutions (3:1, 1:1, 1:3) the refined Zr/Hf occupancies are listed. The quality marks *R*_int_, *R*_1_, *wR*_2_, and the goodness of fit (GOF) are also listed.

Considering the lattice constants, the *a*-parameter decreased with the hafnium content. This tendency is also valid for the *c*-parameter, however, for the 1:1 mixture, the *c*-parameter is slightly larger compared to the 3:1 mixture. The opposite behavior is seen for the *c*/*a* ratio. The unit cell volume decreased more or less constantly with an increase in the hafnium amount. Figure 5 shows a graphical representation of these data and also features the results of the PXRD investigations of this solid solution series to be discussed later.

Some flux-grown crystals were selected from each fraction of the solid solution series and crushed for PXRD analysis because crystals with mixed Zr/Hf composition could feature zoning (cf. Figure 8, Tables S2–S7), which resulted in deviations from the expected composition for the 3:1 and the 1:1 mixtures (cf. Table 1). However, an excellent compositional match was observed for the 1:3 solid solutions. This destructive procedure was applied to homogenize a possible varying composition being present within single crystals featuring mixed composition–i.e. 3:1, 1:1, 1:3. Furthermore, a better statistical evaluation of the lattice parameters was possible, because powdered samples provide more crystallites, which contribute to the recorded intensity of Bragg reflections, contrary to single crystal measurements in which only one crystal contributes to Bragg reflections during a single run for data collection.

**Figure 2:**
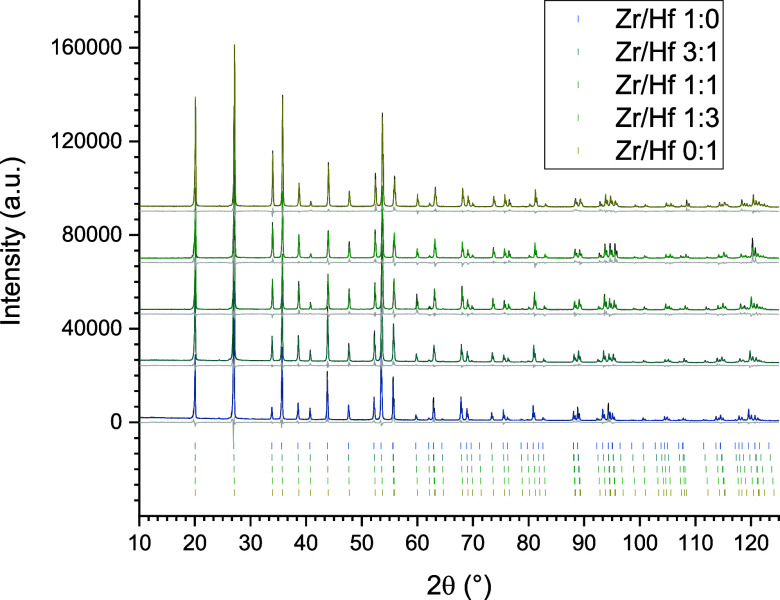
Rietveld plots (*I*_obs_: black, *I*_calc_: colored, difference: gray) of the crushed single crystals of the zircon-hafnon solid solution series. The colorization blue to green indicates the increase of the hafnium content with related (*hkl*) tick marks.

Nevertheless, the latter method is used for the determination of fractional coordinates of atomic positions. With respect to the zircon/hafnon structure (both isostructural (i.e., *I4*_*1*_*amd*, SG 141) only the Oxygen y- and z-coordinates (cf. Table 1) needed to be determined because Zr, Hf, and Si are located on special positions considering their fractional coordinates. The crushed samples were prepared on a silicon background free sample holder, measured, and evaluated by the Rietveld Method (cf. refs (66,65)), Figure 2 shows the obtained Rietveld plots of the solid solution series in a stacked manner, i.e. the color shift from blue to green the increase of the hafnium content, upward for the stack of graphs, and downward for the tick mark stack. By visual inspection, the respective diffractograms of each composition were single-phased. The evaluation of the refined lattice parameters, which are listed in Table 2, showed a continuous decrease of the *a*- and *c*-parameters of the solid solution series with an increase in the hafnium content. The same behavior was also valid for the unit cell volume. A linear but opposite trend was observed for the *c*/*a* ratio, which increased with the hafnium content. This tendency was also observed for the single crystal data (cf. Figure 5, Table 1). The Rwp values given in Table 2, range from 5.13 to 8.09 and so demonstrate a good quality.

**Table 2:** Results of the Rietveld Analysis Including Refined Lattice Parameters of the Zircon–Hafnon Solid Solution Single Crystals Crushed for PXRD Investigation, Unit Cell Volume, *c*/*a* Ration, and *R*_wp_ Values

Hf:Zr_ideal_	1:0	3:1	1:1	1:3	0:1
Hf:Zr_ideal_	0.000	0.250	0.500	0.750	1.000
Hf:Zr: measured^a^/weighed	0.008^a^	0.247	0.502	0.759	0.994^a^
*a*-parameter/Å	6.60402(4)	6.59480(5)	6.58755(5)	6.58078(7)	6.57453(5)
*c*-parameter/Å	5.97928(6)	5.97584(8)	5.97205(7)	5.96957(10)	5.96560(8)
*c*/*a*	0.90540(1)	0.90614(1)	0.90657(1)	0.90712(2)	0.90738(1)
unit cell volume/Å^3^	260.775(4)	259.898(5)	259.161(5)	258.522(6)	257.860(5)
*R*_wp_	7.65	5.13	6.32	8.09	5.11

a Determined by μXRF.

The evident systematic behavior of the lattice parameters, *c*/*a* ratio, and the unit cell volume of the crushed single crystals is also shown in Figure 5, which shows hydrothermal, single crystal (black lines and symbols), and PXRD data of the solid solution series (red lines and symbols).

**Figure 3:**
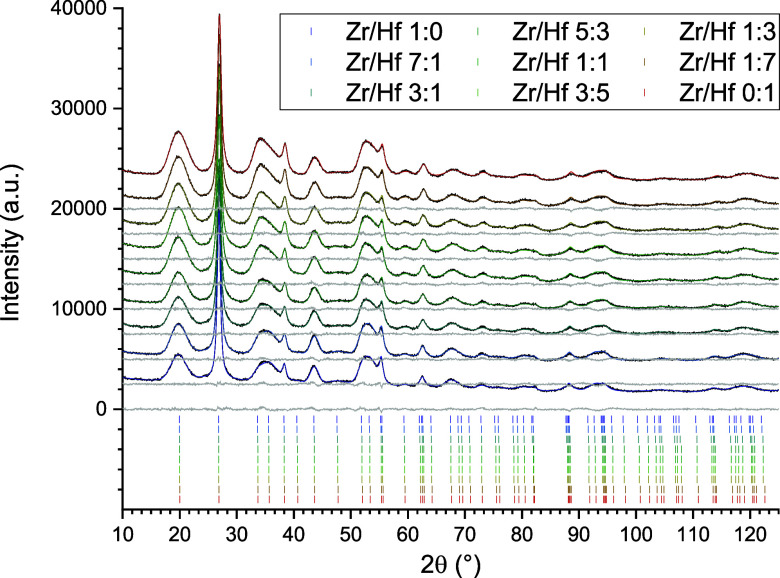
Rietveld plots (*I*_obs_: black, *I*_calc_: colored, difference: gray) of the hydrothermally synthesized zircon–hafnon solid solution series. The color shift blue to red indicates the increase of the hafnium content and their related (*hkl*) tick marks.

Figure 3 shows the Rietveld plots of the zircon-hafnon solid solution series, synthesized via a hydrothermal route in 7 days at 200 °C. The stacking order of the tick marks and the displayed diffractograms follows the scheme of Figure 2 (displaying the diffractograms of the crushed single crystals). However, the hydrothermal route features four more compositions for the zircon-hafnon solid solution series, i.e., 7:1 (*x*(Hf) = 0.125), 5:3 (*x*(Hf) = 0.375), 3:5 (*x*(Hf) = 0.625), and 1:7 (*x*(Hf) = 0.875). Thus, nine mixtures were in total produced and evaluated by PXRD analysis. The true composition also features deviations from the ideal ratios. In this case, these differences are due to the determined LOI of the starting materials for the hydrothermal synthesis, indicating deviations of the given composition for the starting material. The atomic positions of the intermediates 7:1 (0.125), 5:3 (0.375), 3:5 (0.625), and 1:7 (0.875) were the *y*- and *z*- coordinates of the single crystal analysis of the zircon–hafnon series which have been determined for the mixtures 3:1 (0.25), 1:1 (0.5), and 1:3 (0.75). The Zr/Hf ratio relates to the true (weighed) composition of the hydrothermal series (cf. Table 3).

**Table 3:** Results of the Rietveld Analysis–Refined Lattice Parameters of the Hydrothermally Synthesized Zircon–Hafnon Solid Solution Series

Hf:Zr_ideal_	01:00	07:01	03:01	05:03	01:01	03:05	01:03	01:07	00:01
Hf:Zr_ideal_	0.000	0.125	0.250	0.375	0.500	0.625	0.750	0.875	1.000
Hf:Zr: measured^a^/	0.008^a^	0.146	0.293	0.421	0.550	0.670	0.778	0.892	0.994^a^
weighed									
*a*-parameter/Å	6.64667(53)	6.64311(54)	6.63816(62)	6.63650(72)	6.63322(66)	6.63356(61)	6.62987(62)	6.62742(61)	6.62624(62)
*c*-parameter/Å	5.98212(192)	5.98149(188)	5.97417(234)	5.96636(228)	5.96554(234)	5.96196(230)	5.96201(245)	5.96014(234)	5.95575(242)
*c*/*a*	0.90002(30)	0.90004(29)	0.89997(36)	0.89902(36)	0.89934(36)	0.89876(36)	0.89927(38)	0.89932(36)	0.89881(38)
unit cell volume/Å^3^	264.279(1)	264.968(1)	263.253(1)	262.777(1)	262.481(1)	262.351(1)	262.061(1)	261.785(1)	261.499(1)
*R*_wp_	2.41	2.09	1.92	1.83	1.77	1.80	1.79	1.78	1.81

a Same composition as single crystal synthesis (determined by μXRF) due to the same starting materials.

Due to the strong peak broadening, the Rietveld fitting routine was additionally modified to account for the strong anisotropy of the observed *hkl* reflections −13 different types of *hkl*-dependent crystallite sizes (in nanometres) were refined individually and independently from each other in order to account for the anisotropic feature of the hydrothermal reaction products (cf. Supporting Information: Table S11b). Furthermore, a peak phase was introduced, because the 101 reflection was not fitted accurately. This additional feature accounts for the presents of amorphous phases, which could not be observed, yet produce an amorphous hump. Heat treatments up to 1250 °C of the zirconium-rich end-member for several hours revealed the presence of crystalline cristobalite and ZrO_2_ (cf. Supporting Information: Figure S9). This observation was the reason for the introduction of a “peaks phase”. The difference curve representing the *I*_obs_ – *I*_calc_ did not feature strong amplitudes and the respective *R*_wp_ values listed in Table 3 were ≤2.63. The *c*/*a* ratio showed a clear trend and decreased mainly continuously with the increase of the Hf content. Considering the *a*- and *c*-parameters and the unit cell volume, these parameters decreased even more clearly with the decrease of the Zr content (cf. Table 3, Figure 5).

This behavior is displayed graphically in Figure 5 (blue lines and symbols), which also features the variation of the lattice constants of the hydrothermal synthesis reaction products, which were analyzed by TG (green lines and symbols).

**Figure 4:**
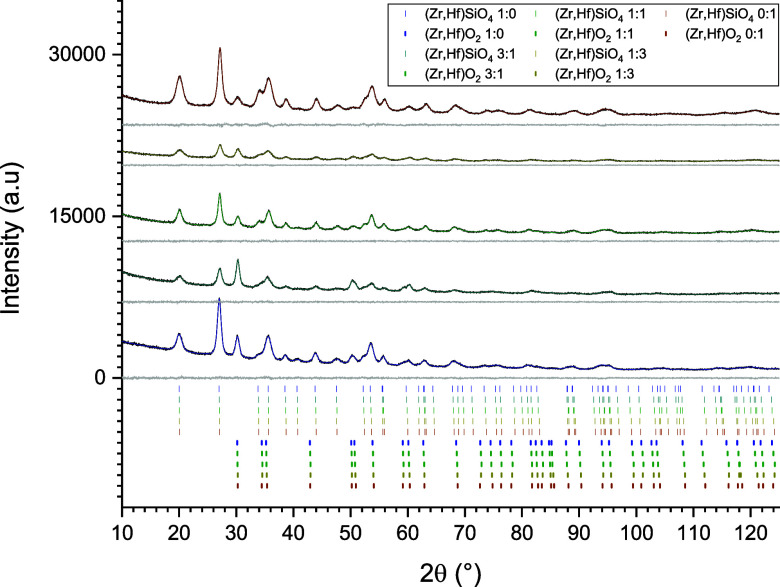
Rietveld plots (*I*_obs_: black, *I*_calc_: colored, difference: gray) of the hydrothermally synthesized zircon-hafnon solid solution series, heated to 1250 °C during TG analysis. The block (shift from blue to red) indicates the increase of the hafnium content and related (*hkl*) tick marks. The upper 5 lines relate to zircon-hafnon *hkl* reflections. The lower 5 lines belong to the Zr_1–*x*_Hf_*x*_O_2_ compounds.

Figure 4 shows the respective Rietveld plots of the hydrothermal reaction products, which were analyzed additionally with TG. The stacking order of diffractograms and tick marks follows the identical scheme that had been applied for Figures 2 and 3. However, these diffractograms were not single-phased anymore because tetragonal solid solutions of Zr_1–*x*_Hf_*x*_O_2_ crystallized upon the heat treatment and were hence observed as a second phase (red tick marks in Figure 4). Structures of tetragonal ZrO_2_^67^ and HfO_2_^68^ were used for the QPA.

**Figure 5:**
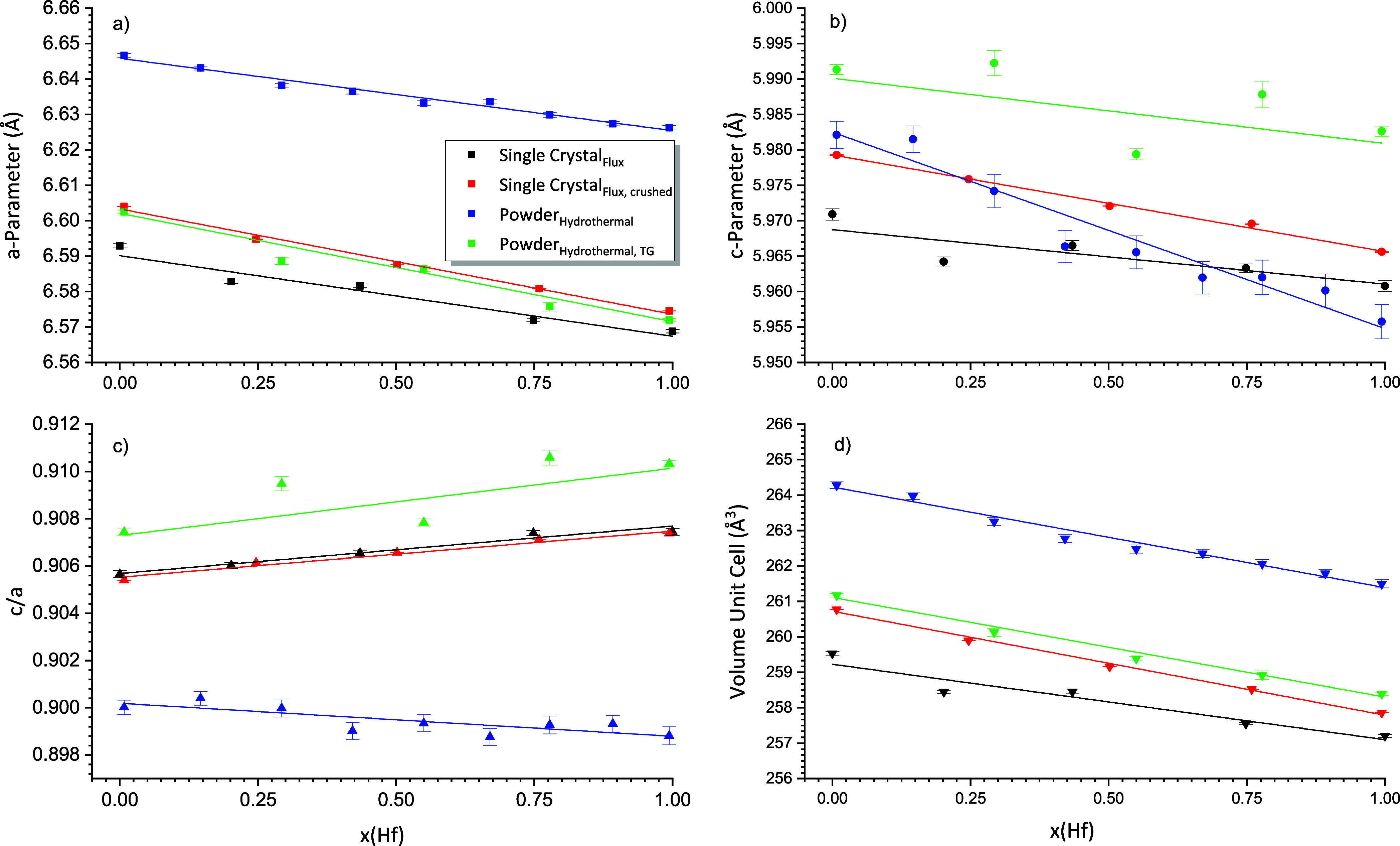
Comparison of lattice parameters (a) *a*-parameter, (b) *c*-parameter), *c*/*a* ratio (c)), and unit cell volume (d)) of the zircon-hafnon solid solution series considering the different XRD analysis: Single crystal analysis–black lines and symbols, crushed-to-powder single crystals–red lines and symbols, hydrothermal synthesis–blue lines and symbols, and TG-heated samples of the hydrothermal synthesis–green lines and symbols. Lines are fitted by linear functions.

The applied Rietveld refinement (cf. Table 4) revealed an alternating pattern for the c-parameter as well as for the *c*/*a* ratio. However, the respective overall trends decreased and increased with the hafnium content, respectively (cf. Figure 5). The a-parameter and the unit cell volume decreased systematically with increasing hafnium content. The *R*_wp_ featured low values ≤4.46.

**Table 4:** Results of the Rietveld Analysis–Refined Lattice Parameters of the Hydrothermally Synthesized Zircon–Hafnon Solid Solution Series, Which Were Heated to 1250 °C during TG Analysis

Hf:Zr_ideal_	1:0	3:1	1:1	1:3	0:1
Hf:Zr_ideal_	0.000	0.250	0.500	0.750	1.000
Hf:Zr: measured^a^/weighed	0.008^a^	0.293	0.550	0.778	0.994^a^
*a*-parameter/Å	6.60248(54)	6.58865(102)	6.58639(62)	6.57577(120)	6.57193(48)
*c*-parameter/Å	5.99133(70)	5.99226(177)	5.97937(80)	5.98782(180)	5.98262(7154)
*c*/*a*	0.90744(13)	0.90948(30)	0.90784(15)	0.91059(32)	0.91033(13)
unit cell volume/Å^3^	261.179(4)	260.125(5)	259.388(5)	258.918(6)	258.391(5)
*R*_wp_	2.96	3.26	3.09	4.46	2.83

a Same composition as single crystal synthesis (determined by μXRF) due to the same starting materials.

Additionally, the refinement yielded the phase quantities of the observed zircon–hafnon and Zr–Hf-oxide solid solution series (Table 5). The determined amounts did not follow a constant trend; instead, an alternating pattern was observed with the increase of the hafnium content, and it correlates with the *c*-parameter and with the *c*/*a* ratio listed in Table 4.

**Table 5:** Quantification of the Reaction Products of the Hydrothermally Synthesized Zircon–Hafnon Solid Solution Series Being Formed during TG Analysis at 1250 °C

Hf:Zr_ideal_	1:0	3:1	1:1	1:3	0:1
Hf:Zr_ideal_	0.000	0.250	0.500	0.750	1.000
Hf:Zr: measured^a^/Weighed	0.008^a^	0.293	0.550	0.778	0.994^a^
Zr_1–*x*_Hf_*x*_SiO_4_	84.39(0.84)	63.24(2.55)	86.87(2.92)	77.51(3.85)	91.66(1.12)
Zr_1–*x*_Hf_*x*_O_2_	15.61 (0.84)	36.77(2.55)	13.13(2.92)	22.49(3.85)	8.34(1.12)

a Same composition as single crystal synthesis (determined by μXRF) due to the same starting materials.

From all XRD measurements, Figure 5 summarizes graphically the behavior of the lattice parameters and derived parameters like the *c*/*a* ratio and the unit cell volume in dependence of the hafnium amount of the synthesized solid solution series.

The *a*-parameter decreased, irrespective of the XRD method and the synthesis route. However, the *a*-parameter of the hydrothermal series (*T*_applied_ = 200 °C) is significantly larger compared to the single crystal and TG series (*T*_applied_ ≥ 1250 °C). For the c-parameter, the situation revealed a different behavior for the hydrothermal samples (blue symbols), which have been subjected to thermal analysis (green symbols) – for the latter, a distinct scatter is evident. Linear fitting procedures of the *c*-parameter resulted in lower *R*^2^ values (cf. Supporting Information: Table S10) for these two series. The slope for the hydrothermal series (blue line) is distinctly more negative than the other fitted slopes (Single Crystal–black line, crushed single crystal–red line, and TG–green line) all exhibiting similar slopes.

The *c*/*a* ratio (Figure 5c) shows the strong scatter for the hydrothermal (blue triangles) and hydrothermal TG analysis (green triangles) of the zircon–hafnon solid solution series, yet the linear fits for the single crystals and for the hydrothermal series, which has been subjected to TA also featured a positive slope. A negative slope was observed only for the hydrothermally synthesized solid solution series. For the (crushed) single crystals better *R*^2^ values were obtained in comparison to hydrothermal and TG data. The fit results for the unit cell volume (Figure 5d) for four XRD measurement regimes were quite consistent because each fit revealed a negative slope and the *R*^2^ values did not vary to such an extent as for the other parameters (*a*, *c*, and *c*/*a*). Furthermore, the unit cell volume is significantly larger for the hydrothermal zircon-hafnon series.

Although a linear fit is convenient for comparison of the four ZHSS, we observed a negative deviation (Figure S12) from linear behavior considering the crushed series (red symbols in Figure 5).

### 3.3 Raman and μXRF

**Figure 6:**
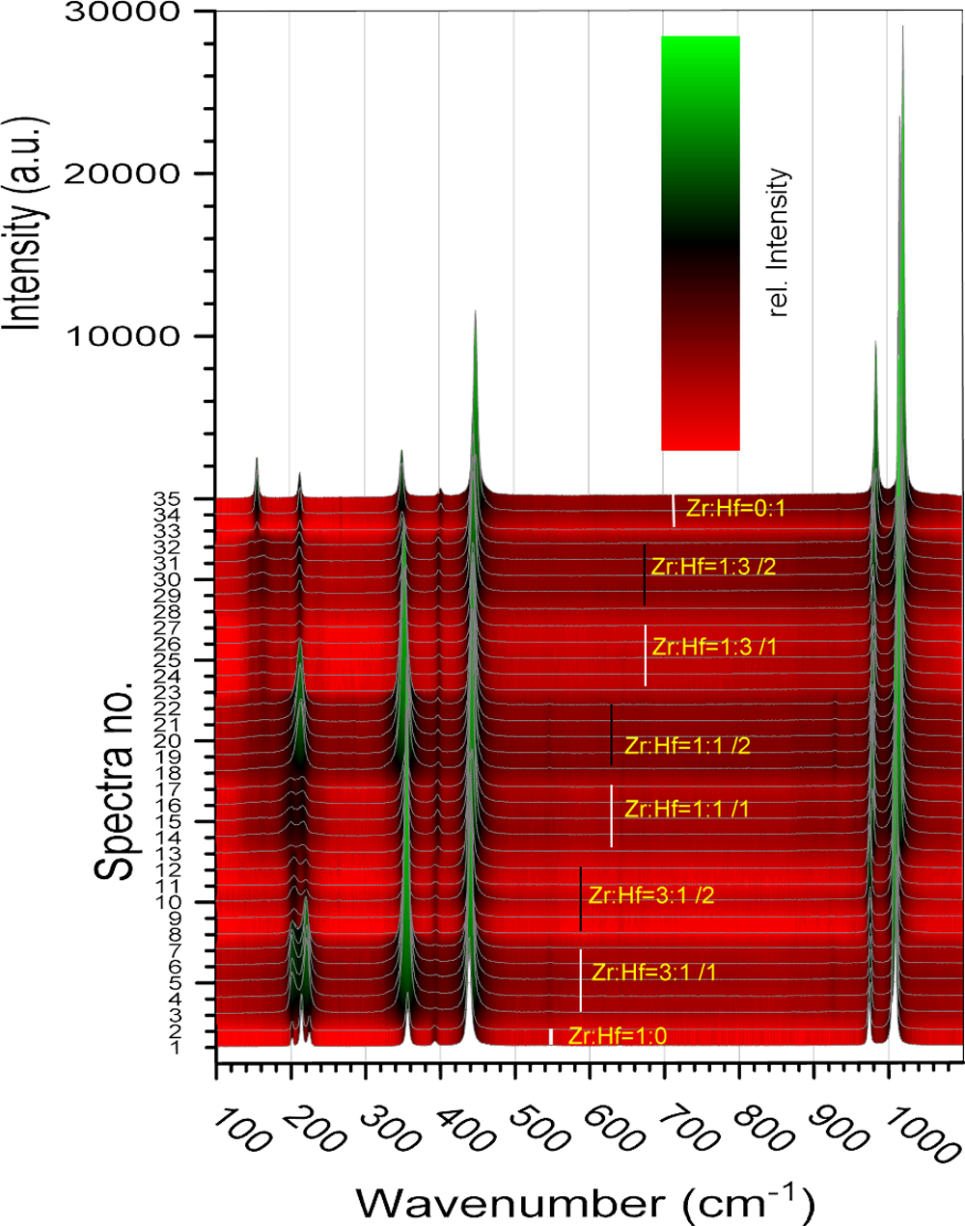
Raman spectra of the single crystal zircon–hafnon solid solution series. Black and white bars indicate the recorded spectra of specific compositions of the ZHSS series.

**Figure 7:**
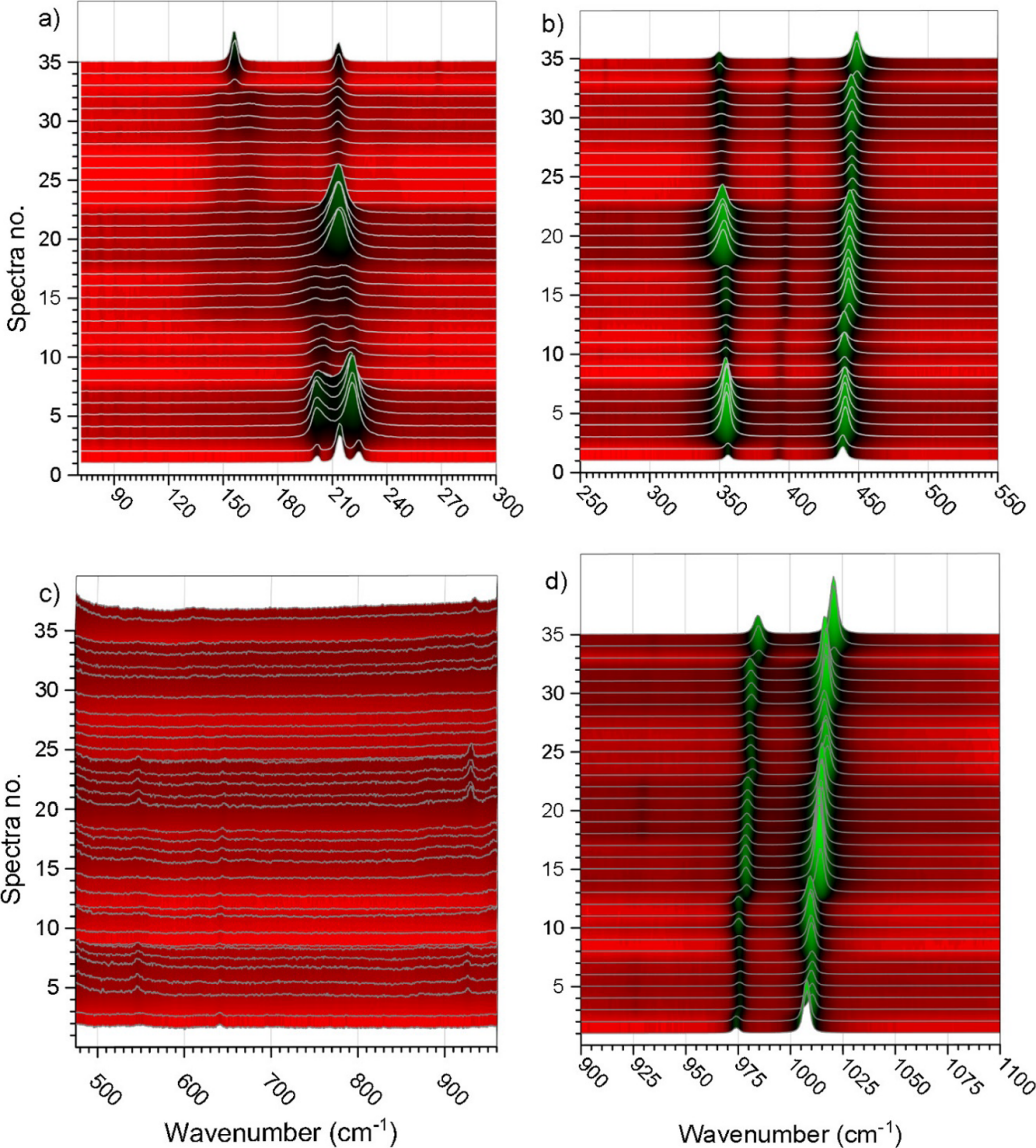
(a–d) Selected enlarged ROI of the Raman spectra of the single crystal ZHSS.

**Figure 8:**
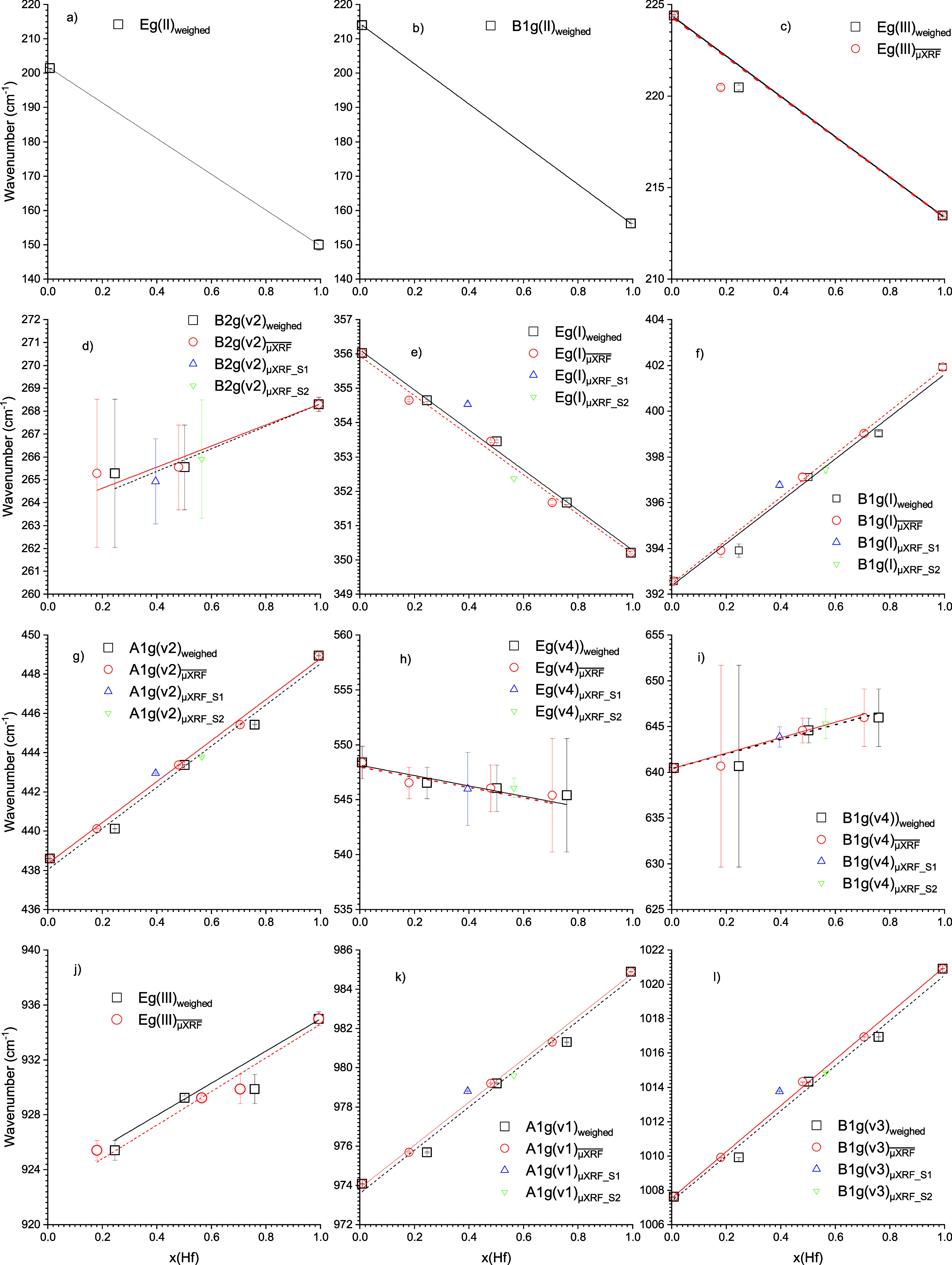
(a–l) Plots of the observed Raman modes of the single crystal ZHSS with the composition *x*(Hf). Black symbols and lines represent linear fits with respect to the weighed ZrO_2_/HfO_2_ ratio for the intermediate compositions Zr/Hf = 3:1, 1:1, 1:3. Red symbols are linear fitting procedures related to Zr/Hf ratios determined by μXRF. Green and blue symbols relate to the Zr/Hf ratio determined by μXRF of the two different zircon-hafnon single crystals with an assumed ideal 1:1 ratio.

To characterize this solid solution series with Raman Spectroscopy (cf. Figures 6–8, Table 6) and μXRF (cf. Supporting Information: Figures S1–S8, Tables S1–S8), larger individuals of the solid solution series were selected and glued onto ordinary sample holders used for microscopy. Pictures of the whole crystals of this selection were taken with a digital microscope and are shown in Figure S13. For the end members −1:0 (a)), 0:1 (e)) – only one crystal has been selected for each composition. With respect to the crystal with mixed compositions −3:1 (b1/b2)), 1:1 (c1/c2), 1:3 (d1/d2) – two crystals were selected for each composition. These individuals, which featured larger dimensions than those selected for the single analysis, were also transparent and colorless.

**Table 6:** Averaged Wavenumbers of the Observed Raman Modes of the Single Crystal Zircon–Hafnon Solid Solution Series

Zr/Hf_ideal_	1:0	3:1	1:1	1:3	0:1
Zr/Hf_weighed_	0.000	0.247	0.502	0.759	1.000
Zr/Hf_μXRF_	0.008	0.182	0.481	0.706	0.994
*E*_g_(II)	201.41	SM^a^	SM^a^	SM^a^	149.99(1.60)
*B*_1g_(II)	213.98(2)	SM^a^	SM^a^	SM^a^	156.21(4)
*E*_g_(III)	224.40(4)	220.47(13)	SM^a^	SM^a^	213.46(4)
*B*_2g_(ν2)	n.d	265.28(3.24)	265.54(1.85)	n.d	268.30(31)
*E*_g_(I)	356.03(3)	354.64(3)	353.45(4)	351.67(6)	350.21(3)
*B*_1g_(I)	392.56(10)	393.91(29)	397.11(15)	399.02(11)	401.92(13)
*A*_1g_(ν2)	438.60(2)	440.12(2)	443.37(2)	445.42(2)	448.94(2)
*E*_g_(ν4)	548.39(1.48)	546.52(1.44)	546.04(2.11)	545.40(5.16)	n.d
*B*_1g_(ν4)	640.45(53)	640.66(11.02)	644.58(1.36)	645.95(3.14)	n.d
*E*_g_(ν3)	n.d	925.41(72)	929.22(14)	929.87(1.06)	934.99(51)
*A*_1g_(ν1)	974.08(2)	975.68(2)	979.20(2)	981.31(2)	984.89(1)
*B*_1g_(ν3)	1007.61(1)	1009.91(1)	1014.32(1)	1016.94(1)	1020.91(1)

a SM, supporting mode.

Figure 6 shows the Raman spectra, which have been recorded. Due to the purity of the solid solutions end-members, only one zircon and one hafnon crystal were chosen and so feature only one and three recorded spectra, respectively.

With respect to the intermediate compositions 3:1, 1:1, and 1:3, two crystals were chosen for analysis, and each has been measured 5 times. 34 spectra have been recorded for the complete solid solution series. For the graphical display in Figures 6 and 7, the zircon end-member has been plotted twice (spectra no. 1 and 2).

Figure 7a–d depicts the enlarged ROI of Figure 6. These magnified depictions helped to identify and select modes used for fitting to determine the position of bands more precisely (cf. Table 6). Especially in the region 70 to 300 cm^–1^, the selection of modes was very challenging for the intermediate composition, because a clear systematic shift of modes was not observed for these intermediate compositions 3:1, 1:1, 1:3. Hence, only the end-members–zircon and hafnon–allowed an unambiguous selection of modes. With respect to the transition of the compositions 1:0 to 3:1 the observed modes seemed to broaden and to merge from 3 (approximately 201, 214, and 224 cm^–1^) into 2 modes (spectra no. 1 to 12) at approximately 201 and 216 cm^–1^. Furthermore, the spectra no. from 8–12 (the second sample of the 3:1 composition) feature the same modes that were observed on the first 3:1 sample, yet the intensity was significantly reduced. These 2 modes seemed to flatten even more considering the transition from 3:1 to 1:1 (spectra no. 13–17) and also featured a slight shift toward smaller wavenumbers. However, the evaluation became more complicated by analyzing the second crystal with the 1:1 composition (spectra no. 18–22). These two flat modes merge visually into one mode with an increased intensity at approximately 213 cm^–1^. Such could be explained by the different chemical composition determined by μXRF (cf. Figure 8, Tables S4–S6), despite the same nominal 1:1 ratio.

For the transition from 1:1 (sample 2, spectra no. 18–22) to 1:3 (sample 1, spectra no. 23–27), the latter mode remained visually unchanged, yet lost intensity, which is the same for both investigated samples of the 1:3 composition. Furthermore, both of the 1:3 samples feature two broad modes at approximately 150 and 165 cm^–1^. However, their observed intensity was weak and diffuse compared to the spectra of the 1:1 sample. To summarize the situation in this ROI, the *E*_g_(II), *B*_1g_(II), and *E*_g_(III) modes were only identified for the end-members of the ZHSS. Furthermore, the mode assignment for hafnon was not as clear as it was for zircon. This circumstance will be discussed later.

The second ROI (Figure 7b) shows wavenumbers ranging from 250 to 550 cm^–1^. By visual inspection, the modes *E*_g_(I), *B*_1g_(I), and *A*_1g_(ν2) were easy to identify for all investigated samples and featured clearly a dependence on the hafnium content. However, the *B*_2g_(ν2) mode was only visible for the 3:1, 1:1, and 0:1 compositions. As shown in Figure 7c, the wavenumber ROI between 470 and 970 cm^–1^ is strongly enhanced in intensity because the *E*_g_(ν4), *B*_1g_(ν4), and the *E*_g_(ν3) modes were barely detectable. These modes seemed to be very weak and were not always observed, e.g., *E*_g_(ν4) was not present in the 0:1 sample and, furthermore, its observable shift (cf. Table 6) seemed not to vary steadily with respect to the transition from composition 1:0 to 3:1.

A comparable behavior could be observed for the *B*_1g_(ν4) mode, which was not visible for the 0:1 composition. A distinct shift jump could be observed for the transition from 3:1 to 1:1 composition. The *E*_g_(ν3) mode seemed to behave likewise because its band was not present for the 1:0 composition and a strong hiatus was observed for the transition from 3:1 to 1:1 and from 1:3 to 0:1 depending on the hafnium content. In contrast, in the range from 900 to 1100 cm^–1^, the modes *A*_1g_(ν1) and *B*_1g_(ν3) were very strong and seemed to shift constantly and gradually with the increase of hafnium. Table 6 comprises the observed and averaged fitted modes used to for a second fitting procedure, which was carried out, to evaluate the dependencies of the shifts from the composition (cf. Figure 8), i.e. on the hafnium amount.

The mean values for the modes with mixed composition (3:1, 1:1, 1:3) in Table 6 were generally obtained from 5 measurements of each from two samples (cf. Figure S13, Table S14–S22). However, this procedure could not be applied in every case, because the modes were sometimes not observed (e.g., the *E*_g_(ν3) mode of sample 1 for the 1:1 composition and for *E*_g_(ν4) mode sample 2 for the 1:3 composition). Furthermore, to improve the fit quality for the full spectrum, despite the unambiguous mode situation for the mixed composition smaller than approximately 240 cm^–1^, a work-around was applied by introducing some useful supporting modes (cf. Table 6). In this context, it remains an open question whether the mode for the 3:1 composition at 220.47 cm^–1^ is a true *E*_g_(III) or only a supporting mode. The shift of observed modes depending on the composition (i.e., on the hafnium content) of the zircon-hafnon solid solution series is shown in Figure 8a–l. Fitting procedures were based on the methods applied for the determination of the Zr and Hf compositions. Two methods were considered: The first was based on averaged results obtained by μXRF analysis (red dotted line and red symbols) of the flux-grown crystals of the complete solid solution series shown in Figure S13 (cf. Tables S1–S8, cf. Figures S1–S8), the second (solid black line and black symbols) considered the results of the μXRF analysis for the end-member zircon and hafnon, however, the mixed compositions 3:1, 1:1, and 1:3 were based on the mass of the starting materials ZrO_2_ and HfO_2_ determined by weighing. The graphs shown in Figure 8 feature also blue and green symbols, which refer to sample 1 and sample 2 of the 1:1 composition. These 2 samples were also plotted (yet not considered for fitting) because the μXRF analysis of each sample revealed significantly different compositions based on the hafnium content–sample 1: *x*(Hf) = 0.396, sample 2: *x*(Hf) = 0.565, *x*_mean_ = 0.481. In contrast, the mixed compositions 3:1 and 1:3 were quite homogeneous for both samples for each composition. Consequently, for determination of the mean values the average value of each crystal was considered (cf. Supporting Information: Tables S13–S23). The reason to apply two different fitting procedures is that μXRF and the Raman analysis were applied on the surface of the crystals, which could possibly feature zoning, especially for the mixed compositions 3:1, 1:1, and 1:3.

μXRF and Raman measurements were carried out on the same faces. It was not sufficient to rely only on the weighed amount of the starting materials. To evaluate the quality of these two methods, the *R*^2^ was determined. In six out of ten linear fits, the *R*^2^ is better for the μXRF measurements (cf. Supporting Information: Raman–Linear Mode Fit). However, as Figure 8c–l shows, the differences were very small. Consequently, both methods provide useful information for zoning effects.

### 3.4 IR Spectroscopy

**Figure 9:**
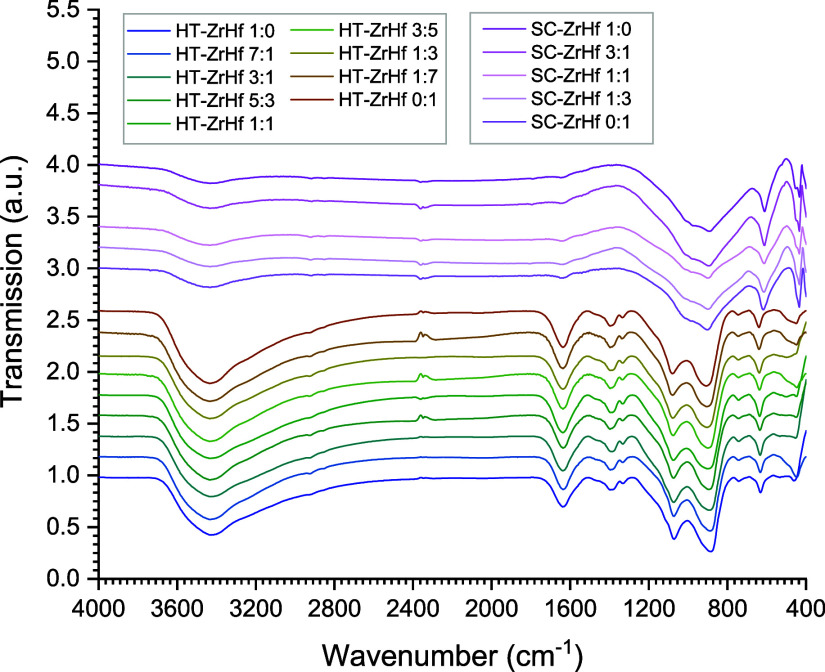
IR spectra of the crushed single crystals (SC: Top five stack) and hydrothermal products of the zircon-hafnon solid solution series (HT: Bottom nine stack).

Besides Raman spectroscopy, IR spectroscopy was also applied to characterize the zircon-hafnon solid solution series. These investigations comprised the flux-grown and hydrothermally produced series as well. First, the powder of crushed single crystal was used. The five spectra thereof are shown in Figure 9 on top of the stack. The nine graphs of the hydrothermal solid solution series are represented at the bottom of the stack. All spectra are displayed in transmission mode. To determine the band position, the first derivatives were applied for the observed minima and the second derivatives for the shoulders.

**Figure 10:**
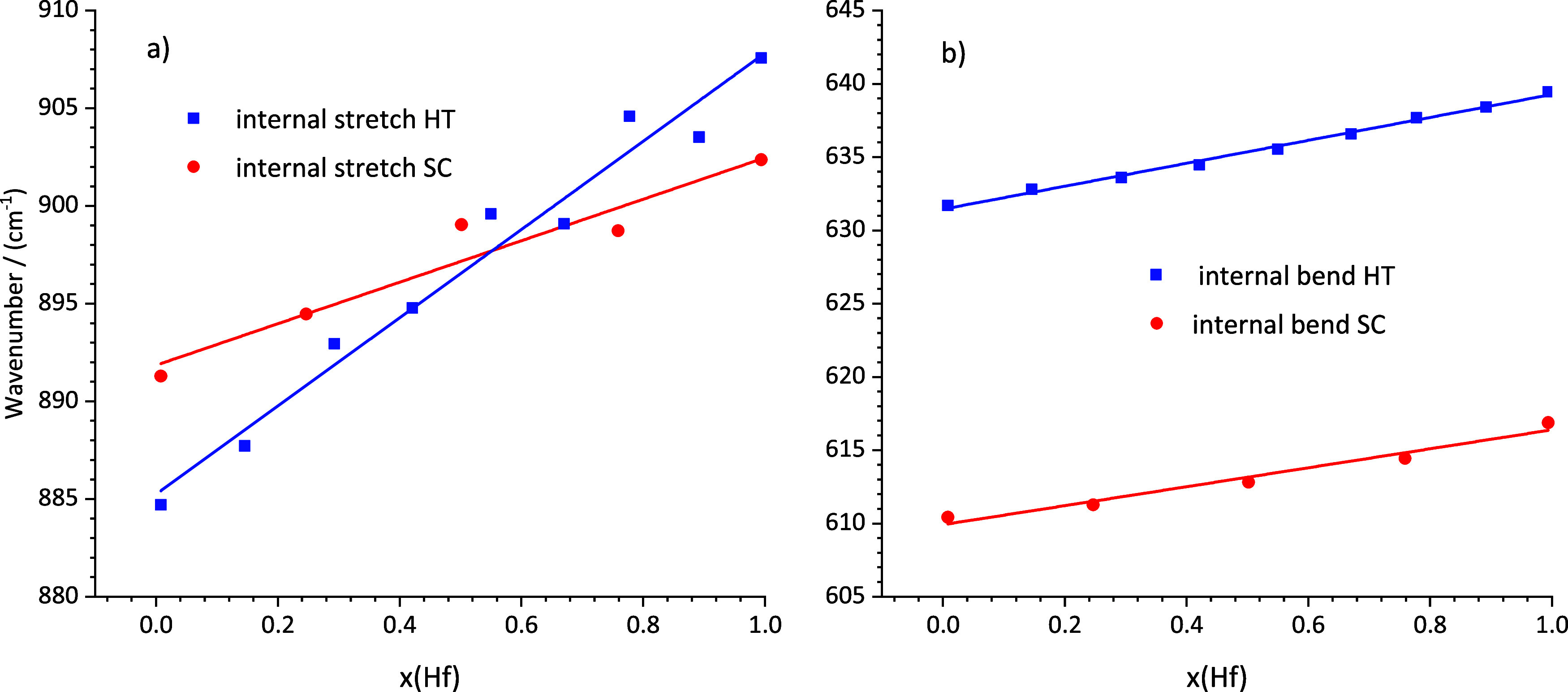
Comparison of selected IR modes of the crushed single crystals and hydrothermal synthesis of the zircon–hafnon solid solution series vs composition *x*(Hf) (a) Comparison of internal asymmetric stretching modes ν3 *E*_u_ and (b) internal asymmetric bending modes (ν4 *A*_2u_).

The results are listed in Table 7 (flux-grown solid solution series) and Table 8 (hydrothermally synthesized solid solution series). Graphical representations, which also feature linear fits, are shown in Figure 10 (flux grown series: red lines and symbols, hydrothermal series: blue lines and symbols). Differences between these two series could be spotted immediately. The broad band at approximately 3430 cm^–1^ was present in both series and could be ascribed to vibrational stretching modes of water, but it was much more intense for the hydrothermal series. The same is also valid for the band at approximately 1640 cm^–1^ belonging to the bending modes of H_2_O.

**Table 7:** Observed IR Modes of the Crushed Single Crystals of the Zircon–Hafnon Solid Solution Series

ideal	1:0	3:1	1:1	1:3	0:1
	0.000	0.250	0.500	0.750	1.000
measured^a^/weighed	0.008^a^	0.247	0.502	0.759	0.994^a^
overtone	1791.61	1794.92	1798.79	1807.09	1810.46
shoulder^b^	1006.76	1008.52	1009.27	1010.88	1010.83
int. as. stretch. ν3 *A*_2u_^b^	970.59	974.91	978.00	981.01	984.38
int. as. stretch. ν3 *E*_u_	891.28	894.46	899.03	898.73	902.36
int. as. bend. ν4 *A*_2u_	610.42	611.28	612.80	614.44	616.88
ext.	451.22	447.40	445.69^b^	444.23^b^	443.58^b^
int.as.bend. ν4 *E*_u_	433.75	434.22	434.39	434.48	434.64

a Determined by μXRF.

b Shoulder.

**Table 8:** Observed IR Modes of the Hydrothermal Reaction Products of the Zircon–Hafnon Solid Solution Series

ideal	0.000	0.125	0.250	0.375	0.500	0.625	0.750	0.875	1.000
measured^a^/weighed	0.008^a^	0.146	0.293	0.421	0.550	0.670	0.778	0.892	0.994^a^
Si–O–Si: as. stretch.	1071.67	1072.42	1073.33	1074.28	1074.44	1076.67	1077.27	1079.26	1079.61
int. as. stretch. ν3 *E*_u_	884.70	887.74	892.94	894.80	899.60	899.05	899.05	903.48	907.58
Zr_1–*x*_Hf_*x*_O_2_ mcl.	742.36	742.88	743.19	743.84	743.46	743.70	744.34	744.85	745.08
int. as. bend. ν4 *A*_2u_	631.66	632.77	633.59	634.45	635.54	636.56	637.66	638.40	639.43
int. as. bend. ν4 *E*_u_	nd	446.30	nd	nd	nd	446.53	nd	448.67	450.37

a Same composition as SC synthesis (determined by μXRF) due to the same starting material.

The presence of this band for the flux-grown series could be ascribed to the water content of KBr, which was used as a matrix for the IR pellets (cf. Supporting Information: Figure S14). The IR modes of the flux-grown series of the end-members zircon and hafnon all observation could be related to the composition determined by μXRF. The mixed compositions 3:1, 1:1, and 1:3 refer to the weighed ZrO_2_/HfO_2_ ratio of the starting materials because the single crystals were crushed to powder for the IR analysis and a possible zoning of these crystals was leveled out by the crushing procedure. Such an assumption was not necessary for the end-members.

For the observed wavenumbers, all observations seemed to correlate with an increase in the hafnium content. The first observed minimum was at a wavenumber of approximately 1800 cm^–1^. These minima (cf. Table 7) were very weak and could possibly represent some overtones of the vibrational minima at approximately 900 cm^–1^, which were very strong, and represent absolute minima. These bands are the internal asymmetric stretching modes ν3 *E*_u_. Their wavenumber increased with the increase of the hafnium content. The same is also valid for the shoulders at approximately 977 cm^–1^ ranging approximately from 971 to 984 cm^–1^. These bands could be assigned as internal asymmetric stretching modes ν3 *A*_2u_.

The ROI at 1009 cm^–1^ exhibits also shoulders with the modes increased with the hafnium content from approximately 1007 to 1011 cm^–1^. However, a clear mode assigned was not possible.

Two more minima were present at approximately 434 and 613 cm^–1^. These modes could be assigned as internal bending of ν4 *E*_u_ and *A*_2u_. Between these minima, additional modes at approximately 447 cm^–1^ could be observed. For the 1:0 (pure zircon) and 3:1 composition, these modes are observed as true minima whereas from 1:1 to 0:1 only shoulders were observed which weakened with the increase of the hafnium content. In contrast to the other minima and shoulders, their wavenumber shifts decreased from approximately 451 to 444 cm^–1^ with the increase of the hafnium content of the solid solution series.

The evaluation of the hydrothermally synthesized ZHSS revealed four modes, which feature a dependence on the hafnium content. The results are summarized in Table 8. All wavenumbers increase with the content of hafnium. By comparing these modes with modes of the flux-grown series two wavenumber regions can be related to the latter series: 900 and 635 cm^–1^.

The difference of the first ROI is that the flux-grown series covered a range of approximately 11 cm^–1^ (891.30 to 902.34 cm^–1^) whereas the hydrothermal series ranges from 884.71 to 907.56 cm^–1^ and covered thus a range, which is slightly more than twice as large (cf. Figure 10a). However, the hydrothermal internal asymmetric bending modes at 635 cm^–1^ (ν4 *A*_2u_), cover approximately 8 wavenumber units (from 631.66 to 639.43 cm^–1^) comparable to the flux grown series, which ranges from 610.41 to 616.88 cm^–1^. Furthermore, this interval was shifted by approximately 20 cm^–1^ to higher wavenumbers compared to the flux-grown series (Figure 10b).

The other two observed ROIs at 1075 and 743 cm^–1^ did not have respective analogues in the flux-grown series. The range between 1072 and 1080 cm^–1^ could be ascribed to Si–O–Si vibrations, which could belong to an intermediate unstable siliceous phase consumed by heating to form further solid solutions. These ROIs showed the same trend with the wavenumber increasing with hafnium content. Consequently, the intermediate phases could already contain zircon and hafnium. At the lower end of the hydrothermal spectra minima at approximately 448 cm^–1^ were observed and could be assigned as internal bending ν4 *E*_u_. However, only the compositions 7:1, 3:5, 1:7, and 0:1 could be evaluated and showed similar wavenumbers increase with the hafnium content. The evaluation of the minima at approximately 1400 and 1335 cm^–1^ made the presence of organic matter very likely. These ROIs are typical for modes of C–H. Furthermore, an evaluation of the wavenumbers did not exhibit a distinct dependence on the hafnium content. However, a specific organic compound could not be identified. The spectra of hydrothermal synthesis feature broad and intense modes H_2_O at approximately 1635 and 3435 cm^–1^ which diminished for the flux grown series. The modes between 742 and 745 cm^–1^ could be assigned to monoclinic ZrO_2_ (cf. refs (69,15)) and solutions with hafnium, which were also formed during hydrothermal synthesis.^70^

**Figure 11:**
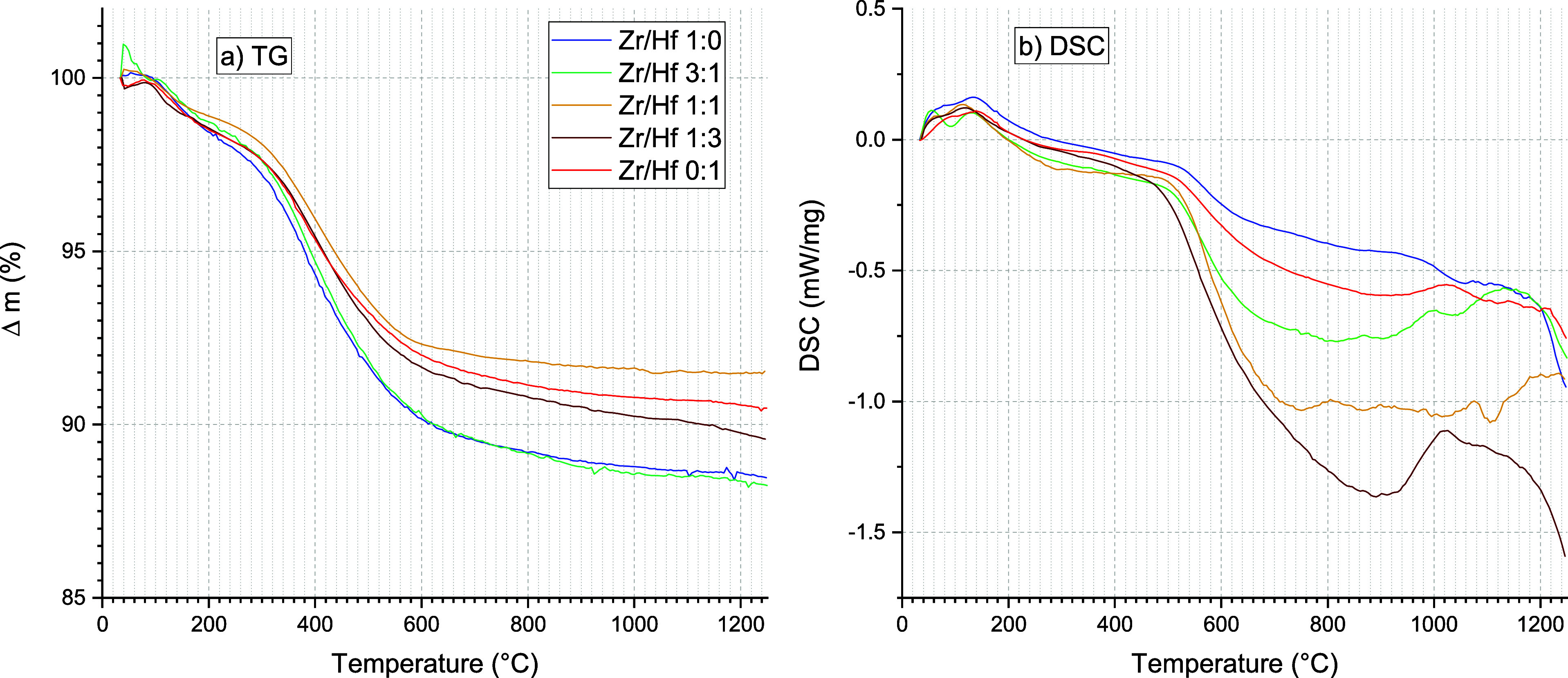
(a) TG analysis and (b) DSC analysis of the hydrothermal reaction products of the zircon-hafnon solid solution series.

The dependencies of mode shifts on the hafnium content in the hydrothermal series, shown in Figure 10 (blue lines and symbols), were consistent with results observed for the minima of the flux-grown series (red lines and symbols). With the increase of the hafnium content, the wavenumbers visually increased linearly as well. From the spectroscopic and XRD findings the hydrothermally synthesized ZHSS constitutes a complete solid solutions series without miscibility gaps because the results indicated a constant and gradual change of respective properties. Therefore, only the compositions 1:0, 3:1, 1:1, 1:3, and 0:1 were characterized by additional thermal analysis. Figure 11 shows the results of the TG (a) and the DSC measurement (b) of these samples.

### 3.5 Simultaneous TG/DSC/MS

The weight loss at 1250 °C of all samples of the solid solutions ranged from approximately 8 to 12%, but a specific dependence on the hafnium content was not obvious. Nevertheless, all investigated samples showed a similar behavior considering the weight loss. Three steps could be observed: between room temperature to approximately 275 °C, between 275 °C approximately 600 °C, and between 600 and 1250 °C.

The first two steps could be attributed to the loss of water and decomposition of organic residues. The last step contributes to approximately 2 wt % weight loss, which seemed not to be finished even at 1250 °C. In this interval, ZHSS could lose structural water during crystallite growth because exothermic signals were observed beyond 600 °C. The DSC measurement curves exhibited a wide range of scatter, but the basic course of the detected heat flow is comparable, i.e. all DSC graphs feature more or less an exothermic minimum and exhibited distinct variations in terms of broadness of the temperature range–approximately between 700 and 1100 °C: Minima (1:3 brown, 0:1 red) ≈ 900 °C; Minima (3:1 green, 1:1 dark yellow ≈800 °C); the minimum for 1:0 (blue) is not clearly observed. Beyond these minima, the DSC curves showed the tendency for further exothermic reactions, which could be explained by crystallite growth during the heat treatment, because by comparing the peak broadening in the recorded diffractograms the reflections became smaller (cf. Figures 3 and 4).

**Figure 12:**
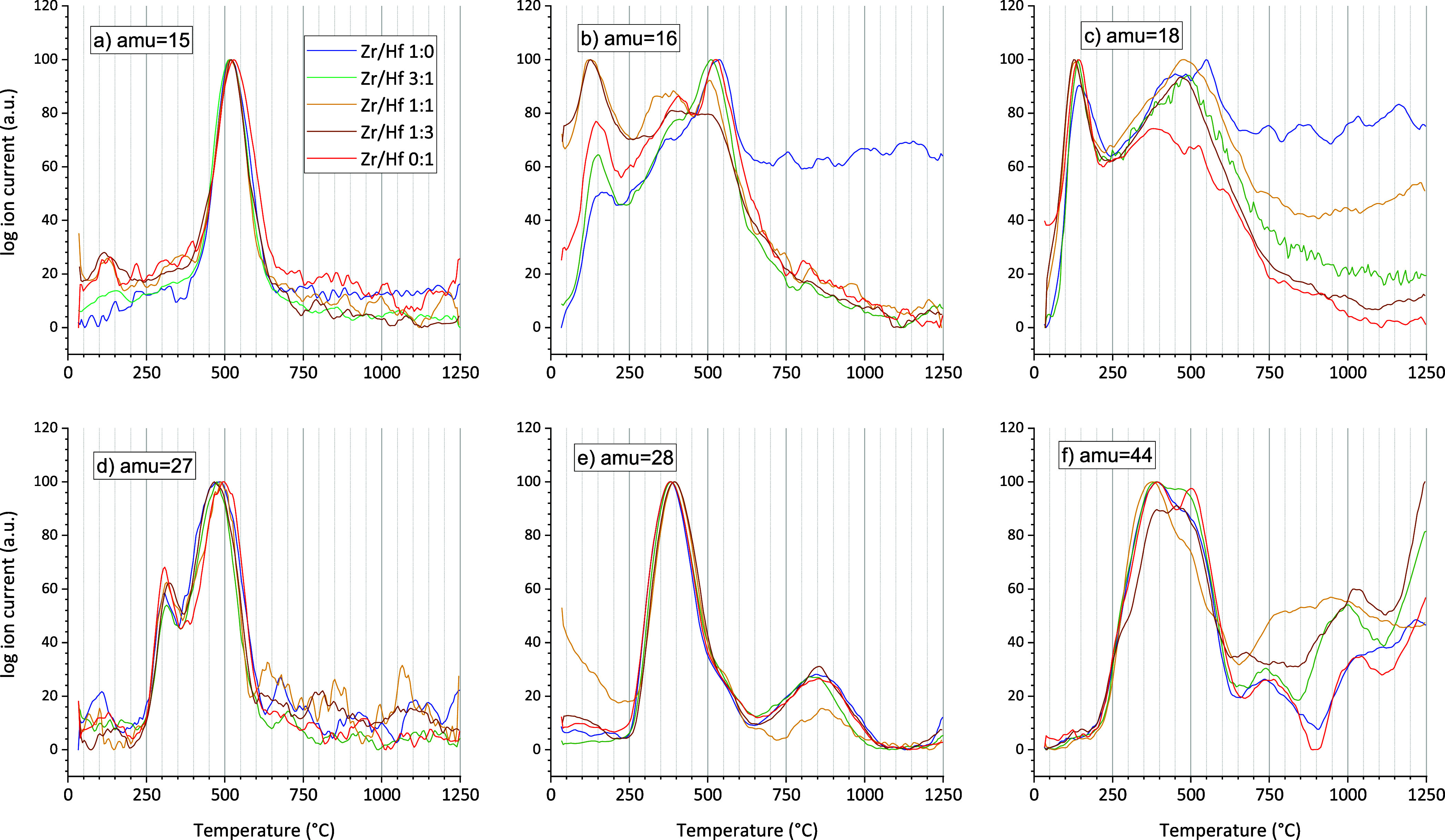
Selected normalized mass spectra being recorded simultaneously during the TG analysis of the hydrothermal reaction products of the zircon-hafnon solid solution series. (a) amu = 15 KF: CH_3_^+^; PM: C_*x*_H_*y*_, (b) amu = 16 KF: O^+^, CH_4_^+^; PM: O_2_, H_2_O, CH_4_ (c) amu = 18 KF: H_2_O^+^; PM: H_2_O (d) amu = 27 KF: C_2_H_3_^+^; PM: C_*x*_H_*y*_ (e) amu = 28 KF: C_2_H_4_^+^, CO^+^; PM: C_*x*_H_*y*_, CO, CO_2_, (f) amu = 44 KF: C_3_H_8_^+^, CO_2_^+^ and C_2_H_4_OH^+^; PM: CO_2_, C_2_H_5_OH (alcohol), C_3_H_8_.

Parallel to the TG/DSC measurement, mass spectra (Figure 12) of the volatile components also were recorded. In Figure 12, six different atom mass units (amu) are shown. The respective graphs for the ion current were normalized for comparison.

Figure 12a shows the amu = 15 which represents the release of CH_3_^+^. The parent molecule (PM) has a common composition of C_*x*_H_*y*_, but it denotes a pure organic compound because it shows a very sharp signal ranging from approximately 350 to 700 °C with a maximum at approximately 500 °C. Figure 12b) shows the amu = 16, which refers to the Key fragments (KF) O^+^ and CH_4_^+^. Possible PM could be O_2_, H_2_O, and CH_4_ but O_2_ could be present only in trace amounts because the apparatus was constantly purged with argon. In the range of room temperature to approximately 200 °C a maximum could be observed followed by a minimum at 250 °C. From 250 °C to approximately 700 °C a maximum at approximately 500 °C could be observed for all solid solutions. This observation could be explained by the release of water because amu = 18 (Figure 12c) represents the PM H_2_O exclusively and exhibited a similar behavior of the ion currents. The release of water was visually finished in a range beginning at approximately 750 °C (blue curve 1:0) to approximately 1000 °C.

Figure 12d represents amu = 27 (KF = C_2_H_3_^+^, PM = C_*x*_H_*y*_) and shows that the release of organic compounds is comparable for all investigated samples of the solid solution series. The release exhibited two stages with maxima at approximately 300 and 500 °C. The full range spans from approximately 250 to 650 °C. With further increase of the temperature, the ion current reached the start level and remained constant until the end of the measurements.

Figure 12e shows the amu = 28, which represents PM = CO, CO_2_, and C_*x*_H_*y*_. All samples show more or less the same ion currents. A first maximum could be observed at approximately 375 °C. The decrease to the minimum at approximately 625 °C featured a shoulder from 500 °C to approximately 625 °C. In the range from 625 to 1000 °C, a maximum at approximately 850 °C could be observed for all curves. The first maximum could possibly to be attributed the evaporation of organic compounds which decompose into CO and CO_2_.

The amu 44 (Figure 12f) specifies the gaseous release and composition of parent molecules like C_2_H_5_OH (ethanol), C_3_H_8_ and CO_2_. The detection of the ion current refers to KF = CO_2_^+^, C_3_H_8_^+^ and C_2_H_4_OH^+^. Based on the starting materials (TEOS and ethanol) and the IR investigation of the hydrothermal reaction products of the solid solution series, this interpretation of amu 44 is reasonable. An explanation for the detection of CO_2_ is likely due to the decomposition of organic compounds. Such an interpretation is also valid by considering the amu 28 (Figure 12d), which indicates CO, CO_2_, and C_*x*_H_*y*_ as parent molecules.

A first peak (Figure 12 f) could be observed at approximately 380 °C. For all samples, a shoulder/minimum and a second peak could be seen at approximately 400 to 500 °C. With respect to amu 44, in the range from 630 to 1100 °C further release of carbon compounds could be observed, i.e. decomposition into CO_2_. In the interval from approximately 630 to 1100 °C, similarities between all samples are more obvious than for sample 1:1 (orange). With a further increase in the temperature, compounds were released until 1250 °C.

To summarize the MS analysis, all samples show more or less the same behavior with the release of H_2_O and they all indicate the presence of ethanol and/or alkane compounds, which decompose to CO and CO_2_ during heating. A dependence on the hafnium content is not seen.

## 4 Discussion

### 4.1 Optical Microscopy, SEM

The morphologies of the two synthesized zircon–hafnon solid solution series depend strongly on the method applied, i.e. whether the reaction products were produced via a flux- or hydrothermal-based route. Idiomorphic and transparent crystals could be harvested and characterized nicely by optical means because this method resulted in crystals that were large enough (millimeter range) to observe with well-grown, distinct crystal faces (cf. Figure S13). However, the flux approach also yielded small crystals suitable for single crystal analysis without further preparation. In contrast, the reaction products of the hydrothermal synthesis did not feature any specific morphology, which is indicative of zircon crystals by application of high-resolution SEM analysis (cf. Figure 1).

This finding corresponds well with the XRD analysis of the hydrothermal reaction products, which feature very broad reflections in the respective diffractograms and indicate a crystallite size on the nanometre scale (cf. Supporting Information: Table S11b). A dependence of the morphology on the hafnium content seemed not to be obvious neither for the hydrothermal synthesis nor for the flux-grown samples of the zircon-hafnon solid solution series. For hydrothermal synthesis, the semiquantitative EDS analysis (cf. Figure 1f) also featured carbon and oxygen signals, which do not exhibit a constant ratio. This observation could be explained by the presence of unevenly distributed organic matter, which itself could be possibly ascribed to different residues of the organic starting materials (TEOS and ethanol) or to reaction products thereof, which have been formed during the hydrothermal synthesis of the solid solution series. Their presence is also evident by comparing the IR spectra of crushed single crystals and hydrothermal reaction products (cf. Figure 9, Table S28) and by evaluation of the MS analysis (cf. Figures 11 and 12).

### 4.2 X-ray Diffraction

In this study, structural gradual and constant changes could be observed, which depended on the hafnium content. A detailed view also revealed effects, that could be attributed to the synthesis route, i.e. whether the reaction products were obtained via hydrothermal or flux-grown synthesis. For the latter method, the *a*/*c* parameter, the *c*/*a* ratio, and the unit cell volume decreased clearly with the hafnium content. However, the c-parameter exhibited a stronger scatter (Figure 5c, Table S10). The single crystal data analysis shows that the lattice parameter determination exhibits more scatter than PXRD due to statistical reasons. This circumstance is clearly visually traceable by the red curves and symbols representing the PXRD analysis of the crushed single crystals in Figure 5 – cf. Table 2 as well. All fits have better *R*^2^ values (cf. Supporting Information: Table S10) than the respective data for the single crystal data (black lines and symbols in Figure 5). In addition to the statistical improvement, the crushing procedure leads to a homogenization of the composition of the intermediate single crystals (3:1, 1:1, 1:3), which could feature zoned structures (cf. Figure 8, Tables S2–S7) as observed for the 3:1 and 1:1 single crystal composition, which exhibit values with significant deviations from the expected composition (cf. Table 1). The analysis XRD (for the crushed ZHSS, Figure S12) revealed a nonlinear behavior and confirmed the findings which have been described by Cota et al.^59^ who also observed a negative deviation from linear behavior for the cell parameters. IR analysis of internal asymmetric bending modes of ν4 A2u for hydrothermal and crushed single crystals series of ZHSS (Tables 7 and 8) indicated such a correlation likewise and supported hence that XRD observation.

The lattice parameters of the hydrothermally produced material, heated in the TG measurement process (green lines and symbols in Figure 5), behave similarly to the crushed single crystals, including the a and c parameters, the *c*/*a* ratio, and the unit cell volume. However, the deviation from linearity was more evident (cf. *R*^2^ values Table S10) possibly due to some heterogeneities because the quantitative phase analysis (QPA), given in Table 5, showed that the second crystalline phase, solid solutions of Zr_1–*x*_Hf_*x*_O_2_, exhibited different amounts, which did not correlate with the increase of the hafnium content. Such irregularities independent of the Zr/Hf ratio were also observed in the thermograms and DSC analysis of these samples (Figure 11).

All investigated samples of the solid solution series featured a comparable thermal behavior; however, dependence on the hafnium content could not be observed. With respect to the hydrothermally synthesized samples, the four parameters *a*, *c*, *c*/*a*, and unit cell volume showed a clear dependence on the incorporated hafnium (cf. Figure 5 blue lines and symbols). The *c* parameter and especially the *c*/*a* ratio exhibited exceptional behavior: The *c* parameter of the hydrothermal synthesis featured the steepest decline of the linear fit with the increase of the hafnium amount compared to the single crystal and hydrothermally synthesized samples, measured (i.e., heat-treated) by TG. This result impacts the slope of the fit for the *c*/*a* ratio, which was exclusively negative whereas all other XRD analyses featured a positive slope. The *c*/*a* ratio became larger with the hafnium amount and the unit cell elongated relatively in the *c* axis direction, also in good accordance with the literature findings. Ramakrishnan et al.^54^ and Cota et al.^59^ observed this behavior for their investigated zircon-hafnon solid solution series, which were also synthesized at very high temperatures (Ramakrishnan: 1450 °C, Cota: 1550 °C (Table S9, Figure 5c) demonstrating that with an increase of temperature structural processes occurred that lead to a twist of the slope of the *c*/*a* ratio.

In comparison with the previous HfSiO_4_ study,^52^ many similarities considering peak broadening, lattice parameters could be observed, although different precursors were applied for synthesis. The unusual behavior of the *c*/*a* ratio, which was observed for the hydrothermal zircon-hafnon series, could be anticipated in the study of ref (52) as well (cf. Table S9, Tables 1–4, and Figure 5). The *c*/*a* ratio of hafnon synthesized at 150 and 200 °C, respectively, equals or was even smaller than 0.9 whereas the TG sample (1000 °C) featured a value of 0.90825–so within 1%.

Furthermore, the study showed small CS for the low-temperature hydrothermal synthesis, in part determined by the dependence of the Miller indices. The derived CS values were comparable with the results of this study. However, the CS_220_ was approximately 6 times larger. *R*-values of the Rietveld refinement considering diffractograms with anisotropic peak broadening were not reported. Estevenon et al.^52^ also observed a maximum of lattice constants for hafnon with anisotropic peak broadening. Furthermore, the thermal behavior showed a similar weight loss of approximately 6 to 12.5 wt % depending on the synthesis temperature. These values are in good agreement with the study here, which featured approximately 10.1 wt % weight loss at 1000 °C for hafnon (cf. Figure 11a). The complete solid solutions weight loss ranged from 8.4 to 11.5 wt %. The 0:1 sample (HfSiO_4_) which was subjected to TG also had *c*/*a* ratios which were typical for hafnon. Furthermore, *c*/*a* ratios of the hydrothermal synthesis, i.e. for lower temperatures were also in good agreement with this study (cf. Supporting Information: Table S9). However, contrary to our study Estevenon et al.^52^ focused on the hydrothermal synthesis of pure hafnon and was successful by applying a multiparametric approach considering the concentration of precursors, pH, and temperature. Our study focused on the synthesis of the ZHSS by a TEOS-based approach, without primarily considering parameters such as pH, concentration of precursors, and temperature. Hence, IR, XRD of TG samples, and MS revealed the presence of byproducts.

At present, one could only speculate to which extent these processes depend on the loss of OH-groups in Zr_1–*x*_Hf_*x*_(SiO_4_)_1–*y*_(OH)_4*y*_ and/or on crystal growth, which could have an impact on the real structure as well. The presence of such hydroxyl groups could not be ruled out because the unit cell volume (cf. Figure 5d) for hydrothermal synthesis is larger than those determined for samples synthesized at high temperatures or had experienced a massive heat treatment (e.g., TG analysis). An analogue behavior could be observed for garnets. The lattice parameters of grossular Ca_3_Al_2_(SiO_4_)_3_ (*a* = 11.872(1) Å, cf. ref (71)) and katoite Ca_3_Al_2_((OH)_4_)_3_ (*a* = 12.565(3) Å, cf. ref (72)) are different with the parameter for katoite greater than that for grossular. Yet, in a study by Caruba et al.^73^ in which zircon was also hydrothermally synthesized by additional usage of fluorine, it was pointed out that lattice parameters became smaller due to the fluorine which will also replace with OH^–^ the SiO_4_ tetrahedron in Zr_1–*x*_Hf_*x*_(SiO_4_)_1–*y*_(OH/F)_4*y*_. Here, the hydrothermal zircon-hafnon series was synthesized without fluorine so the structural situation could be different. According to Julg and Ozias,^74^ a replacement of the SiO_4_ entity with 4 OH^–^ anions in zircon is also possible due to crystal field stabilization. Lei et al.^45^ used TEOS and NaF as mineralizers for the hydrothermal synthesis of zircon. They explained the peak shifts in comparison with “normal zircon” due to the impact of OH^–^ and F^–^ ions. The role of organic residues of the TEOS precursor was not discussed. According to TG and XRD analysis of Estevenon et al.,^52^ the shrinkage of the lattice parameters could be observed due to the release of water being present in the hafnon lattice.

**Figure 13:**
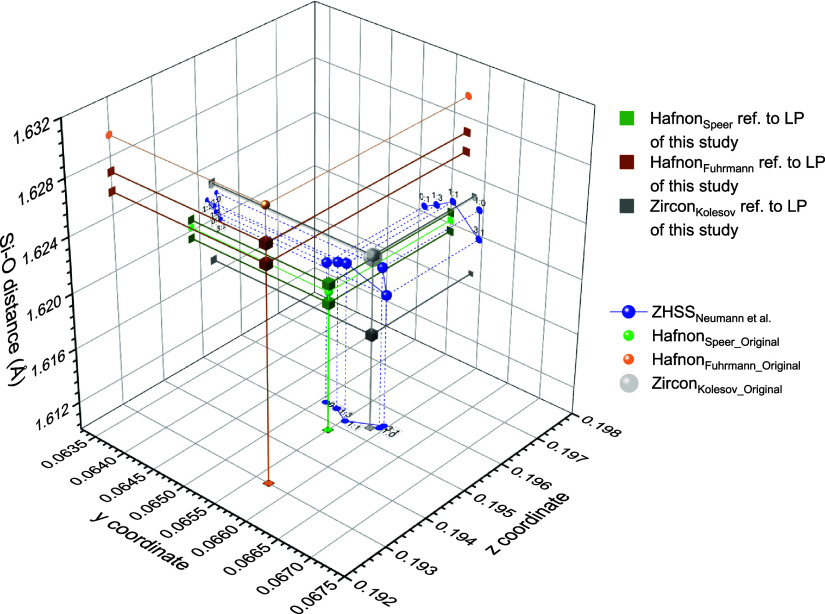
Dependence of the Si–O distance from the composition of the zircon hafnon solid solution and *y*/*z* atomic positions.

A further issue of the XRD analysis was the determination of the atomic position for y and z coordinates for oxygen in the Zr_1–*x*_Hf_*x*_SiO_4_ solid solutions. Kolesov et al.^29^ also analyzed synthetic zircon and very good agreement has been achieved - *y* = 0.06594 and *z* = 0.19543. To our best knowledge currently no structural data for the intermediate solid solutions were investigated and reported to the structural ICSD database. Consequently, the authors suggest the atomic coordinates which were determined via single crystal analysis (cf. Table 1). These *y*-*z* atomic positions (*x* and *y* axes) are shown in Figure 13 with the dependence of Si–O distance (*z*-axis) in the SiO_4_ tetrahedron.

Lattice constants were determined by Rietveld refinements of the crushed single crystals of the zircon–hafnon solid solution series because the crushed single crystal analysis overcomes the inherent problem of zoning effects in the solid solutions. Using the atomic positions obtained by SHELXL and the lattice constants obtained by Rietveld refinements for the calculation of the Si–O distance leads to unique solutions in the 3D coordinate system for *y*, *z*, and Si–O distance, as shown in Figure 13.

The blue spheres in Figure 13 indicate the measured data of the ZHSS. With the increase of the hafnium amount, the Si–O distance decreases; however, the 3:1 composition could be an outlier.

Due to the observed linear dependences of spectroscopic data on the increase of the hafnium content (cf. Figures 8/10), a downward path (interconnected big blue spheres) featuring a small inclination with respect to the *z*-axis (decrease of the Si–O bond length) can be taken.

Figure 13 features also data for zircon (ref (29) – green symbols) and hafnon (ref (39) – brown symbols, ref (38) – gray symbols). Each data set was plotted with reference to the lattice parameters of the single crystal (incl. crushed samples– lower and upper cube symbols) study and the original study (sphere symbols). Good accordance was achieved for the data of Kolesov (zircon), and Speer (hafnon). However, the respective data of Fuhrmann (hafnon) feature more distant values considering Si–O-distance and *z*-coordinate.

**Figure 14:**
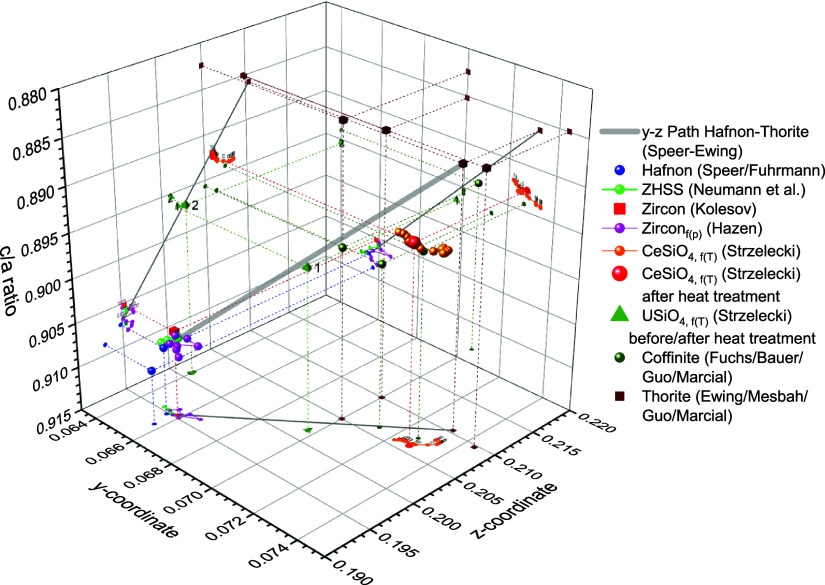
Selected reference data of *y*/*z* atomic positions of isostructural orthosilicates, hafnon, zircon, stetindite, coffinite, and thorite, dependent on the *c*/*a* ratio. Numbers above the zircon series by Hazen^28^ indicate the increase from ambient to high pressures (*p*_max_ = 48.1 kbar). Numbers above orange spheres of the stetindite (CeSiO_4_) series by Strzelecki^75^ indicate the increase from ambient to high temperatures (*T*_max_ = 865 °C); big red sphere indicates stetindite after the heat treatment at room temperature. Green tetrahedra indicate coffinite before (1) and after (2) heat treatment by Strzelecki^75^; for details in Figure 14 cf. Supporting Information file: S_Fig_14.opju.

Speer and Cooper^38^ discussed a linear correlation between the increase of *y* and *z* coordinates and lattice parameters of isostructural orthosilicates like zircon and thorite. Hence, the *y* and *z* values as shown in Figure 14 increase in the following order: Hafnon–zircon–thorite. For completeness, additional selected references of stetindite (cf., ref (75) coffinite, and thorite (cf. refs (76−79)) were also plotted and basically follow this idealized linear trend (gray line as a guide to the eye) and exhibit deviations from this path from hafnon to thorite (ThSiO_4_) synthesized by Taylor and Ewing^80^. Consequently, the approach of Speer and Cooper^38^ is not suitable to classify these isostructural orthosilicates. Recent research considers thermodynamics, i.e. Δ*H*_f,Ox_ in dependence of ionic radii,^81^ because some orthosilicates are metastable and not easy to synthesize in contrast to hafnon, zircon, and thorite and need for purification a post treatment.^82^

Consequently, the preparation and analyses of more intermediate compositions for single crystals, e.g., 7:1, 5:3, 3:5, 1:7, could help sharpen the resolution of atomic coordinates for the ZHSS.

### 4.3 Spectroscopy: Raman, IR, and μXRF

Due to the good quality of samples, achieved via the flux growth (cf. Figure S13), single crystals were selected and measured with Raman spectroscopy. Furthermore, this analysis was complemented by μXRF because both methods are primarily sensitive surface probes. Fitting procedures, which consider the surface composition of these single crystals as well (red lines and symbols in Figure 8), were compared to the linear fits, which consider the composition of the solid solutions and reflect the ZrO_2_/HfO_2_ ratio based on the weighted amounts (black lines and symbols). This bivalent method was applied for 10 out of 12 modes, which had been observed. Hence, *E*_g_(II) and *B*_1g_(II) were not considered, because these bands were only observed for the end-members of the zircon-hafnon series (cf. Figure 8a,b). Furthermore, the number of measurements (one on the zircon crystal, three on the hafnon crystal) was significantly less for the end-members than for the mixed compositions 3:1, 1:1, and 1:3. For these latter compositions two crystals were chosen and each measured five times. Figure 8 shows also the differentiation for the two 1:1 crystals (S1: blue symbols, S2: green symbols), because the μXRF analysis of these two samples revealed huge differences for the same nominal 1:1 composition (S1 = 0.396, S2 = 0.565). Similar observations were also reported in a previous study.^58^ Furthermore, this μXRF finding could hence explain the differences in the spectra of the two analyzed crystals (cf. Figure S13c_1/2) with the same nominal 1:1 ratio; two broad bands at approximately 201 and 216 cm^–1^ merge into one band at approximately 213 cm^–1^ (cf. Figure 7a) with the increase of hafnium. Yet, such an evident deviation was not observed for the pair of crystals of the mixed compositions 3:1 and 1:3 (cf. Supporting Information Tables S2, S3, S6, S7). The linear fitting procedure showed by evaluation of the *R*^2^ values (cf. Supporting Information: Raman – Linear Mode Fit) that six (*B*_1g_(I), *A*_1g_(ν2), *B*_1g_(ν4), *E*_g_(ν3), *A*_1g_(ν1), and *B*_1g_(ν3)) out of ten fits resulted in higher *R*^2^ values for the μXRF samples. Four *R*^2^ values (*E*_g_(III), *B*_2g_(ν2), *E*_g_(I), and *E*_g_(ν4)) favored the weighted Zr/Hf ratio. However, considering the μXRF findings of the nominal 1:1 composition (cf. blue and green symbols in Figure 9), the Zr/Hf ratio determined by μXRF should be considered as more reliable. Therefore, these results support the nonequilibrated growth of this solid solution series.

In comparison, the observed modes in this study were in accordance with experimental Raman data of previous studies. Nicola and Rutt^55^ investigated the end-members zircon and hafnon. The zircon data were consistent, however, *E*_g_(ν4) and *B*_1g_(ν4) were not observed in a previous study.^55^ Vice versa, the *B*_2g_(ν2) was not observed in the present study. For hafnon, *B*_2g_(ν2) was observed in this study but not in the study of Nicola and Rutt.^55^ Such was also true for *E*_g_(ν3), *E*_g_(ν4), and *B*_1g_(ν4) that were not observed in both studies. The hafnon modes, which were observed in both studies, showed slightly increased deviations of approximately 1–2 cm^–1^. In a more recent study by Grüneberger et al.,^58^ experimental Raman data of the zircon-hafnon solid solution series were reported. They synthesized and analyzed 7 compositions for the ZHSS whereas in this study 5 mixtures were probed. Good accordance with the observed modes in comparison with Grüneberger et al.^58^ was obtained. *B*_2g_(ν2) was not observed and both studies did not show the presence of the *E*_g_(ν3) mode. In this study, three modes between ∼147–166 cm^–1^ were observed for hafnon. At approximately 156 cm^–1^, the observed mode was very strong and at the left and right peak bases, the presence of further modes could not be ruled out. With respect to the *E*_g_(II), *B*_1g_(II), and *E*_g_(III) hafnon modes,^58^ suggested - based on theoretical calculations–a revision of the *E*_g_(II) mode for hafnon, reported by Nicola and Rutt^55^ and Hoskin and Rodgers.^57^ The assignment of these three modes for the end-member hafnon is ambiguous because Syme et al.^56^ who investigated the Raman spectra of zircon and thorite (ThSiO_4_), argued for a different assignment of the *E*_g_(II), *B*_1g_(II), and *E*_g_(III) modes of the hafnon end-member. We followed the assignment suggested by Syme et al.^56^

With respect to the intermediate compositions of this study and that of Grüneberger et al.^58^ study, both studies experienced the same challenges in observing distinct modes, which exhibited a clear dependence on the hafnium content based on the *E*_g_(II), *B*_1g_(II), and *E*_g_(III) mode shifts from zircon to hafnon as shown for the two samples, that feature the 1:1 composition (cf. Figures 6 and 7a). Figures 6 and 7a, the intermediate ROI for the mixed compositions 3:1, 1:1, and 1:3 is very broad, i.e. “more-hump” like modes could be observed. To establish a sound fit of the full spectra, additional supporting modes (SM) were introduced for the fit of these broad bands (cf. Table 6). However, these broad bands were not assigned to any of the *E*_g_(II), *B*_1g_(II), and *E*_g_(III) modes. At the current state of knowledge such assignments are still a guess and more supporting data are lacking to explain the observed phenomena. Further hafnon modes observed by^58^ were in excellent accordance with the observations of this study (cf. Table 6). However, the *E*_g_(ν4) and *B*_1g_(ν4) bands were not observed in their study. The dependences on the hafnium amount in solid solutions feature the same trends as reported in Grüneberger et al.^58^ and hence confirm and consolidate the findings.

The IR characterization of the zircon–hafnon solution series revealed some significant differences between the hydrothermally synthesized and flux-grown reaction products. Both series feature modes, at approximately 3430 and 1640 cm^–1^ which could be ascribed to the presence of water. For the flux-grown samples, these observed modes could also be ascribed to water, present in the KBr matrix, used for the IR pellets (cf. Supporting Information Figure S14). However, these modes were much larger in terms of transmission for the hydrothermal series (cf. Figure 9). Several reasons are possible for this observation. The presence of water, which could be absorbed and/or incorporated in the structure, and due to the nanosized dimensions of crystallites, there is a much more specific surface area on which water molecules could be adsorbed. In addition, it is likely that the hydrothermal reaction products also feature organic residues or organic reaction products of the starting materials, which could exhibit functional OH groups. Modes at approximately 1395 and 1335 cm^–1^ could be ascribed to organic molecules, which feature such properties according to the EPA NIST database. Furthermore, a clear specific hafnium dependence of the wavenumbers of these ROIs was not obvious (cf. Tables S24–S28). However, at the current state of knowledge, an exact composition of these assumed organic remnants could not be given, because the database is also limited and does not feature metal–organic compounds, which could have been formed as well. The mode at approximately 1075 cm^–1^ (cf. Table 8) belongs most likely to Si–O–Si vibrations of silicious precursors, which exhibited an impact on the hafnium content because these modes were not present in comparison with IR spectra of the flux-grown series (cf. Figure 9). The same is also valid for the ROI at approximately 743 cm^–1^ which could be attributed to vibrations of the monoclinic Zr_1–*x*_Hf_*x*_O_2_ solid solutions (cf. refs (69,15)). Pitcher et al.^70^ showed that monoclinic ZrO_2_ could be stable alongside tetragonal ZrO_2_ at low temperatures. Consequently, it is highly likely that the SiO_2_ and Zr_1–*x*_/Hf_*x*_O_2_ react to Zr_1–*x*_/Hf_*x*_SiO_4_ upon heating because these modes were not observed for the flux-grown solid solution series. However, the modes at approximately 900 cm^–1^ (internal stretching mode) and 635 cm^–1^ (internal bending mode) seem to have analogue modes for the flux-grown samples at approximately 900 and 615 cm^–1^ (cf. Figure 10a,b). All these observed modes increase with the increase of the hafnium content. The differences between these two synthesis routes are an approximately twice broader range for the shift to higher wavenumbers for the hydrothermal samples (Δ ∼ 23 cm^–1^) compared to the crushed single crystals (Δ ∼ 11 cm^–1^). Furthermore, the lower wavenumber of the hydrothermal synthesis became even larger than the wavenumbers for the crushed single crystals of the solid solution series with an increase in the hafnium content. The linear fits of the hydrothermal and flux-grown solid solutions visually intersect at approximately *x*(Hf) = 0.55 at a wavenumber of approximately 898 cm^–1^ (cf. Figure 10a). This observation could not be explained by comparing the Si–O distance of the SiO_4_ tetrahedron (cf. Figure S10a), because this parameter always displayed larger values for the hydrothermal solid solutions. However, the determined interatomic distances between Zr/Hf–O (edge sharing Oxygen), Zr/Hf–Si (cf. Figure S10), and the c parameter (cf. Figure 5b, Tables 2/3) exhibited an analogous, but inverted behavior compared to the development of wavenumbers of hydrothermal and flux grown series of this solid solution series, i.e. the slope depending on the hafnium content inclined steeper for the hydrothermal samples (cf. Figure S10). Consequently, with increasing hafnium content the observed behavior of wavenumbers depended on the different development of Zr/Hf–Si/O bond lengths for the hydrothermal and the crushed single crystal solid solutions, respectively. However, this interpretation of the Zr/Hf- and Si–O bond length is based on the atomic positions of the single crystal refinements. Attempts to refine these positions for the hydrothermal syntheses did not exhibit plausible results, i.e. Si–O distances were too short (∼1.55 Å) and systematic dependencies of the hafnium content were barely evident. One possible explanation is the content of amorphous fractions which could have an impact on the intensities of broad peaks of the hydrothermally synthesized solid solutions and so impose some limitations on the refinement of *y* and *z* coordinates of samples featuring a distinct anisotropic peak broadening.

The internal bending modes, which cover a comparable range of approximately 6–8 cm^–1^, were shifted by approximately 22 cm^–1^ to higher wavenumbers for the hydrothermal solid solutions (Hydrothermal range: ∼632 to 639 cm^–1^, flux grown range: ∼610 to 617 cm^–1^). Estevenon et al.^52^ synthesized hafnon at low temperatures and observed a shift to 627 cm^–1^ for this mode. Caruba et al.^73^ who investigated synthetic hydrothermal zircon reported a similar shift from 615 to 635 cm^–1^ and explained this shift by the replacement of the SiO_4_ tetrahedron with (OH/F)^−^, which has a different atomic weight.

The mode at approximately 433 cm^–1^ represents the internal bending mode (ν4 *E*_u_) and was slightly shifted toward higher wavenumbers at approximately 434 cm^–1^ with an increase in the hafnium content in the flux-grown series. Woodhead et al.^11^ assigned this mode as the external rotational vibration of the SiO_4_^–^ tetrahedron. For the hydrothermal series, this mode seemed to shift to higher wavenumbers at approximately 446 cm^–1^. However, this ROI could not be evaluated fully for the hydrothermal series. Only four modes (7:1, 3:5, 1:7, 0:1–ranging approximately from 446 to 450 cm^–1^) could be evaluated (cf. Table 8, Figure 9) and showed the same tendency as the flux-grown series, i.e., the wavenumbers with the increasing hafnium content. However, the range of the hydrothermal series seemed to be approximately 4 times larger for the internal bending mode (ν4 *E*_u_) irrespective of the uncertainties.

For the flux-grown series, an analogous behavior was seen for the internal stretching mode at 970 cm^–1^, which increased to approximately 984 cm^–1^ with an increase in the hafnium content. However, the modes observed at 451 cm^–1^ behaved in the opposite manner, i.e., the wavenumber decreased to approximately 443 cm^–1^ and was dampened significantly with the increase of hafnium (178.5 g/mol), which exhibits nearly twice the atomic mass of zirconium (91.2 g/mol). The Zr/Hf–O bond length decreases also with Hf content (cf. Table 1, Figure S15). Consequently, this shift is due to other external vibrational effects, which have still to be evaluated. For the zircon end-member, Woodhead assigned this mode also as internal bending mode (ν4 *E*_u_). We did not adopt the assignments for these modes proposed by Woodhead et al.^11^ Tartaj et al.^46^ who investigated ZrSiO_4_ spherical particles, produced by hydrolysis of aerosols from mixtures of TEOS and Zr-n-propoxide observed a mode close to our observations at 455 cm^–1^ and attributed it to Zr–O vibrations.

Furthermore, the characteristic of this mode changed from a minimum to a shoulder with an increase of the hafnium amount. The IR spectra also featured two further modes, which increased with the hafnium content. However, the weak minimum at approximately 1800 cm^–1^ was only observed in this study (cf. Table 7) and was not reported elsewhere. Modes ranging from approximately 1790 to 1810 cm^–1^ could represent overtones of the internal stretching modes observed at approximately 900 cm^–1^ because the range of modes depending on the hafnium content has also been doubled from 10 cm^–1^ to approximately 20 cm^–1^. Furthermore, the intensity of this mode's ROI weakened with an increase in the hafnium content.

### 4.4 Simultaneous TG/DSC/MS

Thermogravimetric analysis, combined with simultaneous DSC (cf. Figure 11) and MS (cf. Figure 12), showed that the selected compositions of hydrothermally synthesized samples of the zircon–hafnon solid solutions series exhibited more or less a similar behavior upon heating to 1250 °C. However, irrespective of the method a distinct trend depending on the hafnium content was not observed. Considering the loss of weight, three steps, from ambient to approximately 275 °C, from approximately 275 to 600 °C, and from 600 to 1250 °C, were observed for all samples. The first step could be attributed to the loss of water, which could be adsorbed on the surface. Furthermore, this process could also be accompanied in part by the loss of organic matter. However, according to the ion currents, organic compounds seemed to be released at higher temperatures.

For all samples, the weight loss of this type of water was about 2 to 3%. However, Figure 12c) shows the mass spectra for H_2_O, with a second very broad peak from approximately 300 to 700 °C, with a maximum at approximately 500 °C. In this ROI a further release of water was observed ascribed to the decomposition of organic compounds. Furthermore, upon heating and crystallite growth hydroxyl groups began to leave Zr(SiO_4_)_1–*x*_(OH)_4*x*_, because zircon can contain water (cf. e.g., refs (83−85)). This process may have not come to an end at 1250 °C, because the weight loss graphs were still slightly declining (cf. Figure 12a). Hence, higher-end temperatures and reduced heating and cooling rates for combined TG/DSC/MS analysis could help explore in-depth the role of structural water in hydrothermally synthesized zircon. In-situ HT-XRD is also a useful and promising tool to explore the thermal properties of such silicates, e.g., stetindite CeSiO_4_ and coffinite USiO_4_ (cf. Strzelecki et al.^75^), belonging to the same space group *(I4*_*1*_*amd)* as zircon and hafnon.

For the respective IR spectra shown in Figure 9, modes at approximately 1400 and 1335 cm^–1^ support the assumption for the presence of organic residues. Tartaj et al.,^46^ who also used TEOS, observed residual organic matter. By comparing the TG analysis with the DSC (cf. Figure 11) most of the heat flux was detected in a range from approximately 500 to 1000 °C, i.e. in this range the loss of weight was nearly complete. Consequently, exothermic reactions occurred, which could be ascribed to reactions between unreacted SiO_2_ and Zr_1–*x*_Hf_*x*_O_2_ compounds to form Zr_1–*x*_Hf_*x*_SiO_4_. At temperatures beyond 1000 °C a tendency for more exothermic heat release was observed (cf. Figure 11b), ascribed to crystal growth, because the peak shapes in the respective diffractograms featured increased sharpness (cf. Figures 3 and 4), i.e., 001 crystallite sizes increased (cf. Supporting Information: Table S11).

## 5 Conclusions

In this study, the zircon-hafnon solids solution series has been synthesized via two approaches. The first route was established successfully via the flux growth of single crystals at high temperatures starting at 1400 °C, the second route constitutes a hydrothermal TEOS-based route at 200 °C. Although not showing visually any byproducts in the diffractograms of the low-temperature route, the hydrothermal reaction products exhibited the presence of organic residues and other amorphous phases. The presence of carbon compounds was clearly evidenced by the application of a combined TG/DSC/MS investigation. Amorphous siliceous compounds in addition to amorphous Zr/Hf oxides were also present, as was shown by evaluation of the XRD diffractograms and IR spectra. Consequently, the introduction of peak phases (representing amorphous phases) was beneficiary for the Rietveld refinements, which revealed more clearly the dependence of the structural properties for the hydrothermal synthesis on the hafnium content. The impact of the organic residues of the observed shift of the lattice parameters has to be addressed in future studies because according to Lei et al.^45^ who also used TEOS concluded that their shifts of the lattice parameters are due OH^–^ and F^–^ ions.

Morphological investigations showed that the single crystals of the flux method were idiomorphic, whereas the hydrothermal reaction products did not reveal any typical crystallographic features. Reaction products of both approaches showed a hundred percent miscibility as was demonstrated by XRD analysis. However, depending on the synthesis method structural differences were apparent by investigating the *c*/*a* ratio, which decreased for the hydrothermal route and increased for the flux-grown series with an increase of the hafnium amount. The result is consistent with other studies that investigated the solid solution series obtained via high-temperature routes. The synthesis by a TEOS-based hydrothermal route resulted in ZHSS, however experimental parameters as reported by Estevenon et al.^52^ should be optimized to get rid of byproducts that could have effects of data evaluation.

IR spectroscopy complemented both approaches of synthesis and revealed differences, which could be related to the chosen route for synthesis. The internal bending modes were shifted to higher wavenumbers for the hydrothermal reaction products and the internal stretching modes covered a broader range of wavenumbers. For the stretching modes, IR and XRD evaluations of the hydrothermal and flux-grown series showed that phenomena of the short-range order were related to the development of interatomic distances. However, such refers to the atomic coordinates of oxygen which were determined by single crystal analysis. Attempts to refine the *y* and *z* coordinates for the hydrothermal series resulted in less consistent data. This observation could be due to additional amorphous phase content, which belongs to other phases than to the ZHSS. This could also have an impact on the intensity of the very broad reflections for the anisotropic peak broadening.

For the IR spectroscopy of the crushed single crystals, obtained via flux growth, the results were consistent with literature findings, yet weak overtones of internal stretching modes were also observed in this study. Raman spectroscopy, which was carried out only for single crystals, also confirmed the results of other studies, that had investigated the solid solution series including the uncertainties related to the *E*_g_(II), *B*_1g_(II), and *E*_g_(III) modes of mixed compositions and the hafnon end-member. Therefore, additional in-depth studies are still necessary to investigate the Raman spectra in this ROI. Furthermore, the investigated single crystals could possibly feature zoning, which has been assumed based on additional μXRF measurements, that complemented the Raman investigations. This zoning was also apparent from additional PXRD analysis of the crushed single crystals because the scatter of the lattice parameters of the ZHSS of single crystals was significantly reduced and revealed the dependences on hafnium content more precisely. For the thermal analysis of the hydrothermally obtained reaction products of the solid solution series no dependence on the hafnium content was apparent, however, the simultaneous recording of mass spectra, complementing the TG/DSC analysis, confirmed the presence of organic matter.

To sum up, for the development of pure Zr_1–*x*_Hf_*x*_SiO_4_ compounds for specific applications, the TEOS based approach still has potential for optimization.

## Supporting Information Available

**μXRF:** measured elements in atom percent units; Zr/Hf amounts are normalized to unity (=1), i.e., *x*(Zr) + *y*(Hf)= 1 with *x*,*y* = 0 to 1; yellow cross symbols indicate the position of the μXRF measurement; red circle denotes the diameter of the μXRF probe (∼20 μm); phase evolution during heat treatment of hydrothermal zircon at 500°, 1000 °C, and 1250 °C; interatomic distance between Si and O (SiO_4_ tetrahedron), Zr_1–*x*_Hf_*x*_ and O, connecting the tetrahedra via common edges in the *c*-direction, and Zr_1–*x*_Hf_*x*_ and O, connecting the tetrahedra via bridging O with Zr_1–*x*_Hf_*x*_ (ab-plane) (d) Interatomic distance between Zr_1–*x*_Hf_*x*_ and Si; lattice constants–literature comparison zircon, hafnon listed in the ICSD database 2018/2 (cf. (Zagorac et al.,^21^)) for zircon and hafnon; images of zircon-hafnon solid solution single crystals mounted on capillaries used for single crystal analysis–grid distance equals 100 μm; *R*^2^ values for *a*, *c*, *c*/*a*, and unit cell volume for Single Crystal XRD, crushed single PXRD, hydrothermal synthesis, and TG based on the linear fit in Figure 7; crystallite size of crushed single crystals of the zircon-hafnon solid solutions dependence on the Miller indices; crystallite size of hydrothermal synthesis zircon-hafnon solid solutions (HT) dependence on the Miller indices – Part 1 (Zr/Hf: 1:0–1:1); crystallite size of hydrothermal synthesis zircon-hafnon solid solutions (HT) dependence on the Miller indices – Part 2 (Zr/Hf: 3:5–0:1); crystallite size of zircon–hafnon solid solutions of hydrothermal synthesis being subjected to TG analysis (1250 °C) dependence on the Miller indices; negative deviation of the lattice constants from linear behavior of the crushed single crystals, (a) *a* parameter, (b) Unit cell volume UC, (c) *c* parameter with data points for *x*(Hf) considered for fitting, (d) *c* parameter without *x*(Hf) = 0.75, i.e., 1:3; **Raman:** images of zircon-hafnon solid solution single crystals glued on sample holders used for Raman and μXRF analyses; fitted results for the observed Raman modes of the single crystals (cf. Figure 2) of the zircon-hafnon solid solution series; linear mode fit in dependence on *x*(Hf); **IR/Raman:** comparison of blank KBr matrix (black) and KBr matrix with zircon (red: crushed single crystal); wavenumber dependencies of IR and Raman modes on Si–O and ZrHf–O bond length; and IR modes of H_2_O and organic residues (PDF)
CIF files of the single crystal analyses of the ZHSS (ZIP)
Origin File of Figure 14^14^ (S_Fig_14.opju) (ZIP)

## References

[ref1] Hanchar J. M.; Hoskin P. W. O., Eds., Zircon; Mineralogical Society of America, 2003, 53.

[ref2] Ge R.; Wilde S. A.; Nemchin A. A.; Whitehouse M. J.; Bellucci J. J.; Erickson T. M.; Frew A.; Thern R.; E R. A 4463 Ma apparent zircon age from the Jack Hills (Western Australia) resulting from ancient Pb mobilization. Geology 2018, 46, 303–306. 10.1130/G39894.1.

[ref3] Kaiser A.; Lobert M.; Telle R. Thermal stability of zircon (ZrSiO4). Journal of the European Ceramic Society 2008, 28, 2199–2211. 10.1016/j.jeurceramsoc.2007.12.040.

[ref4] Ewing R. C. Nuclear waste forms for actinides. Proc. Natl. Acad. Sci. U. S. A. 1999, 96, 3432–3439. 10.1073/pnas.96.7.3432. 10097054 PMC34285

[ref5] Meldrum A.; Zinkle S. J.; Boatner L. A.; Wu M.; Mu R.; Ueda A.; Henderson D. O.; Ewing R. C. Radiation Effects in Zircon, Hafnon, and Thorite: Implications for Pu Disposal. MRS Online Proc. Libr. 1998, 540, 395–400. 10.1557/PROC-540-395.

[ref6] Meldrum A.; Zinkle S. J.; Boatner L. A.; Ewing R. C. Heavy-ion irradiation effects in the ABO4 orthosilicates: Decomposition, amorphization, and recrystallization. Phys. Rev. B 1999, 59, 3981 10.1103/PhysRevB.59.3981.

[ref7] Burakov B. E.; Anderson E. B.; Shabalev S. I.; Strykanova E. E.; Ushakov S. V.; Trotabas M.; Blanc J. Y.; Winter P.; Duco J. The behavior of nuclear fuel in first days of the Chernobyl accident. MRS Online Proc. Libr. 1996, 465, 1297–1308. 10.1557/PROC-465-1297.

[ref8] Ewing R. C.; Murakami T. Fukushima Daiichi more than one year later. Elements 2012, 8, 181–182. 10.2113/gselements.8.3.181.

[ref9] Nasdala L.; Zhang M.; Kempe U.; Panczer G.; Gaft M.; Andrut M.; Plötze M. Spectroscopic methods applied to zircon. In Reviews in Mineralogy and Geochemistry – Zircon, Eds. Hanchar J. M.; Hoskin P. W. O.; Mineralogical Society of America, 2003, 53, 427–467.

[ref10] Titorenkova R.; Gasharova B.; Mihailova B.; Konstantinov L. Attenuated total-reflection infrared microspectroscopy of partially disordered zircon. Canadian Mineralogist 2010, 48, 1409–1421. 10.3749/canmin.48.5.1409.

[ref11] Woodhead J. A.; Rossman G. R.; Silver L. T. The metamictization of zircon: Radiation dose-dependent structural characteristics. Am. Mineral. 1991, 76, 74–82.

[ref12] Zhang M.; Salje E. K. H.; Farnan I.; Graeme-Barber A.; Daniel P.; Ewing R. C.; Clark A. M.; Leroux H. Metamictization of zircon: Raman spectroscopic study. J. Phys.: Condens. Matter 2000, 12, 1915 10.1088/0953-8984/12/8/333.

[ref13] Zhang M.; Salje E. K. H. Infrared spectroscopic analysis of zircon: Radiation damage and the metamict state. J. Phys.: Condens. Matter 2001, 13, 3057 10.1088/0953-8984/13/13/317.

[ref14] Zhang M.; Salje E. K. H.; Ewing R. C.; Daniel P.; Geisler T. Applications of near-infrared FT-Raman spectroscopy in metamict and annealed zircon: oxidation state of U ions. Phys. Chem. Miner. 2004, 31, 405–414. 10.1007/s00269-004-0399-6.

[ref15] Zhang M.; Salje E. K. H.; Wang A. H.; Li X. J.; Xie C. S.; Redfern S. A. T.; Li R. X. Vibrational spectroscopy of fast-quenched ZrSiO4 melts produced by laser treatments: local structures and decomposed phases. J. Phys.: Condens. Matter 2005, 17, 6363 10.1088/0953-8984/17/41/007.

[ref16] Zhang M.; Boatner L. A.; Salje E. K. H.; Honda S. I.; Ewing R. C. Pb+ irradiation of synthetic zircon (ZrSiO4): Infrared spectroscopic investigation. Am. Mineral. 2008, 93, 1418–1423. 10.2138/am.2008.2733.

[ref17] Shannon R. D. Revised effective ionic radii and systematic studies of interatomic distances in halides and chalcogenides. Acta crystallographica section A: crystal physics, diffraction, theoretical and general crystallography 1976, 32, 751–767. 10.1107/S0567739476001551.

[ref18] Neves J. C.; Nunes J. L.; Sahama T. G. High hafnium members of the zircon-hafnon series from the granite pegmatites of Zambézia. Mozambique. Contributions to Mineralogy and Petrology 1974, 48, 73–80. 10.1007/BF00399111.

[ref19] Wilk G. D.; Wallace R. M.; Anthony J. High-κ gate dielectrics: Current status and materials properties considerations. Journal of applied physics 2001, 89, 5243–5275. 10.1063/1.1361065.

[ref20] Burakov B. E.; Anderson E. B.; Zamoryanskay M. V.; Yagovkina M. A.; Strykanova E. E.; Nikolaeva E. V. Synthesis and study of 239Pu-doped ceramics based on zircon, (Zr, Pu) SiO4, and hafnon, (Hf, Pu) SiO4. MRS Online Proc. Libr. 2000, 663, 307 10.1557/PROC-663-307.

[ref21] Zagorac D.; Müller H.; Ruehl S.; Zagorac J.; Rehme S. Recent developments in the Inorganic Crystal Structure Database: theoretical crystal structure data and related features. J. Appl. Crystallogr. 2019, 52 (5), 918–925. 10.1107/S160057671900997X. 31636516 PMC6782081

[ref22] Robinson K.; Gibbs G. V.; Ribbe P. H. The structure of zircon: a comparison with garnet. Am. Mineral. 1971, 56, 782–790.

[ref23] Finger L. W. Refinement of the crystal structure of zircon. Carnegie Inst. Wash. Yearbook 1974, 73, 544 547.

[ref24] Siggel A.; Jansen M. Röntgenographische untersuchungen zur bestimmung der einbauposition von seltenen erden (Pr, Tb) und vanadium in zirkonpigmenten. Zeitschrift für anorganische und allgemeine Chemie 1990, 583, 67–77. 10.1002/zaac.19905830109.

[ref25] Mursic Z.; Vogt T.; Boysen H.; Frey F. Single-crystal neutron diffraction study of metamict zircon up to 2000 K. J. Appl. Crystallogr. 1992, 25, 519–523. 10.1107/S0021889892002577.

[ref26] Finch R. J.; Hanchar J. M.; Hoskin P. W. O.; Burns P. C. Rare-earth elements in synthetic zircon: Part 2. A single-crystal X-ray study of xenotime substitution. Am. Mineral. 2001, 86, 681–689. 10.2138/am-2001-5-608.

[ref27] Yu S. C.; Tung S. F.; Lee J. S.; Bai W. J.; Yang J. S.; Fang Q. S.; Zhang Z. M.; Kuo C. T. Structural and spectroscopic features of mantle-derived zircon crystals from Tibet. Western Pac. Earth Sci. 2001, 1, 47–58.

[ref28] Hazen R. M.; Finger L. W. Crystal structure and compressibility of zircon at high pressure. Am. Mineral. 1979, 64, 196–201.

[ref29] Kolesov B. A.; Geiger C. A.; Armbruster T. The dynamic properties of zircon studied by single-crystal X-ray diffraction and Raman spectroscopy. European Journal of Mineralogy 2001, 13, 939–948. 10.1127/0935-1221/2001/0013-0939.

[ref30] Hassel O. XIV. Die Kristallstruktur einiger Verbindungen von der Zusammensetzung MRO4. I. Zirkon ZrSiO4. Z. Kristallogr.-Cryst. Mater. 1926, 63, 247–254. 10.1524/zkri.1926.63.1.247.

[ref31] Wyckoff R. W. G.; Hendricks S. B. IV. Die Kristallstruktur von Zirkon und die Kriterien für spezielle Lagen in tetragonalen Raumgruppen. Zeitschrift für Kristallographie-Crystalline Materials 1928, 66, 73–102. 10.1524/zkri.1928.66.1.73.

[ref32] Binks W. The crystalline structure of zircon. Mineralogical magazine and journal of the Mineralogical Society 1926, 21, 176–187. 10.1180/minmag.1926.021.115.06.

[ref33] Krstanović I. R. Redetermination of the oxygen parameters in zircon (ZrSiO4). Acta Crystallogr. 1958, 11, 896–897. 10.1107/S0365110X58002553.

[ref34] Torres F. J.; Tena M. A.; Alarcón J. Rietveld refinement study of vanadium distribution in V+4–ZrSiO4 solid solutions obtained from gels. Journal of the European Ceramic Society 2002, 22, 1991–1994. 10.1016/S0955-2219(01)00521-0.

[ref35] Chaplot S. L.; Mittal R.; Busetto E.; Lausi A. Thermal expansion in zircon and almandine: Synchrotron x-ray diffraction and lattice dynamical study. Phys. Rev. B 2002, 66, 064302 10.1103/PhysRevB.66.064302.

[ref36] Kittiauchawal T.; Mungchamnankit A.; Sujinnapram S.; Kaewkhao J.; Limsuwan P. The Effect of Heat Treatment on Crystal Structure in Zircon Monitored by ESR and XRD. Procedia Engineering 2012, 32, 706–713. 10.1016/j.proeng.2012.02.001.

[ref37] Onken H.; Vierheilig K.; Hahn H. Über Silicid-und Germanidchalkogenide des Zirkons und Hafniums. Zeitschrift für anorganische und allgemeine Chemie 1964, 333, 267–279. 10.1002/zaac.19643330414.

[ref38] Speer J. A.; Cooper B. J. Crystal structure of synthetic hafnon, HfSiO4, comparison with zircon and the actinide orthosilicates. Am. Mineral. 1982, 67, 804–808.

[ref39] Fuhrmann J.; Pickardt J. Bildung von HfSiO4-Einkristallen durch chemische Transportreaktion. Zeitschrift für anorganische und allgemeine Chemie 1986, 532, 171–174. 10.1002/zaac.19865320123.

[ref40] Boakye E.; Hay R. S.; Petry M. D.; Parthasarathy T. A. Sol-Gel Synthesis of Zircon-Carbon Precursors and Coatings of Nextel 720 Fiber Tows. In 23rd Annual Conference on Composites, Advanced Ceramics, Materials, and Structures: A: Ceramic Engineering and Science Proceedings, Eds. Ustundag E.; Fischman G. 1999.

[ref41] Chen T.; Zhang X.; Jiang W.; Liu J.; Jiang W.; Xie Z. Synthesis and application of C@ZrSiO4 inclusion ceramic pigment from cotton cellulose as a colorant. Journal of the European Ceramic Society 2016, 36, 1811–1820. 10.1016/j.jeurceramsoc.2015.07.021.

[ref42] Fang P.-Y.; Wu J. Low Temperature Synthesis of high purity Zircon Powder by a hydrothermal Method. J. Chin. Ceram. Soc. 2009, 2, 304–316.

[ref43] Heydari H.; Naghizadeh R.; Samimbanihashemi H. R.; Hosseini-Zori M. Synthesis and characterisation of hematite-zircon nanocomposite by sol-gel method. Advanced Materials Research 2013, 829, 544–548. 10.4028/www.scientific.net/AMR.829.544.

[ref44] Hirano M.; Morikawa H.; Inagaki M.; Toyoda M. Direct Synthesis of New Zircon-Type ZrGeO4 and Zr (Ge, Si) O4 Solid Solutions. J. Am. Ceram. Soc. 2002, 85, 1915–1920. 10.1111/j.1151-2916.2002.tb00380.x.

[ref45] Lei B.; Peng C.; Wu J. Controllable synthesis of layered zircons by low-temperature hydrothermal method. J. Am. Ceram. Soc. 2012, 95, 2791–2794. 10.1111/j.1551-2916.2012.05362.x.

[ref46] Tartaj P.; Sanz J.; Serna C. J.; Ocana M. Zircon formation from amorphous spherical ZrSiO4 particles obtained by hydrolysis of aerosols. J. Mater. Sci. 1994, 29, 6533–6538. 10.1007/BF00354017.

[ref47] Wang F.; Liu D. W.; Zhu J. F.; Li D. Microwave Hydrothermal Synthesis of ZrSiO4 Nano-powders. Advanced materials research 2011, 295, 1485–1488. 10.4028/www.scientific.net/AMR.295-297.1485.

[ref48] Baghramyan V. V.; Sargsyan A. A.; Sargsyan A. S.; Knyayan N. B.; Harutyunyan V. V.; Aleksanyan E. M.; Grigoryan N. E.; Badalyan A. H. Optical Properties and Radiation Resistance of Zirconium Silicate Obtained by Microwave Method. Armen. J. Phys. 2017, 10, 56–63.

[ref49] Mori T.; Yamamura H.; Kobayashi H.; Mitamura T. Preparation of High-Purity ZrSiO4 Powder Using Sol–Gel Processing and Mechanical Properties of the Sintered Body. J. Am. Ceram. Soc. 1992, 75, 2420–2426. 10.1111/j.1151-2916.1992.tb05594.x.

[ref50] Alarcón J. Crystallization behaviour and microstructural development in ZrSiO4 and V-ZrSiO4 solid solutions from colloidal gels. Journal of the European Ceramic Society 2000, 20, 1749–1758. 10.1016/S0955-2219(00)00057-1.

[ref51] Kanno Y. Effect of dopants on the formation of hafnon via a sol-gel route. Journal of materials science letters 1993, 12, 1807–1809. 10.1007/BF00539993.

[ref52] Estevenon P.; Kaczmarek T.; Rafiuddin M. R.; Welcomme É.; Szenknect S.; Mesbah A.; Moisy P.; Poinssot C.; Dacheux N. Soft Hydrothermal Synthesis of Hafnon, HfSiO4. Cryst. Growth Des. 2020, 20, 1820–1828. 10.1021/acs.cgd.9b01546.

[ref53] Dawson P.; Hargreave M. M.; Wilkinson G. R. The vibrational spectrum of zircon (ZrSiO4). Journal of Physics C: Solid State Physics 1971, 4, 240 10.1088/0022-3719/4/2/014.

[ref54] Ramakrishnan S. S.; Gokhale K. V. G. K.; Subbarao E. C. Solid solubility in the system zircon-hafnon. Mater. Res. Bull. 1969, 4, 323–327. 10.1016/0025-5408(69)90036-1.

[ref55] Nicola J. H.; Rutt H. N. A comparative study of zircon (ZrSiO4) and hafnon (HfSiO4) Raman spectra. Journal of Physics C: Solid State Physics 1974, 7, 1381 10.1088/0022-3719/7/7/029.

[ref56] Syme R. W. G.; Lockwood D. J.; Kerr H. J. Raman spectrum of synthetic zircon (ZrSiO4) and thorite (ThSiO4). Journal of Physics C: Solid State Physics 1977, 10, 1335 10.1088/0022-3719/10/8/036.

[ref57] Hoskin P. W. O.; Rodgers K. A. Raman spectral shift in the isomorphous series (Zr1-xHfx) SiO4. Eur. J. Solid State Inorg. Chem. 1996, 33, 1111–1121.

[ref58] Grüneberger A. M.; Schmidt C.; Jahn S.; Rhede D.; Loges A.; Wilke M. Interpretation of Raman spectra of the zircon–hafnon solid solution. European Journal of Mineralogy 2016, 28, 721–733. 10.1127/ejm/2016/0028-2551.

[ref59] Cota A.; Burton B. P.; Chaín P.; Pavón E.; Alba M. D. Solution properties of the system ZrSiO4-HfSiO4: a computational and experimental study. J. Phys. Chem. C 2013, 117, 10013–10019. 10.1021/jp401539g.

[ref60] Cherniak D. J.; Pyle J.; Rakovan J. Synthesis of REE and Y phosphates by Pb-free flux methods and their utilization as standards for electron microprobe analysis and in design of monazite chemical U-Th-Pb dating protocol. Am. Mineral. 2004, 89, 1533–1539. 10.2138/am-2004-1023.

[ref61] Sheldrick G. M. A short history of SHELX. Acta Crystallographica Section A: Foundations of Crystallography 2008, 64, 112–122. 10.1107/S0108767307043930. 18156677

[ref62] Farrugia L. J. WinGX and ORTEP for Windows: an update. J. Appl. Crystallogr. 2012, 45, 849–854. 10.1107/S0021889812029111.

[ref63] Cheary R. W.; Coelho A. A fundamental parameters approach to X-ray line-profile fitting. J. Appl. Crystallogr. 1992, 25, 109–121. 10.1107/S0021889891010804.

[ref64] Coelho A. A. TOPAS and TOPAS-Academic: an optimization program integrating computer algebra and crystallographic objects written in C++. J. Appl. Crystallogr. 2018, 51, 210–218. 10.1107/S1600576718000183.

[ref65] Rietveld H. M. A profile refinement method for nuclear and magnetic structures. Journal of applied Crystallography 1969, 2, 65–71. 10.1107/S0021889869006558.

[ref66] Rietveld H. M. Line profiles of neutron powder-diffraction peaks for structure refinement. Acta Crystallogr. 1967, 22, 151–152. 10.1107/S0365110X67000234.

[ref67] Bondars B.; Heidemane G.; Grabis J.; Laschke K.; Boysen H.; Schneider J.; Frey F. Powder diffraction investigations of plasma sprayed zirconia. J. Mater. Sci. 1995, 30, 1621–1625. 10.1007/BF00375275.

[ref68] Jaffe J. E.; Bachorz R. A.; Gutowski M. Low-temperature polymorphs of ZrO2 and HfO2: A density-functional theory study. Phys. Rev. B 2005, 72, 144107 10.1103/PhysRevB.72.144107.

[ref69] Phillippi C. M.; Mazdiyasni K. S. Infrared and Raman spectra of zirconia polymorphs. J. Am. Ceram. Soc. 1971, 54, 254–258. 10.1111/j.1151-2916.1971.tb12283.x.

[ref70] Pitcher M. W.; Ushakov S. V.; Navrotsky A.; Woodfield B. F.; Li G.; Boerio-Goates J.; Tissue B. M. Energy crossovers in nanocrystalline zirconia. J. Am. Ceram. Soc. 2005, 88, 160–167. 10.1111/j.1551-2916.2004.00031.x.

[ref71] Geiger C. A.; Armbruster T. Mn3Al2Si3O12 spessartine and Ca3Al2Si3O12 grossular garnet: Structural dynamic and thermodynamic properties. Am. Mineral. 1997, 82, 740–747. 10.2138/am-1997-7-811.

[ref72] Lager G. A.; Armbruster T.; Faber J. Neutron and X-ray diffraction study of hydrogarnet Ca3Al2(O4H4)3. Am. Mineral. 1987, 72, 756–765.

[ref73] Caruba R.; Baumer A.; Ganteaume M.; Iacconi P. An experimental study of hydroxyl groups and water in synthetic and natural zircons: a model of the metamict state. Am. Mineral. 1985, 70, 1224–1231.

[ref74] Julg A.; Ozias Y. Stabilization of complex ions by the crystal field: CO3 2-, NO3-, O3-,[(OH) 4]4-,[(OH)3F]4-,[(OH)2F2]4-. Physics and Chemistry of Minerals 1985, 12, 307–310. 10.1007/BF00310344.

[ref75] Strzelecki A. C.; Barral T.; Estevenon P.; Mesbah A.; Goncharov V.; Baker J.; Bai J.; Clavier N.; Szenknect S.; Migdisov A.; Xu H.; Ewing R. C.; Dacheux N.; Guo X. The Role of Water and Hydroxyl Groups in the Structures of Stetindite and Coffinite, MSiO4 (M = Ce, U). Inorg. Chem. 2021, 60, 718–735. 10.1021/acs.inorgchem.0c02757. 33393766

[ref76] Bauer J. D.; Labs S.; Weiss S.; Bayarjargal L.; Morgenroth W.; Milman V.; Perlov A.; Curtius H.; Bosbach D.; Zänker H.; Winkler B. High-pressure phase transition of coffinite, USiO4. J. Phys. Chem. C 2014, 118, 25141–25149. 10.1021/jp506368q.

[ref77] Fuchs L. H.; Gebert E. X-ray studies of synthetic coffinite, thorite and uranothorites. Am. Mineral. 1958, 43, 243–248.

[ref78] Guo X.; Szenknect S.; Mesbah A.; Clavier N.; Poinssot C.; Wu D.; Xu H.; Dacheux N.; Ewing R. C.; Navrotsky A. Energetics of a Uranothorite (Th1–x U x SiO4) Solid Solution. Chem. Mater. 2016, 28, 7117–7124. 10.1021/acs.chemmater.6b03346.

[ref79] Marcial J.; Zhang Y.; Zhao X.; Xu H.; Mesbah A.; Nienhuis E. T.; Szenknect S.; Neuefeind J. C.; Lin J.; Qi L.; Migdisov A. A.; Ewing R. C.; Dacheux N.; McCloy J. S.; Guo X. Thermodynamic non-ideality and disorder heterogeneity in actinide silicate solid solutions. npj Mater. Degrad. 2021, 5, 34 10.1038/s41529-021-00179-0.

[ref80] Taylor M. A. R. K.; Ewing R. C. The crystal structures of the ThSiO4 polymorphs: huttonite and thorite. Acta Crystallographica Section B: Structural Crystallography and Crystal Chemistry 1978, 34, 1074–1079. 10.1107/S0567740878004951.

[ref81] Strzelecki A. C.; Bourgeois C.; Kriegsman K. W.; Estevenon P.; Wei N.; Szenknect S.; Mesbah A.; Wu D.; Ewing R. C.; Dacheux N.; Guo X. Thermodynamics of CeSiO4: Implications for Actinide Orthosilicates. Inorg. Chem. 2020, 59, 13174–13183. 10.1021/acs.inorgchem.0c01476. 32871073

[ref82] Frondel C.; Collette R. L. Hydrothermal synthesis of zircon, thorite and huttonite. Am. Mineral. 1957, 42, 759–765.

[ref83] Mumpton F. A.; Roy R. Hydrothermal stability studies of the zircon-thorite group. Geochim. Cosmochim. Acta 1961, 21, 217–238. 10.1016/S0016-7037(61)80056-2.

[ref84] Nasdala L.; Beran A.; Libowitzky E.; Wolf D. The incorporation of hydroxyl groups and molecular water in natural zircon (ZrSiO4). Am. J. Sci. 2001, 301, 831–857. 10.2475/ajs.301.10.831.

[ref85] Strzelecki A. C.; Zhao X.; Estevenon P.; Xu H.; Dacheux N.; Ewing R. C.; Guo X. Crystal chemistry and thermodynamic properties of zircon structure-type materials. Am. Mineral. 2024, 109 (2), 225–242. 10.2138/am-2022-8632.

