# Supplementary material for: Synthesis and Characterization of Single Crystal Zircon-Hafnon Zr(1–x)Hf(x)SiO4 Solid Solutions and the Comparison with the Reaction Products of a TEOS-Based Hydrothermal Route
Source: ACS Omega. 2024 Mar 25;9(14):15781–803. doi: 10.1021/acsomega.3c06960 (PMC11007697; doi:10.1021/acsomega.3c06960)
Supplement: Supplementary file 1 — ao3c06960_si_001.pdf [file ao3c06960_si_001.pdf]

Associated Content (Supporting Information)

Title

On the synthesis and characterization of single crystal zircon-hafnon $Zr_{(1-x)}Hf_{(x)}SiO_4$ solid solutions and the comparison with the reaction products of a TEOS based hydrothermal route

Authors

Andreas Neumann[1)], Volker Kahlenberg[2)], Ian Lerche[1)], Stefan Stöber[1)*], Herbert Pöllmann (†)[1)]

Affiliations

1) Institute of Geoscience and Geography, Martin-Luther-University Halle-Wittenberg, Von-Seckendorff-Platz 3, D-06120 Halle (Saale), Germany

2) Institute of Mineralogy and Petrography, University of Innsbruck, Innrain 52, A-6020 Innsbruck, Austria

* Corresponding author

Email addresses:

Andreas Neumann: andreas.neumann@geo.uni-halle.de,

Volker Kahlenberg: volker.kahlenberg@uibk.ac.at

Ian Lerche: lercheian@yahoo.com

Stefan. Stöber: stefan.stoeber@geo.uni-halle.de

µXRF

Tables S1-S8 denote the measured elements in atom percent units. Zr/Hf amounts are normalized to unity (=1), i.e. x(Zr) + y(Hf)= 1 with x,y=0 to 1.

In Figures S1-S8 yellow cross symbols indicate the position of the µXRF measurement. The red circle denotes the diameter of the µXRF probe (~ 20 µm)

Table S1: Sample 1:0

Spectr. No	O	Si	Zr	Hf	
10 1	66.698	15.137	18.012	0.152	100.000
10 2	66.694	15.113	18.041	0.152	100.000
10 3	66.695	15.220	17.946	0.139	100.000
10 4	66.694	15.101	18.056	0.149	100.000
Mean	66.695	15.143	18.014	0.148	100.000
St._Dev	0.002	0.047	0.042	0.005	
		x(Zr)/y(Hf)	0.992	0.008	

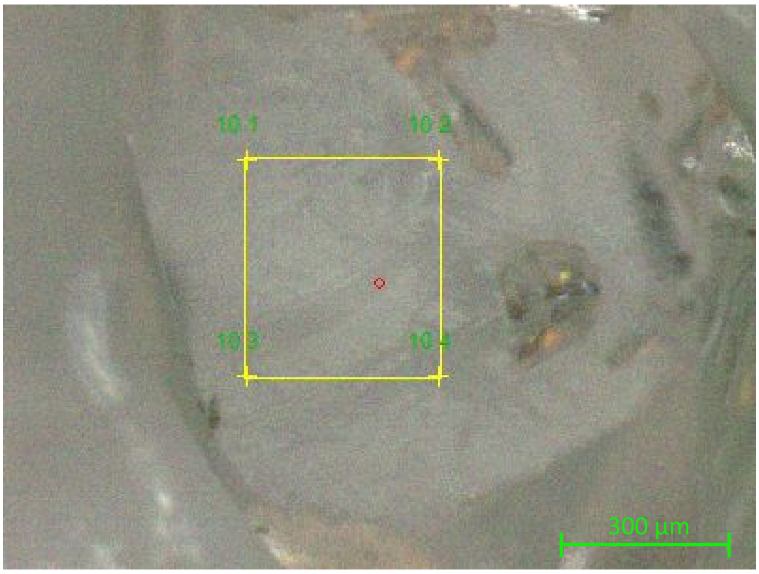

Figure S1

Table S2: Sample 3:1/1

Spectr. No	O	Si	Zr	Hf	
31P1 001	66.695	16.550	13.725	3.030	100.000
31P1 002	66.695	16.041	14.260	3.004	100.000
31P1 003	66.696	15.313	15.050	2.941	100.000
31P1 004	66.694	16.634	13.547	3.126	100.000
31P1 005	66.695	16.814	13.387	3.104	100.000
31P1 006	66.695	16.875	13.340	3.090	100.000
31P1 007	66.701	10.508	19.144	3.647	100.000
31P1 008	66.694	17.167	13.054	3.085	100.000
31P1 009	66.695	16.735	13.432	3.138	100.000
31P1 010	66.700	14.956	15.028	3.316	100.000
31P1 011	66.698	11.691	18.187	3.424	100.000
31P1 012	66.694	17.179	13.040	3.087	100.000
31P1 013	66.695	17.024	13.198	3.083	100.000
31P1 014	66.697	15.900	14.194	3.209	100.000
31P1 015	66.699	12.463	17.293	3.545	100.000
31P1 016	66.696	15.998	13.981	3.325	100.000
31P1 017	66.694	16.966	13.221	3.119	100.000
31P1 018	66.699	14.431	15.401	3.469	100.000
31P1 019	66.696	15.058	14.943	3.303	100.000
Mean	66.696	15.490	14.601	3.213	100.000
St._Dev	0.002	1.900	1.741	0.190	
		x(Zr)/y(Hf)	0.820	0.180	

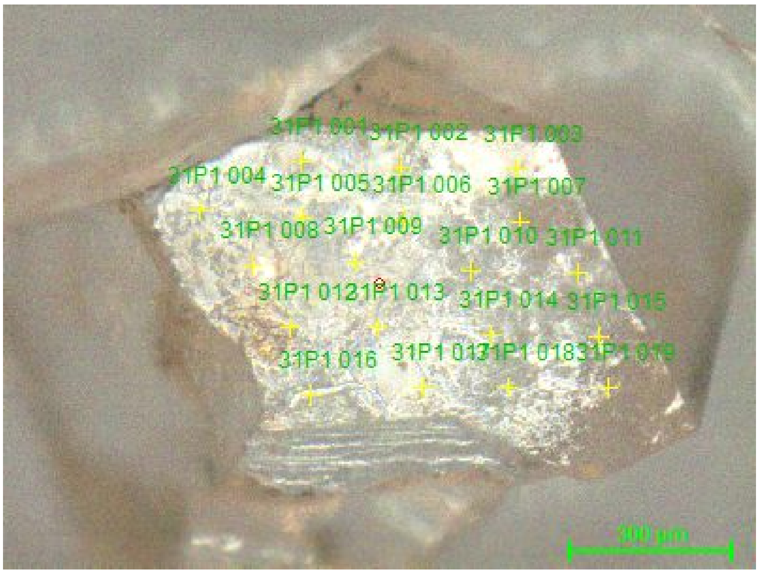

Figure S2

Table S3: Sample 3:1/2

Spectr. No	O	Si	Zr	Hf	
31P2 001	66.691	14.292	14.884	4.133	100.000
31P2 002	66.722	10.896	16.310	6.072	100.000
31P2 003	66.678	5.025	24.368	3.929	100.000
31P2 004	66.673	11.999	17.855	3.472	100.000
31P2 005	66.676	7.002	22.451	3.871	100.000
31P2 006	66.673	15.111	14.947	3.269	100.000
31P2 007	66.672	14.210	15.694	3.424	100.000
31P2 008	66.673	15.823	14.362	3.142	100.000
31P2 009	66.673	15.787	14.471	3.069	100.000
31P2 010	66.672	16.557	13.687	3.084	100.000
31P2 011	66.671	16.990	13.290	3.049	100.000
31P2 012	66.673	17.367	13.044	2.916	100.000
31P2 013	66.671	17.387	13.033	2.909	100.000
Mean	66.678	13.727	16.030	3.565	100.000
St._Dev	0.014	3.806	3.429	0.819	
		x(Zr)/y(Hf)	0.818	0.182	

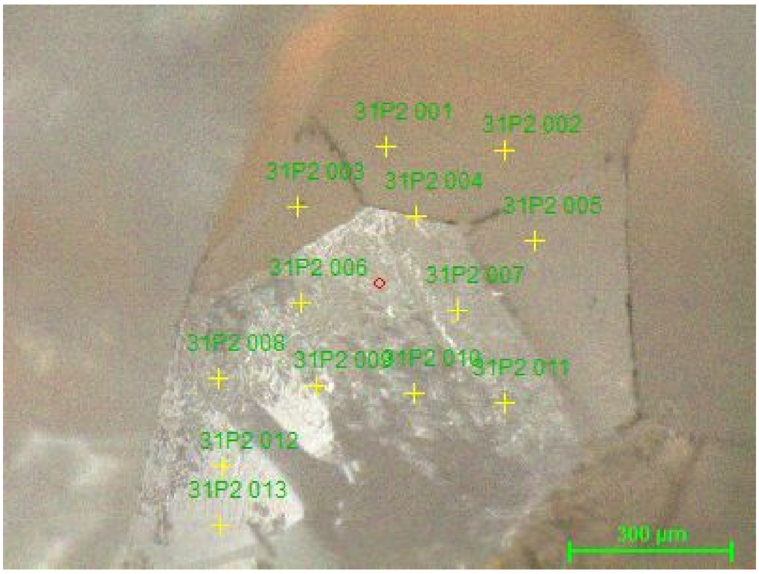

Figure S3

Table S4: Sample 1:1/1

Spectr. No	O	Si	Zr	Hf	
11 001	66.671	14.738	12.349	6.242	100.000
11 002	66.672	15.554	11.488	6.286	100.000
11 003	66.672	16.497	10.657	6.174	100.000
11 004	66.671	15.856	11.090	6.383	100.000
11 005	66.672	17.147	9.948	6.233	100.000
11 006	66.671	16.425	10.776	6.128	100.000
11 007	66.670	16.739	10.665	5.926	100.000
11 008	66.672	17.743	9.308	6.277	100.000
11 009	66.671	17.780	9.282	6.268	100.000
11 010	66.671	17.806	9.236	6.287	100.000
11 011	66.672	17.864	9.175	6.289	100.000
11 012	66.671	16.865	10.011	6.453	100.000
11 013	66.671	16.169	10.914	6.245	100.000
11 014	66.671	17.824	9.199	6.305	100.000
11 015	66.672	16.887	9.938	6.502	100.000
11 016	66.671	17.737	9.167	6.425	100.000
11 017	66.671	17.594	9.246	6.489	100.000
11 018	66.671	17.533	9.267	6.528	100.000
11 019	66.671	17.510	9.540	6.280	100.000
11 020	66.673	17.859	9.139	6.329	100.000
11 021	66.672	17.876	9.102	6.350	100.000
11 022	66.672	17.830	9.114	6.384	100.000
11 023	66.671	17.239	9.464	6.625	100.000
11 024	66.673	16.752	9.934	6.642	100.000
11 025	66.671	17.805	9.155	6.370	100.000
11 026	66.672	17.829	9.089	6.411	100.000
11 027	66.671	17.839	9.054	6.436	100.000
11 028	66.672	17.791	9.072	6.466	100.000
11 029	66.672	17.784	9.098	6.446	100.000
11 030	66.671	17.808	9.064	6.457	100.000
11 031	66.672	16.828	9.905	6.594	100.000
11 032	66.672	17.851	9.105	6.372	100.000
11 033	66.671	17.870	9.116	6.343	100.000
Mean	66.671	17.249	9.717	6.362	100.000
St._Dev	0.001	0.784	0.830	0.144	
		x(Zr)/y(Hf)	0.604	0.396	

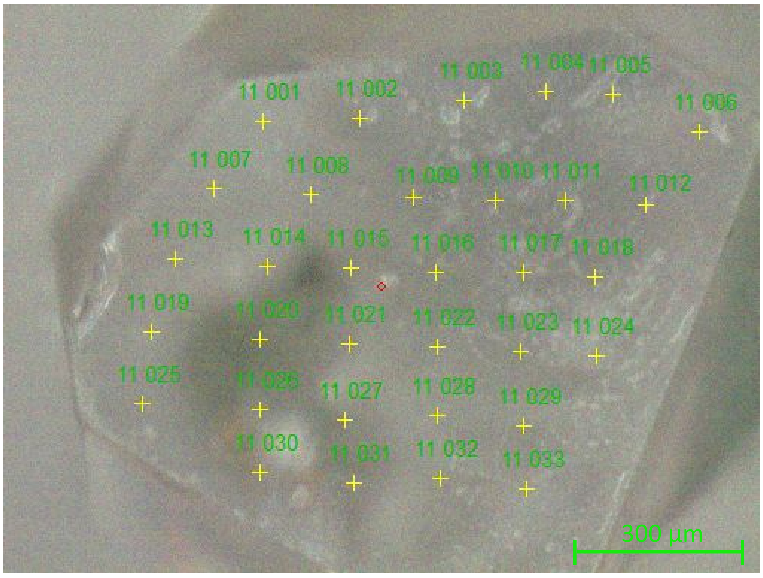
11 001
11 002
11 003
11 004
11 005
11 006
11 007
11 008
11 009
11 010
11 011
11 012
11 013
11 014
11 015
11 016
11 017
11 018
11 019
11 020
11 021
11 022
11 023
11 024
11 025
11 026
11 027
11 028
11 029
11 030
11 031
11 032
11 033
300 µm

Figure S4

Table S5: Sample 1:1/2

Spectr. No	O	Si	Zr	Hf	
11P2 001	66.673	16.126	7.824	9.377	100.000
11P2 002	66.674	16.055	7.491	9.781	100.000
11P2 003	66.675	16.039	7.463	9.823	100.000
11P2 004	66.674	16.243	7.435	9.648	100.000
11P2 005	66.672	16.393	8.024	8.911	100.000
11P2 006	66.673	16.164	7.558	9.605	100.000
11P2 007	66.673	16.417	7.080	9.830	100.000
11P2 008	66.674	16.262	7.410	9.655	100.000
11P2 009	66.675	16.185	7.296	9.845	100.000
11P2 010	66.672	16.143	8.063	9.123	100.000
11P2 011	66.674	14.357	8.202	10.767	100.000
11P2 012	66.674	16.342	7.061	9.923	100.000
11P2 013	66.675	15.945	7.381	9.998	100.000
11P2 014	66.674	16.287	7.198	9.842	100.000
11P2 015	66.674	16.326	7.807	9.193	100.000
11P2 016	66.673	15.557	8.210	9.560	100.000
11P2 017	66.674	15.100	7.450	10.775	100.000
11P2 018	66.675	15.703	7.478	10.145	100.000
11P2 019	66.675	16.333	7.188	9.804	100.000
11P2 020	66.673	15.406	8.384	9.537	100.000
11P2 021	66.674	15.830	7.476	10.020	100.000
11P2 022	66.672	15.418	7.163	10.746	100.000
11P2 023	66.675	15.070	7.627	10.627	100.000
11P2 024	66.674	15.971	7.753	9.602	100.000
11P2 025	66.673	15.342	7.693	10.292	100.000
Mean	66.674	15.881	7.589	9.857	100.000
St._Dev	0.001	0.507	0.360	0.486	
		x(Zr)/y(Hf)	0.435	0.565	

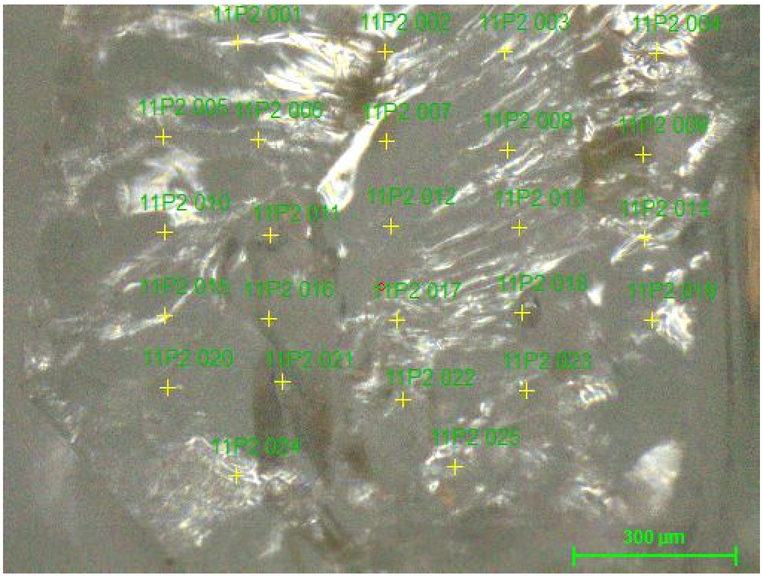
11P2 001
11P2 002
11P2 003
11P2 004
11P2 005
11P2 006
11P2 007
11P2 008
11P2 009
11P2 010
11P2 011
11P2 012
11P2 013
11P2 014
11P2 015
11P2 016
11P2 017
11P2 018
11P2 019
11P2 020
11P2 021
11P2 022
11P2 023
11P2 024
11P2 025
300 µm

Figure S5

Table S6: Sample 1:3/1

Spectr. No	O	Si	Zr	Hf	
13P1 001	66.674	16.275	5.095	11.957	100.000
13P1 002	66.674	16.311	5.087	11.928	100.000
13P1 003	66.674	15.811	5.270	12.246	100.000
13P1 004	66.673	16.497	4.980	11.850	100.000
13P1 005	66.673	15.710	5.269	12.348	100.000
13P1 006	66.674	15.532	5.337	12.458	100.000
13P1 007	66.669	15.620	5.161	12.550	100.000
13P1 008	66.672	15.514	5.367	12.446	100.000
13P1 009	66.675	16.194	4.530	12.600	100.000
13P1 010	66.668	15.519	5.072	12.741	100.000
13P1 011	66.670	15.133	5.299	12.898	100.000
13P1 012	66.674	15.517	4.405	13.405	100.000
Mean	66.672	15.803	5.073	12.452	100.000
St._Dev	0.002	0.401	0.295	0.424	
		x(Zr)/y(Hf)	0.289	0.711	

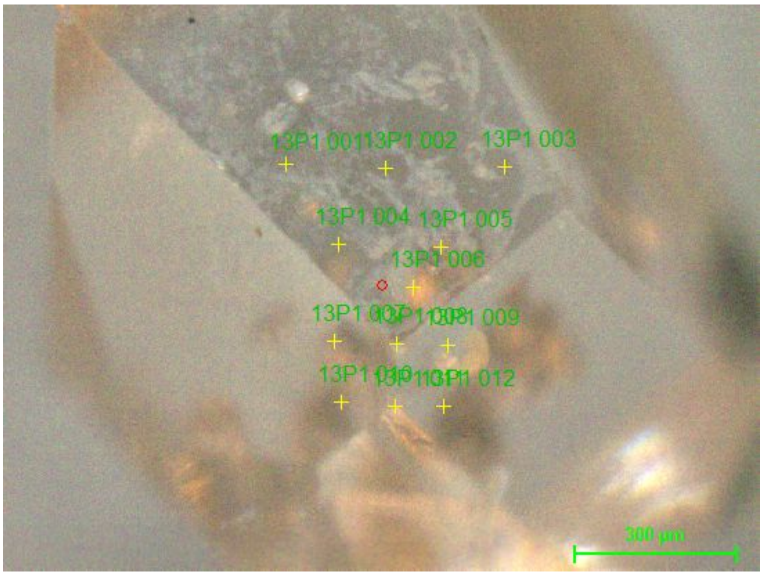

Figure S6

Table S7: Sample 1:3/2

Spectr. No	O	Si	Zr	Hf	
13P2 001	66.685	16.591	4.807	11.917	100.000
13P2 002	66.685	16.583	4.812	11.919	100.000
13P2 003	66.687	16.587	4.816	11.910	100.000
13P2 004	66.687	16.537	4.920	11.855	100.000
13P2 005	66.688	16.511	4.888	11.913	100.000
13P2 006	66.688	16.564	4.802	11.947	100.000
13P2 007	66.690	16.695	4.786	11.830	100.000
13P2 008	66.691	16.573	4.874	11.862	100.000
13P2 009	66.691	15.177	5.928	12.204	100.000
13P2 010	66.688	16.519	4.964	11.829	100.000
13P2 011	66.688	16.527	4.947	11.838	100.000
13P2 012	66.687	16.631	4.777	11.905	100.000
13P2 013	66.689	16.473	4.863	11.975	100.000
13P2 014	66.687	15.656	5.627	12.029	100.000
13P2 015	66.689	16.438	4.986	11.887	100.000
13P2 016	66.689	16.617	4.855	11.839	100.000
13P2 017	66.689	16.510	5.041	11.760	100.000
13P2 018	66.686	15.320	5.981	12.013	100.000
13P2 019	66.693	15.352	5.900	12.055	100.000
Mean	66.688	16.309	5.083	11.920	100.000
St._Dev	0.002	0.491	0.412	0.099	
		x(Zr)/y(Hf)	0.299	0.701	

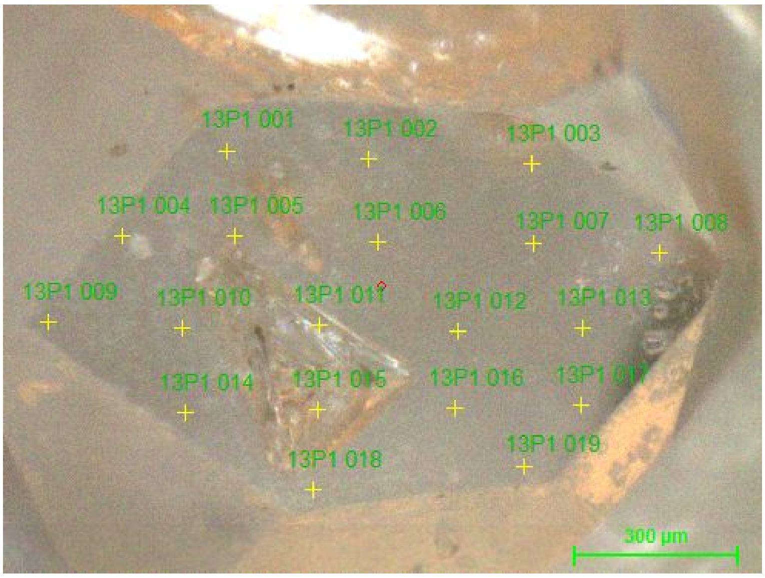

Figure S7

Table S8: Sample 0:1

Spectr. No	O	Si	Zr	Hf	
01 1	66.706	17.010	0.124	16.160	100.000
01 2	66.706	16.812	0.092	16.391	100.000
01 3	66.708	16.149	0.104	17.038	100.000
01 4	66.711	16.450	0.102	16.737	100.000
Mean	66.708	16.605	0.105	16.582	100.000
St._Dev	0.002	0.331	0.012	0.334	
		x(Zr)/y(Hf)	0.006	0.994	

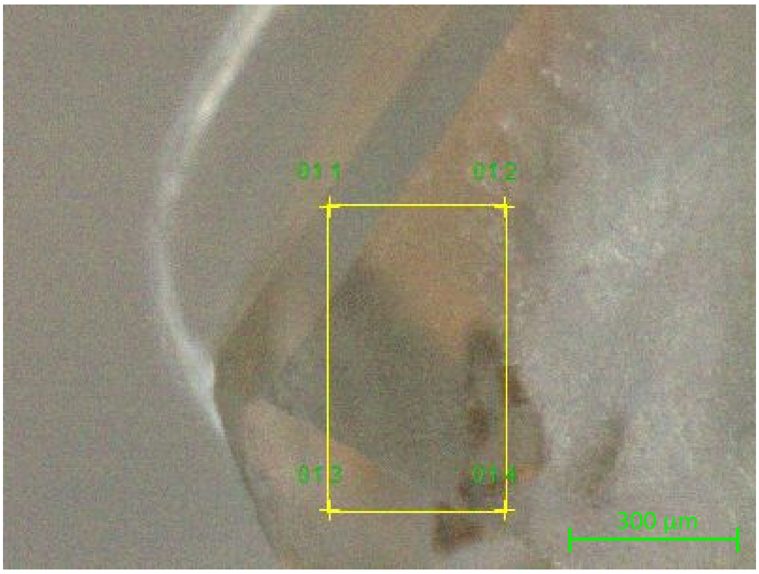

Figure S8

XRD

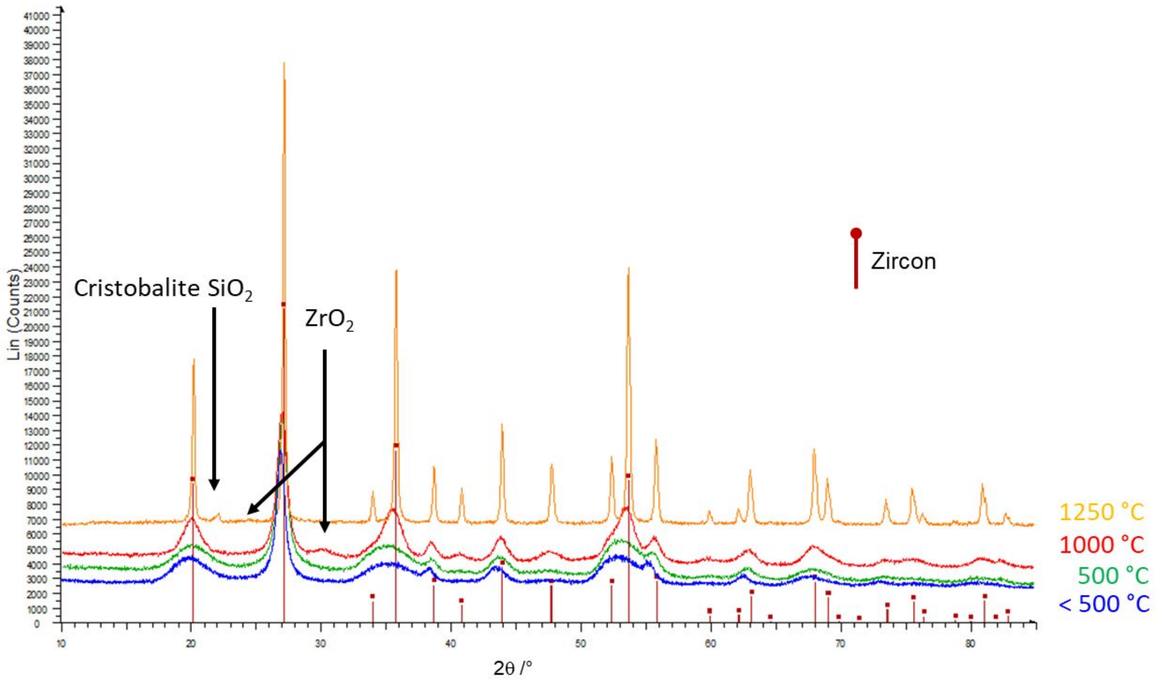

Figure S9: Phase evolution during heat treatment of hydrothermal zircon at 500 °, 1000 °C, and 1250 °C

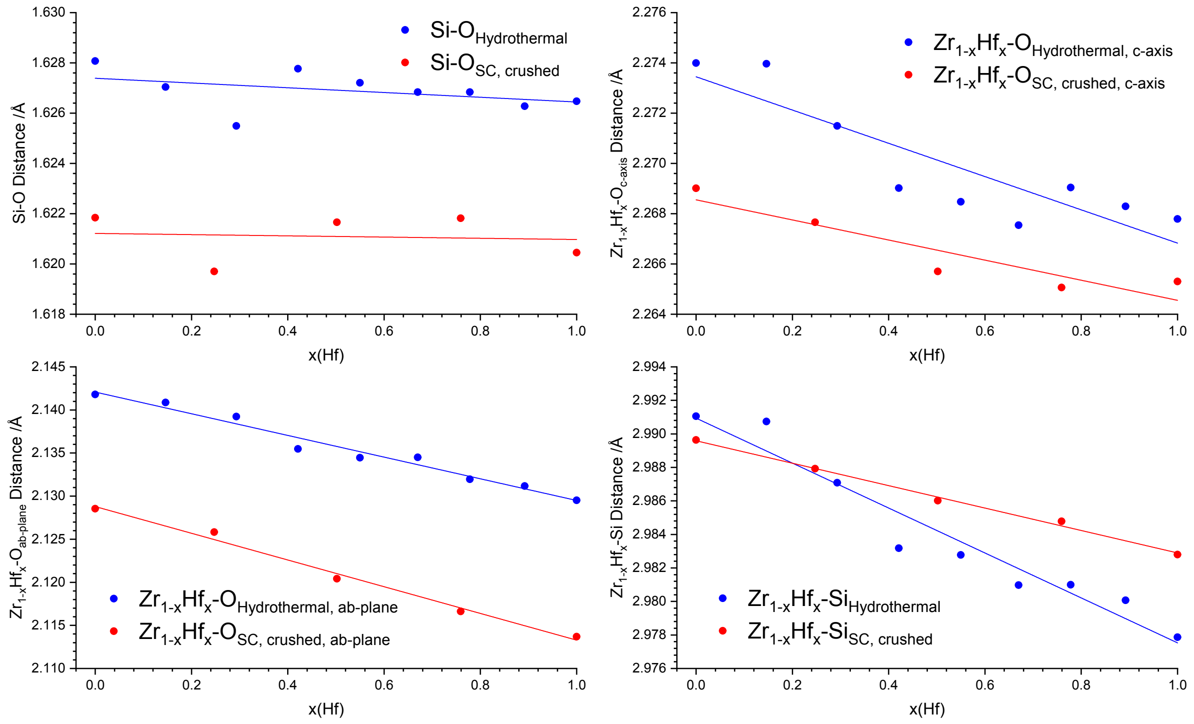

Figure S10: a) Interatomic distance between Si and O (SiO_4 tetrahedron) b) $Zr_{1-x}Hf_x$ and O, connecting the tetrahedra via common edges in c- direction c) $Zr_{1-x}Hf_x$ and O, connecting the tetrahedra via bridging O with $Zr_{1-x}Hf_x$ (ab-plane) d) Interatomic distance between $Zr_{1-x}Hf_x$ and Si

Table S9 (Part 1): Lattice constants – literature comparison zircon, hafnon listed in the ICSD database 2018/2 (cf. [1]) for zircon and hafnon

No	a	c	c/a	Vol_UC	Zircon/Hafnon	Reference	Quality
1	6.6120	5.9940	0.90653	262.049	Zircon	Finger 1974 [2]	*
2	6.6070	5.9820	0.90540	261.129	Zircon	Robinson 1971 [3]	*
3	6.6216	6.0018	0.90640	263.152	Zircon	Siggel 1990 [4]	*
4	6.6056	5.9820	0.90560	261.018	Zircon	Siggel 1990 [4]	*
5	6.6052	5.9802	0.90538	260.908	Zircon	Siggel 1990 [4]	*
6	6.6100	6.0010	0.90787	262.196	Zircon	Mursic 1992 [5]	*
7	6.6080	6.0020	0.90829	262.081	Zircon	Mursic 1992 [5]	*
8	6.5400	6.0330	0.92248	258.041	Zircon	Mursic 1992 [5]	*
9	6.6490	6.0400	0.90841	267.024	Zircon	Mursic 1992 [5]	*
10	6.6102	5.9860	0.90557	261.557	Zircon	Finch 2001 [6]	*
11	6.6139	5.9850	0.90491	261.806	Zircon	Finch 2001 [6]	*
12	6.5980	5.9740	0.90543	260.070	Zircon	Yu 2001 [7]	*
13	6.6042	5.9796	0.90542	260.803	Zircon	Hazen 1979 [8]	*
14	6.5927	5.9742	0.90618	259.661	Zircon	Hazen 1979 [8]	*
15	6.5849	5.9693	0.90651	258.834	Zircon	Hazen 1979 [8]	*
16	6.5808	5.9670	0.90673	258.412	Zircon	Hazen 1979 [8]	*
17	6.5737	5.9638	0.90722	257.717	Zircon	Hazen 1979 [8]	*
18	6.5650	5.9583	0.90759	256.798	Zircon	Hazen 1979 [8]	*
19	6.5590	5.9552	0.90794	256.196	Zircon	Hazen 1979 [8]	*
20	6.5531	5.9519	0.90826	255.593	Zircon	Hazen 1979 [8]	*
21	6.6039	5.9783	0.90527	260.723	Zircon	Kolesov 2001 [9]	*
Mean	6.5951	5.9837	0.90730	260.275			
22	6.5930	5.9400	0.90096	258.198	Zircon	Hassel 1926 [10]	
23	6.6100	5.9800	0.90469	261.279	Zircon	Wyckoff 1927 [11]	
24	6.5800	5.9300	0.90122	256.748	Zircon	Binks 1926 [12]	
25	6.6220	6.0240	0.90969	264.158	Zircon	Krstanovic 1964 [13]	
26	6.6164	6.0150	0.90910	263.317	Zircon	Krstanovic 1958 [13]	
27	6.6004	5.9785	0.90578	260.455	Zircon	Torres 2002 [14]	
28	6.5991	5.9841	0.90681	260.596	Zircon	Chaplot 2002 [15]	
29	6.6007	5.9866	0.90696	260.832	Zircon	Chaplot 2002 [15]	
30	6.5963	5.9824	0.90693	260.301	Zircon	Chaplot 2002 [15]	
31	6.5989	5.9857	0.90708	260.650	Zircon	Chaplot 2002 [15]	
32	6.6017	5.9778	0.90549	260.527	Zircon	Kittiauchawal 2012 [16]]	
33	6.6040	5.9795	0.90544	260.783	Zircon	Kittiauchawal 2012 [16]]	
34	6.6403	5.9262	0.89246	261.307	Zircon	Valero 1998 [17]	
35	6.5180	5.8600	0.89905	248.958	Zircon	Grüneberger 2016 [18]	
36	6.6490	5.9280	0.89156	262.072	Zircon	Grüneberger 2016 [18]	
Mean	6.6020	5.9652	0.90355	260.012			
37	6.5725	5.9632	0.90730	257.597	Hafnon	Speer 1982 [19]	*
38	6.5800	5.9800	0.90881	258.912	Hafnon	Fuhrmann 1986 [20]	*
Mean	6.5763	5.9716	0.90806	258.2547			

Table S9 (Part 2): Lattice constants – literature comparison zircon, hafnon, and $Zr_{1-x}Hf_xSiO_4$

No	a	c	c/a	Vol_UC	Zircon/Hafnon	Reference	Quality
39	6.4940	5.8600	0.90237	247.128	Hafnon	Grüneberger 2016 [18]	
40	6.5970	5.9180	0.89707	257.554	Hafnon	Grüneberger 2016 [18]	
Mean	6.5455	5.8890	0.89972	252.341			
					Solid Solutions		
41	6.6060	5.9830	0.90569	261.094	Zr/Hf 1:0	Cota 2013 [21]	
42	6.5970	5.9780	0.90617	260.165	Zr/Hf 3:1	Cota 2013 [21]	
43	6.5860	5.9740	0.90708	259.125	Zr/Hf 1:1	Cota 2013 [21]	
44	6.5780	5.9710	0.90772	258.366	Zr/Hf 1:3	Cota 2013 [21]	
45	6.5760	5.9700	0.90785	258.165	Zr/Hf 0:1	Cota 2013 [21]	
46	6.6030	5.9810	0.90580	260.769	Zr/Hf 1:0	Ramakrishnan 1969 [22]	
47	6.5960	5.9800	0.90661	260.173	Zr/Hf 4:1	Ramakrishnan 1969 [22]	
48	6.5930	5.9780	0.90672	259.850	Zr/Hf 3:2	Ramakrishnan 1969 [22]	
49	6.5850	5.9740	0.90721	259.046	Zr/Hf 2:3	Ramakrishnan 1969 [22]	
50	6.5780	5.9710	0.90772	258.366	Zr/Hf 1:4	Ramakrishnan 1969 [22]	
51	6.5690	5.9670	0.90836	257.487	Zr/Hf 0:1	Ramakrishnan 1969 [22]	
					pH	Estevenon 2020 [23]	
52	6.5946	5.9605	0.90385	259.215	HCl 1,5M	Estevenon 2020 [23]	
53	6.5935	5.9594	0.90383	259.080	HCl 1,0M	Estevenon 2020 [23]	
54	6.5956	5.9561	0.90304	259.102	0,5	Estevenon 2020 [23]	
55	6.5988	5.9544	0.90235	259.279	1	Estevenon 2020 [23]	
56	6.5869	5.9575	0.90445	258.480	1,6	Estevenon 2020 [23]	
57	6.5945	5.9583	0.90353	259.111	2	Estevenon 2020 [23]	
58	6.5912	5.9555	0.90355	258.730	3	Estevenon 2020 [23]	
59	6.5940	5.9470	0.90188	258.581	5	Estevenon 2020 [23]	
					c(Hf)		
60	6.5939	5.9542	0.9030	258.886	8.4 x 10-3 mol·L-1	Estevenon 2020 [23]	
61	6.5913	5.9602	0.9043	258.942	4.2 x 10-2 mol·L-1	Estevenon 2020 [23]	
62	6.5995	5.9575	0.9027	259.469	1.0 mol·L-1	Estevenon 2020 [23]	
63	6.6447	5.9801	0.9000	264.034	150 °C	Estevenon 2020 [23]	
64	6.6217	5.9511	0.89873	260.937	200 °C	Estevenon 2020 [23]	
65	6.5961	5.9611	0.90373	259.359	7d	Estevenon 2020 [23]	
66	6.5935	5.9606	0.90401	259.133	20d	Estevenon 2020 [23]	
67	6.5714	5.9685	0.90825	257.740	TGA 1000 °C	Estevenon 2020 [23]	

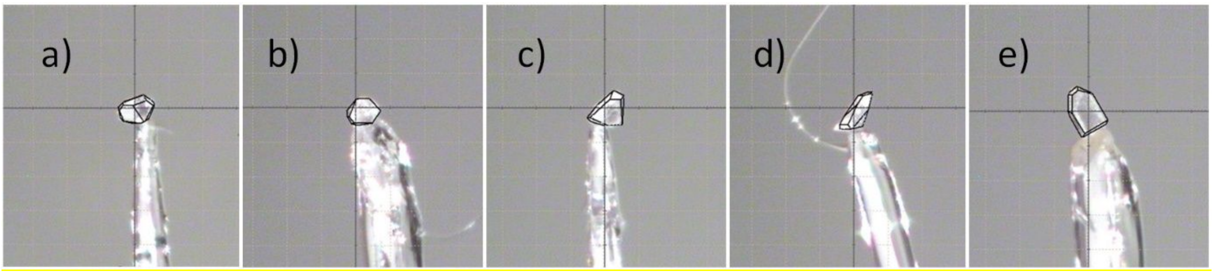

Figure S11: Images of zircon-hafnon solid solution single crystals mounted on capillaries used for single crystal analysis – grid distance equals 100 µm. a) Zr/Hf = 1:0, b) Zr/Hf = 3:1, c) Zr/Hf = 1:1, d) Zr/Hf = 1:3, e) Zr/Hf = 0:1

Table S10: R2 values for a, c, c/a, and unit cell volume for Single Crystal XRD, crushed single PXRD, hydrothermal synthesis, and TG based on the linear fit in Figure 7

	Single Crystal	Crushed Single Crystal PXRD	Hydrothermal PXRD	TG XRD
a	0.94300	0.99338	0.98438	0.98448
c	0.70402	0.99628	0.93934	0.53575
c/a	0.95178	0.97604	0.73191	0.74970
Vol UC	0.91889	0.99507	0.98175	0.98979

Table S11a: Crystallite size of crushed single crystals of the zircon-hafnon solid solutions dependence on the Miller indices

	1:0 (1,000)	±	3:1 (0,250)	±	1:1 (0.500)	±	1:3 (0,750)	±	0:1 (1,000)	±
h00	246.232	15.193	119.107	4.796	107.637	4.432	91.34275	5.27666	81.527	3.125
00l	66.364	34.864	31.956	13.840	31.956	7.411	31.97538	18.10816	31.956	8.531
hkl	118.064	13.042	94.619	10.270	94.762	9.517	85.18107	8.5655	85.720	6.478
hlk	126.199	6.672	91.041	4.088	107.607	5.259	89.33124	5.71713	89.027	4.075
lkh	111.247	10.164	79.086	7.444	112.155	12.461	79.74060	10.38875	74.653	6.058
h0l	138.638	14.840	98.872	8.721	114.202	13.368	69.68882	9.25423	67.017	6.169
l0h	111.935	16.582	92.781	12.372	110.681	19.443	79.25656	13.68258	43.922	3.158
0kl	138.638	14.840	98.872	8.721	114.202	13.368	69.68882	9.25423	67.017	6.169
hk0	106.422	14.859	73.007	9.097	97.378	13.607	80.84499	12.29402	88.081	9.827
hh0	87.151	14.918	75.978	10.064	77.046	9.334	103.56419	23.65887	101.484	15.422
hkk	108.458	19.840	123.389	23.032	155.496	33.057	100.44462	15.72397	91.066	9.634
kkh	150.487	12.636	78.630	5.041	111.788	11.234	104.64963	11.34078	84.964	4.538
h0h	210.168	30.733	139.885	12.490	142.767	15.778	120.50098	13.58767	109.365	8.924

Table S11b: Crystallite size of hydrothermal synthesis zircon-hafnon solid solutions (HT) dependence on the Miller indices – Part 1 (Zr/Hf: 1:0 – 1:1)

	1:0 (1,000)	±	7:1 (0,125)	±	3:1 (0,250)	±	5:3 (0,375)	±	1:1 (0,500)	±
h00	10.345	0.139	9.436	0.119	8.944	0.127	8.964	0.133	8.122	0.111
00l	0.818	6.857	0.794	0.356	0.646	5.720	1.293	8.397	1.477	6.813
hkl	4.267	0.214	4.278	0.186	4.036	0.201	4.127	0.181	3.945	0.152
hlk	2.848	0.049	2.864	0.047	2.659	0.057	2.774	0.061	2.560	0.045
lkh	2.454	0.092	2.462	0.094	2.313	0.115	2.492	0.122	2.247	0.125
h0l	4.938	0.157	4.775	0.142	4.529	0.142	4.616	0.147	4.273	0.127
l0h	1.906	0.105	1.986	0.100	1.824	0.112	1.999	0.125	1.841	0.096
0kl	4.938	0.157	4.775	0.142	4.529	0.142	4.616	0.147	4.273	0.127
hk0	6.261	0.366	6.098	0.364	5.790	0.334	5.975	0.439	5.357	0.475
hh0	7.589	0.335	6.653	0.303	6.572	0.308	6.547	0.304	6.443	0.264
hkk	4.730	0.460	4.675	0.322	4.286	0.269	4.394	0.246	4.167	0.191
kkh	2.093	0.048	2.073	0.043	1.952	0.041	2.010	0.038	1.879	0.033
h0h	2.583	0.109	2.598	0.060	2.351	0.072	2.372	0.070	2.233	0.056

Table S11b: Crystallite size of hydrothermal synthesis Zircon-Hafnon solid solutions (HT) dependence on the Miller indices – Part 2 (Zr/Hf: 3:5 – 0:1)

	3:5 (0,625)	±	1:3 (0,750)	±	1:7 (0,875)	±	0:1 (1,000)	±
h00	8.556	0.116	8.168	0.115	8.680	0.120	8.250	0.115
00l	1.037	7.646	1.030	0.788	1.220	7.269	1.080	7.815
hkl	3.996	0.150	3.862	0.148	4.048	0.147	3.989	0.144
hlk	2.605	0.046	2.501	0.044	2.556	0.046	2.481	0.051
lkh	2.183	0.112	2.104	0.134	2.205	0.127	2.089	0.124
h0l	4.310	0.124	4.114	0.129	4.227	0.127	4.049	0.115
l0h	1.909	0.098	1.738	0.091	1.786	0.096	1.715	0.099
0kl	4.310	0.124	4.114	0.129	4.227	0.127	4.049	0.115
hk0	5.451	0.301	5.461	0.321	5.534	0.420	5.298	0.297
hh0	6.417	0.252	6.346	0.247	6.856	0.225	6.603	0.242
hkk	3.994	0.173	3.880	0.155	3.992	0.149	3.787	0.137
kkh	1.890	0.039	1.823	0.038	1.874	0.039	1.784	0.037
h0h	2.257	0.055	2.168	0.053	2.255	0.049	2.090	0.048

Table S11c: Crystallite size of Zircon-Hafnon solid solutions of hydrothermal synthesis being subjected to TG analysis (1250 °C) dependence on the Miller indices

	1:0 (1,000)	±	3:1 (0,250)	±	1:1 (0,500)	±	1:3 (0,750)	±	0:1 (1,000)	±
h00	9.078	0.180	8.024	0.335	9.749	0.287	7.144	0.312	8.030	0.143
00l	6.108	2.635	3.681	3.869	5.347	2.448	5.833	3.943	6.893	2.203
hkl	7.704	0.656	6.535	1.033	8.391	0.797	6.062	1.064	6.604	0.375
hlk	6.954	0.220	6.120	0.333	7.787	0.335	5.329	0.323	6.085	0.162
lkh	6.114	0.429	5.641	1.307	8.164	0.587	5.675	0.633	5.605	0.248
h0l	8.443	0.610	7.022	0.737	8.234	0.746	6.813	1.011	7.174	0.373
l0h	6.713	0.606	4.412	0.657	7.403	0.790	5.047	0.830	5.143	0.367
0kl	8.443	0.610	7.022	0.737	8.234	0.746	6.813	1.011	7.174	0.373
hk0	7.410	0.694	7.131	1.201	8.807	1.187	6.463	1.140	7.872	0.659
hh0	9.436	1.148	8.051	1.139	9.688	1.235	8.386	2.059	8.018	0.612
hkk	7.902	1.085	7.604	1.431	8.144	0.771	6.139	0.743	6.619	0.289
kkh	6.170	0.222	5.341	0.289	7.061	0.323	5.356	0.415	5.191	0.146
h0h	6.036	0.314	5.246	0.347	7.058	0.398	4.614	0.342	4.841	0.135

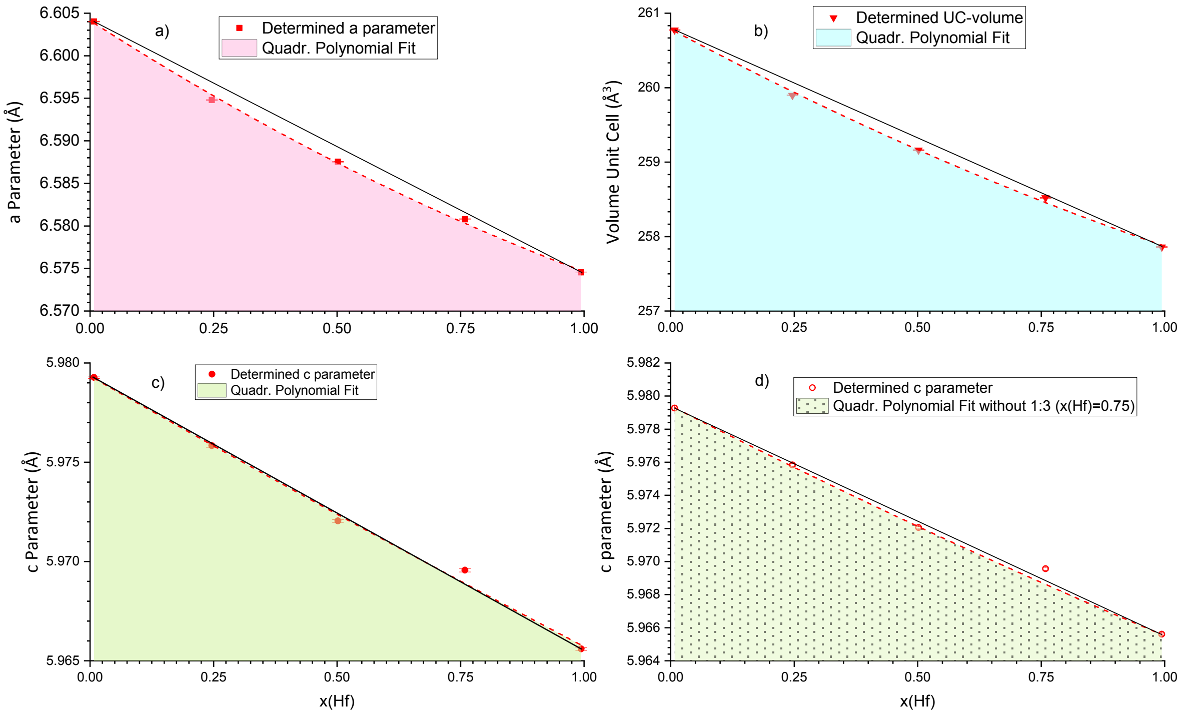

Figure S12: Negative deviation of the lattice constants from linear behavior of the crushed single crystals, a) a parameter, b) Unit cell volume UC, c) c parameter with data points for x(Hf) considered for fitting, d) c parameter without x(Hf)=0.75, i.e. 1:3.

Single crystal zircon-hafnon data solid solution series are provided separately in attached CIF-Files

Raman

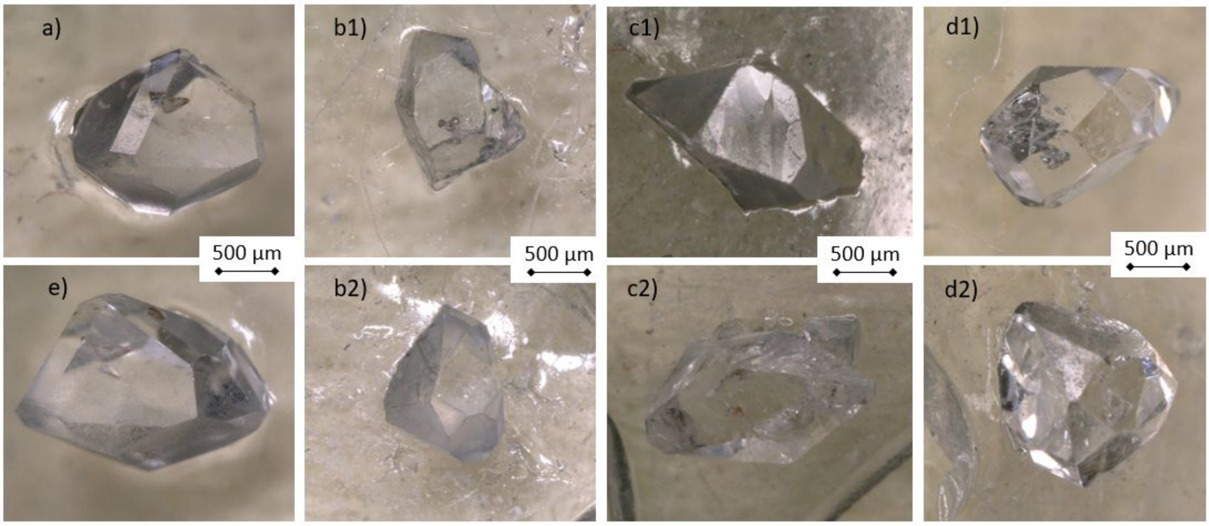

Figure S13: Images of zircon-hafnon solid solution single crystals glued on sample holders used for Raman and µXRF analyses (ref. Figure S1-S8). a) Zr/Hf = 1:0, b1/b2) Zr/Hf = 3:1, c1/c2) Zr/Hf = 1:1, d1/d2) Zr/Hf = 1:3, e) Zr/Hf = 0:1.

Table S12-S22 denote the fitted results for the observed Raman modes of the single crystals (cf. Figure S13) of the zircon-hafnon solid solution series

Table S12: Observed Raman modes of $ZrSiO_4$ (cf. Figure S13)

Sample 1:0	
Ideal	0
Weighed	0.008
µXRF	0.008
Eg(II)	201.408
±	0.045
B1g(II), Mode 207	213.975
±	0.023
Eg(III)	224.399
±	0.041
B2g(v2)	
Eg(I)	356.030
±	0.025
B1g(I)	392.564
±	0.104
A1g(v2)	438.602
±	0.018
Eg(v4)	548.390
±	1.481
B1g(v4)	640.453
±	0.533
Eg(v3)	
A1g(v1)	974.080
±	0.019
B1g(v3)	1007.610
±	0.006

Table S13: Observed Raman modes from sample 3:1/1 (cf. Figure S13) – 5 measurements 1a-e

Sample 3:1/1						
					Ideal	0.25
					Weighed	0.2467
					µXRF	0.18
	1a	1b	1c	1d	1e	Mean
Eg(II)						
B1g(II), Mode 207						
Eg(III)	220.806	220.823	220.990	220.668	219.819	220.621
±	0.040	0.042	0.037	0.049	0.066	0.047
B2g(v2)	265.999	264.658	266.000	266.000	264.000	265.331
±	0.741	0.960	0.922	0.724	1.079	0.885
Eg(I)	354.786	355.011	354.944	354.849	354.230	354.764
±	0.012	0.012	0.011	0.013	0.014	0.012
B1g(I)	394.164	394.449	393.829	394.086	394.197	394.145
±	0.351	0.471	0.403	0.403	0.503	0.426
A1g(v2)	440.433	440.353	440.061	440.387	440.176	440.282
±	0.014	0.015	0.014	0.016	0.017	0.015
Eg(v4)	546.001	546.001	546.431	546.612	546.005	546.210
±	0.618	0.679	0.692	0.887	0.675	0.710
B1g(v4)	640.491	640.763	640.999	640.119	639.207	640.316
±	87.821	0.793	8.152	8.358	0.714	21.168
Eg(v3)	926.041	925.699	925.071	926.005	925.785	925.720
±	0.351	0.295	0.339	0.379	0.467	0.366
A1g(v1)	976.000	975.982	975.669	976.000	975.907	975.912
±	0.028	0.028	0.028	0.030	0.030	0.029
B1g(v3)	1010.250	1010.247	1009.793	1010.357	1010.222	1010.174
±	0.012	0.013	0.012	0.014	0.014	0.013

Table S14: Observed Raman modes from sample 3:1/2 (cf. Figure S13) – 5 measurements 2a-e

Sample 3:1/2						
Ideal					Ideal	0.25
Weighed					Weighed	0.2467
µXRF					µXRF	0.182
	2a	2b	2c	2d	2e	Mean
Eg(II)						
B1g(II), Mode 207						
Eg(III)	220.235	220.228	220.588	220.209	220.294	220.311
±	0.226	0.291	0.136	0.220	0.154	0.205
B2g(v2)	265.208	264.750	267.414	264.376	264.376	265.225
±	25.724	0.361	1.326	0.275	0.275	5.592
Eg(I)	354.561	354.805	354.383	354.556	354.308	354.523
±	0.043	0.046	0.030	0.045	0.032	0.039
B1g(I)	393.923	393.647	393.546	393.815	393.414	393.669
±	0.135	0.162	0.152	0.154	0.176	0.156
A1g(v2)	440.181	439.997	439.908	439.977	439.699	439.952
±	0.022	0.024	0.016	0.023	0.017	0.020
Eg(v4)	546.234	546.464	546.957	548.499	546.001	546.831
±	1.014	2.626	2.425	2.901	1.848	2.163
B1g(v4)	640.994	640.999	640.999	640.999	640.995	640.997
±	0.839	1.042	0.885	0.765	0.793	0.865
Eg(v3)	925.178	925.001	925.001	925.321	925.002	925.101
±	0.918	1.041	0.856	1.160	1.393	1.074
A1g(v1)	975.622	975.580	975.253	975.570	975.252	975.455
±	0.022	0.025	0.016	0.023	0.017	0.020
B1g(v3)	1009.924	1009.792	1009.382	1009.825	1009.314	1009.647
±	0.008	0.009	0.008	0.009	0.009	0.009

Table S15: Mean values for 3:1 derived from Table S13_S14

Sample 3:1/Mean	
Ideal	0.25
Weighed	0.2467
µXRF	0.181
Eg(II)	
B1g(II), Mode 207	
Eg(III)	220.47
±	0.13
B2g(v2)	265.28
±	3.24
Eg(I)	354.64
±	0.03
B1g(I)	393.91
±	0.29
A1g(v2)	440.12
±	0.02
Eg(v4)	546.52
±	1.44
B1g(v4)	640.66
±	11.02
Eg(v3)	925.41
±	0.72
A1g(v1)	975.68
±	0.02
B1g(v3)	1009.91
±	0.01

Table S16: Observed Raman modes from sample 1:1/1 (cf. Figure S13) – 5 measurements 1a-e

Sample 1:1/1	Ideal					0.500
	Weighed					0.502
	µXRF					0.396
	1a	1b	1c	1d	1e	Mean
Eg(II)						
B1g(II), Mode 207						
Eg(III)						
B2g(v2)	264.002	265.800			264.990	264.931
±	2.036	1.096			2.439	1.857
Eg(I)	354.816	354.684	354.382	354.478	354.315	354.535
±	0.065	0.046	0.046	0.048	0.051	0.051
B1g(I)	397.330	396.801	396.685	396.488	396.551	396.771
±	0.159	0.120	0.122	0.122	0.135	0.132
A1g(v2)	443.436	442.962	442.835	442.734	442.758	442.945
±	0.027	0.020	0.020	0.021	0.022	0.022
Eg(v4)	545.000	545.001	545.002	547.498	547.497	546.000
±	4.684	2.457	2.435	3.406	3.666	3.330
B1g(v4)	643.822	644.262	643.559	643.843	643.803	643.858
±	0.847	1.608	1.357	1.014	0.687	1.103
Eg(v3)						
A1g(v1)	979.193	978.829	978.645	978.687	978.685	978.808
±	0.025	0.019	0.019	0.019	0.021	0.020
B1g(v3)	1014.220	1013.757	1013.596	1013.606	1013.648	1013.765
±	0.010	0.007	0.007	0.008	0.008	0.008

Table S17: Observed Raman modes from sample 1:1/2 (cf. Figure S13) – 5 measurements 2a-e

Sample 1:1/2					Ideal	0.500
					Weighed	0.502
					µXRF	0.565
	2a	2b	2c	2d	2e	Mean
Eg(II)						
B1g(II), Mode 207						
Eg(III)						
B2g(v2)	266.000	266.000	266.000	265.545	266.000	265.909
±	2.874	2.082	2.667	2.662	2.657	2.588
Eg(I)	352.413	353.000	352.284	352.175	351.986	352.372
±	0.019	0.020	0.018	0.021	0.020	0.019
B1g(I)	397.618	397.260	397.532	397.326	397.537	397.455
±	0.165	0.166	0.159	0.171	0.163	0.165
A1g(v2)	443.883	443.434	443.819	443.870	443.961	443.793
±	0.018	0.020	0.018	0.020	0.019	0.019
Eg(v4)	547.058	545.846	545.666	545.207	546.614	546.078
±	0.723	0.940	0.797	1.182	0.785	0.885
B1g(v4)	645.470	645.505	644.915	643.988	646.598	645.295
±	1.215	2.938	2.323	0.826	0.821	1.625
Eg(v3)	929.174	928.888	929.260	929.178	929.584	929.217
±	0.245	0.327	0.276	0.316	0.271	0.287
A1g(v1)	979.672	979.290	979.594	979.664	979.747	979.593
±	0.019	0.021	0.018	0.021	0.020	0.020
B1g(v3)	1014.932	1014.505	1014.900	1014.966	1015.070	1014.875
±	0.008	0.008	0.008	0.009	0.008	0.008

Table S18: Mean values for 1:1 derived from Table S16-S17

Sample 1:1/mean	
	0.500
Ideal	0.502
Weighed	0.481
Eg(II)	
B1g(II), Mode 207	
Eg(III)	
B2g(v2)	265.542
±	1.851
Eg(I)	353.453
±	0.035
B1g(I)	397.113
±	0.148
A1g(v2)	443.369
±	0.020
Eg(v4)	546.039
±	2.107
B1g(v4)	644.576
±	1.364
Eg(v3)	929.217
±	0.144
A1g(v1)	979.201
±	0.020
B1g(v3)	1014.320
±	0.008

Table S19: Observed Raman modes from sample 1:3/1 (cf. Figure S13) – 5 measurements 1a-e

Sample 1:3/1					Ideal	0.75
					Weighed	0.7589
					µXRF	0.711
	1a	1b	1c	1d	1e	Mean
Eg(II)						
B1g(II), Mode 207						
Eg(III)						
B2g(v2)						
Eg(I)	352.522	352.191	351.508	351.465	351.619	351.861
±	0.057	0.058	0.053	0.063	0.056	0.057
B1g(I)	399.773	399.386	398.944	399.164	398.539	399.161
±	0.113	0.116	0.095	0.125	0.110	0.112
A1g(v2)	446.180	445.888	445.334	445.512	444.953	445.573
±	0.020	0.020	0.018	0.021	0.019	0.020
Eg(v4)	545.035	545.264	545.264	546.436	545.020	545.404
±	5.749	7.198	7.198	0.281	5.382	5.161
B1g(v4)	646.053	646.517	646.738	646.965	644.378	646.130
±	1.130	1.399	1.876	1.215	1.330	1.390
Eg(v3)	930.440	930.940	929.028	929.018	929.021	929.690
±	0.875	0.779	0.879	1.325	1.364	1.044
A1g(v1)	982.064	981.753	981.187	981.314	980.720	981.408
±	0.018	0.018	0.017	0.020	0.018	0.018
B1g(v3)	1017.731	1017.402	1016.779	1016.973	1016.248	1017.027
±	0.007	0.007	0.007	0.008	0.007	0.007

Table S20: Observed Raman modes from sample 1:3/2 (cf. Figure S13) – 5 measurements 2a-e

Sample 1:3/2					Ideal	0.75
					Weighed	0.7589
					µXRF	0.701
	2a	2b	2c	2d	2e	Mean
Eg(II)						
B1g(II), Mode 207						
Eg(III)						
B2g(v2)						
Eg(I)	351.552	351.624	351.446	351.396	351.372	351.478
±	0.070	0.095	0.048	0.044	0.045	0.060
B1g(I)	398.532	399.057	399.219	398.995	398.552	398.871
±	0.138	0.095	0.098	0.099	0.096	0.105
A1g(v2)	444.992	445.498	445.507	445.396	444.980	445.275
±	0.024	0.018	0.018	0.017	0.017	0.019
Eg(v4)						
B1g(v4)	645.272	645.521	646.960	646.946	644.162	645.772
±	3.397	1.332	2.082	15.887	1.709	4.881
Eg(v3)	930.507	930.720	929.367	929.570	930.105	930.054
±	1.842	0.997	1.058	0.662	0.814	1.075
A1g(v1)	980.659	981.569	981.521	981.402	980.890	981.208
±	0.023	0.016	0.016	0.015	0.015	0.017
B1g(v3)	1016.276	1017.192	1017.219	1017.076	1016.470	1016.847
±	0.009	0.007	0.006	0.006	0.006	0.007

Table S21: Mean values for 1:3 derived from Table S19-S20

Sample 1:3/Mean	
	0.75
Weighed	0.7589
µXRF	0.706
Eg(II)	
B1g(II), Mode 207	
Eg(III)	
B2g(v2)	
Eg(I)	351.669
±	0.059
B1g(I)	399.016
±	0.109
A1g(v2)	445.424
±	0.019
Eg(v4)	545.404
±	5.161
B1g(v4)	645.951
±	3.136
Eg(v3)	929.872
±	1.059
A1g(v1)	981.308
±	0.018
B1g(v3)	1016.937
±	0.007

Table S22: Observed Raman modes of $HfSiO_4$ (cf. Figure S13)

					Sample 0:1/Mean	
Sample 0:1	Ideal	1			Ideal	1
	Weighed	0.994			Weighed	0.994
	µXRF	0.994			µXRF	0.994
	1a	1b	1c			
Eg(II)	213.60462	213.48054	213.30422		Eg(II)	213.4631267
±	0.05412	0.03213	0.0325		±	0.039583333
B1g(II), Mode 207	149,48464	150,99106	149,49668		B1g(II), Mode 207	149,99079
±	1.80505	0.86764	2.11706		±	1.59658
Eg(III)	156.29524	156.27806	156.05481		Eg(III)	156.20937
±	0.06539	0.032	0.0272		±	0.04153
B2g(v2)	268.35608	268.32916	268.2086		B2g(v2)	268.2979467
±	0.34599	0.34204	0.24695		±	0.31166
Eg(I)	350.36977	350.23894	350.01821		Eg(I)	350.2089733
±	0.04668	0.02842	0.02919		±	0.034763333
B1g(I)	401.95468	402.00307	401.80184		B1g(I)	401.9198633
±	0.2113	0.08441	0.08587		±	0.127193333
A1g(v2)	449.09527	448.95875	448.76552		A1g(v2)	448.9398467
±	0.02347	0.01433	0.01491		±	0.01757
Eg(v4)					Eg(v4)	
B1g(v4)					B1g(v4)	
Eg(v3)	935.3883	934.59183	935.00012		Eg(v3)	934.9934167
±	0.74413	0.37076	0.41862		±	0.51117
A1g(v1)	985.04772	984.89911	984.7214		A1g(v1)	984.88941
±	0.01953	0.0122	0.01252		±	0.01475
B1g(v3)	1021.00285	1020.9604	1020.77478		B1g(v3)	1020.912677
±	0.01594	0.00652	0.0067		±	0.00972

Raman – Linear Mode Fit in dependence on x(Hf)

Linear fit results of the observed Raman modes of the zircon-hafnon solid solutions series. Linear µXRF fits based on composition of the solid solutions were also carried out (cf. Figure 8).

Eg(II)

	Value
Intercept	201.82491
Slope	-52.14699
X Intercept	3.87031
R-square	1
Pearson R	-1

B1g(II), Mode 207

	Value
Intercept	214.44403
Slope	-58.58618
X Intercept	3.66032

R-square	1
Pearson R	-1

Eg(III)

	Value	Error
Intercept	224.3887	0.40029
Slope	-11.02039	0.56907
X Intercept	20.36123	1.02536

Chi-square reduced	97.25965
R-square	0.99734
Pearson R	-0.99867

µXRF:

	Value	Error
Intercept	224.32001	0.62128
Slope	-10.95494	0.88446
X Intercept	20.4766	1.61273

Chi-square reduced	236.84523
R-square	0.99352
Pearson R	-0.99676

B2g(v2)

	Value	Error
Intercept	263.39996	0.76862
Slope	4.92435	0.78461
X Intercept	-53.48927	8.67782

Chi-square reduced	0.07363
R-square	0.97524
Pearson R	0.98754

µXRF:

	Value	Error
Intercept	263.74004	0.77399
Slope	4.58174	0.79053
X Intercept	-57.56333	10.09972

Chi-square reduced	0.08597
R-square	0.97109
Pearson R	0.98544

Eg(I)

	Value	Error
Intercept	356.11787	0.09934
Slope	-5.83641	0.19613
X Intercept	61.01657	2.03809

Chi-square reduced	23.32645
R-square	0.99662
Pearson R	-0.99831

µXRF:

	Value	Error
Intercept	355.9351	0.15298
Slope	-5.77604	0.31303
X Intercept	61.62272	3.32131

Chi-square reduced	60.34607
R-square	0.99127
Pearson R	-0.99562

B1g(I)

	Value	Error
Intercept	392.36672	0.29569
Slope	9.23768	0.46086
X Intercept	-42.4746	2.14474

Chi-square reduced	9.32703
R-square	0.99259
Pearson R	0.99629

µXRF:

	Value	Error
Intercept	392.47499	0.10676
Slope	9.44253	0.17239
X Intercept	-41.56462	0.76782

Chi-square reduced	1.25708
R-square	0.999
Pearson R	0.9995

A1g(v2)

	Value	Error
Intercept	438.02911	0.45789
Slope	10.50717	0.74165
X Intercept	-41.68857	2.97797

Chi-square reduced	1057.87745
R-square	0.98527
Pearson R	0.99261

µXRF:

	Value	Error
Intercept	438.35563	0.17709
Slope	10.46519	0.29697
X Intercept	-41.88704	1.20203

Chi-square reduced	173.1195
R-square	0.99759
Pearson R	0.99879

Eg(v4)

	Value	Error
Intercept	548.14009	0.40431
Slope	-4.71124	1.3504
X Intercept	116.34739	33.28597

Chi-square reduced	0.09046
R-square	0.85887
Pearson R	-0.92675

µXRF:

	Value	Error
Intercept	547.98795	0.50353
Slope	-4.74275	1.89381
X Intercept	115.54236	46.06232

Chi-square reduced	0.15499
R-square	0.75821
Pearson R	-0.87075

B1g(v4)

	Value	Error
Intercept	640.39481	0.09819
Slope	8.00897	0.45425
X Intercept	-79.95968	4.5403

Chi-square reduced	0.03313
R-square	0.99361
Pearson R	0.9968

µXRF:

	Value	Error
Intercept	640.38962	0.08363
Slope	8.567	0.41306
X Intercept	-74.75077	3.60824

Chi-square reduced	0.02399
R-square	0.99537
Pearson R	0.99768

Eg(v3)

	Value	Error
Intercept	923.27763	0.92207
Slope	11.70482	1.6793
X Intercept	-78.88009	11.39321

Chi-square reduced	3.00031
R-square	0.96046
Pearson R	0.98003

µXRF:

	Value	Error
Intercept	922.34193	0.79214
Slope	12.25708	1.32296
X Intercept	-75.24975	8.18503

Chi-square reduced	1.72773
R-square	0.97723
Pearson R	0.98855

A1g(v1)

	Value	Error
Intercept	973.59439	0.48715
Slope	10.99231	0.7035
X Intercept	-88.57047	5.70592

Chi-square reduced	1010.17044
R-square	0.98786
Pearson R	0.99391

µXRF:

	Value	Error
Intercept	973.87963	0.19013
Slope	10.95489	0.28209
X Intercept	-88.89908	2.30361

Chi-square reduced	165.21084
R-square	0.99801
Pearson R	0.99901

B1g(v3)

	Value	Error
Intercept	1007.36456	0.3505
Slope	13.15983	0.61801
X Intercept	-76.54847	3.61536

Chi-square reduced	4072.15444
R-square	0.99343
Pearson R	0.99671

µXRF:

	Value	Error
Intercept	1007.56198	0.16553
Slope	13.49536	0.30513
X Intercept	-74.65989	1.69737

Chi-square reduced	948.72036
R-square	0.99847
Pearson R	0.99923

IR

Figure S14: Comparison of blank KBr matrix (black) and KBr matrix with zircon (red: crushed single crystal)

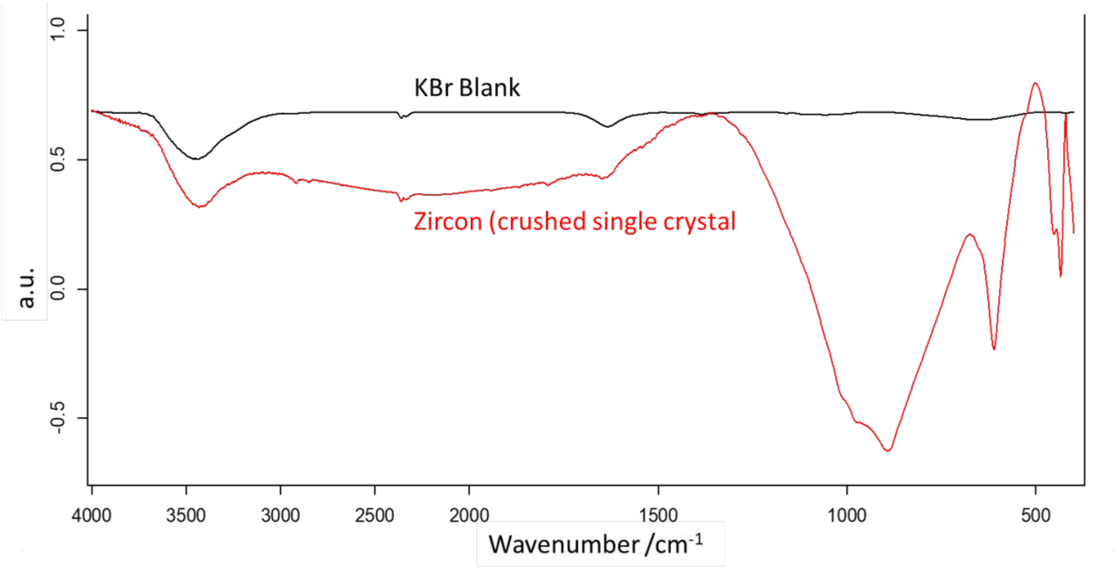

Raman/IR

Figure S15: Wavenumber dependencies of IR and Raman modes on Si-O and ZrHf-O bond length and x(Hf)

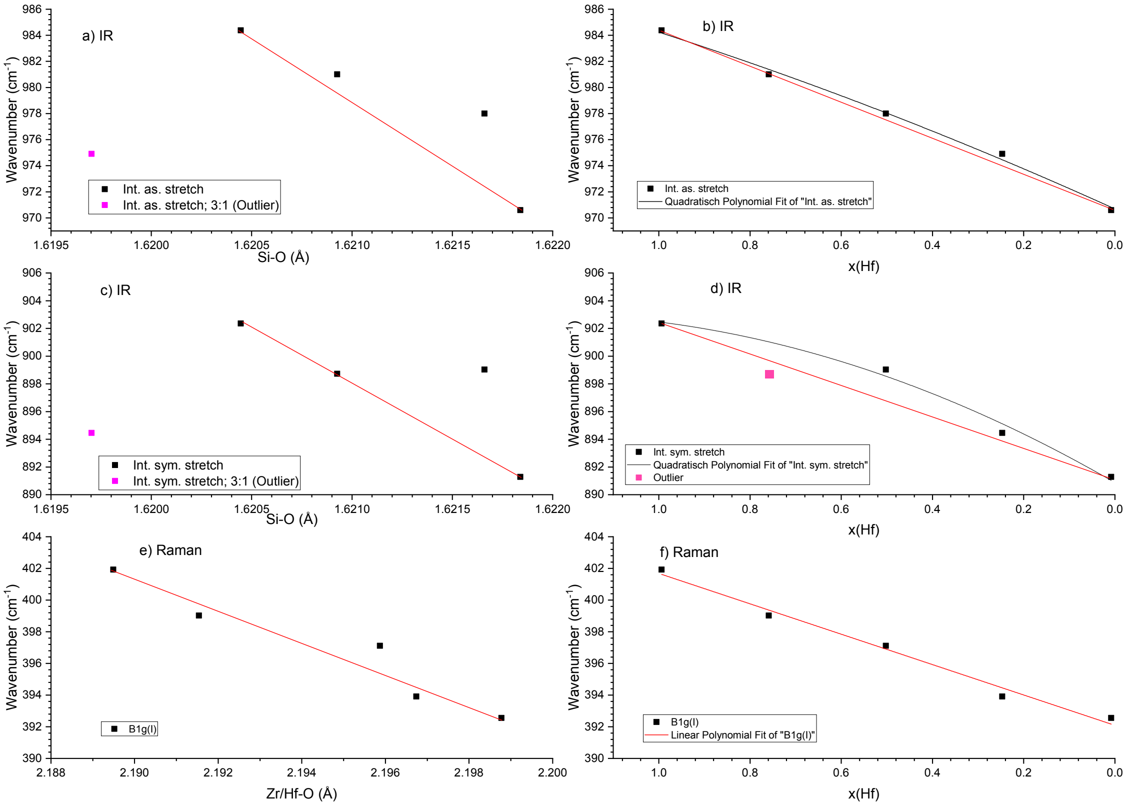

a) IR: Si-O bond length vs. int. as. stretch mode , b) IR: x(Hf) vs. int. as. stretch mode, a) IR: Si-O bond length vs. int. sym. stretch mode , d) IR: x(Hf) vs. int. sym. stretch mode e) Raman: Zr/Hf-O bond length vs. B1g (I), f) Raman: x(Hf) vs. B1g (I) vs.; Magenta: Outlier; Red line: Guide to the eye from a)-e) and linear fit in f)

Table S23-S27: IR-Modes of H_2O and organic residues

Table S23: Stretching modes of H_2O of flux grown grown samples of the solid solution series

Sample	Ideal x(Hf)	Measured x(Hf)	Wavenumber cm^{-1}
1:0	0.000	0.008	3434.17
3:1	0.250	0.247	3434.00
1:1	0.500	0.502	3435.20
1:3	0.750	0.759	3434.74
0:1	1.000	0.994	3434.89

Table S24: Stretching modes of H_2O of hydrothermal samples of the solid solution series

Sample	Ideal x(Hf)	Measured x(Hf)	Wavenumber cm^{-1}
1:0	0.000	0.008	3430.22
7:1	0.125	0.146	3433.65
3:1	0.250	0.293	3433.01
5:3	0.375	0.421	3434.10
1:1	0.500	0.550	3433.90
3:5	0.625	0.670	3434.25
1:3	0.750	0.778	3434.33
1:7	0.875	0.892	3434.85
0:1	1.000	0.994	3434.63

Table S25: Stretching modes of H_2O of flux grown grown samples of the solid solution series

Sample	Ideal x(Hf)	Measured x(Hf)	Wavenumber cm^{-1}
1:0	0.000	0.008	1648.30
3:1	0.250	0.247	1647.90
1:1	0.500	0.502	1647.50
1:3	0.750	0.759	1645.65
0:1	1.000	0.994	1647.30

Table S26: Stretching modes of H_2O of hydrothermal samples of the solid solution series

Sample	Ideal x(Hf)	Measured x(Hf)	Wavenumber cm^{-1}
1:0	0.000	0.008	1636.98
7:1	0.125	0.146	1635.54
3:1	0.250	0.293	1637.28
5:3	0.375	0.421	1636.48
1:1	0.500	0.550	1637.26
3:5	0.625	0.670	1636.48
1:3	0.750	0.778	1637.08
1:7	0.875	0.892	1636.62
0:1	1.000	0.994	1637.18

Table S27: Modes of organic compounds at approx. 1395 cm^{-1} and 1335 cm^{-1} in the hydrothermal samples of the solid solution series

Sample	Ideal x(Hf)	Measured x(Hf)	Wavenumber cm^{-1}	
1:0	0.000	0.008	1396.54	1331.90
7:1	0.125	0.146	1396.10	1333.10
3:1	0.250	0.293	1393.00	1332.40
5:3	0.375	0.421	1397.35	1333.86
1:1	0.500	0.550	1395.11	1332.94
3:5	0.625	0.670	1396.35	1335.57
1:3	0.750	0.778	1396.12	1333.67
1:7	0.875	0.892	1396.32	1336.40
0:1	1.000	0.994	1396.97	1334.39

References

[1] Zagorac, D.; Müller, H.; Ruehl, S.; Zagorac, J.; Rehme, S. Recent developments in the Inorganic Crystal Structure Database: theoretical crystal structure data and related features. *Journal of Applied Crystallography* **2019**, *52 (5)*, 918–925.

[2] Finger, L. W. “Refinement of the crystal structure of zircon. *Carnegie Inst. Wash. Yearbook*, *73*, **1974**, 544–547.

[3] Robinson, K.; Gibbs, G. V.; Ribbe, P. H. The structure of zircon: a comparison with garnet. *American Mineralogist: Journal of Earth and Planetary Materials* **1971**, *56*, 782–790.

[4] Siggel, A.; Jansen, M. Röntgenographische untersuchungen zur bestimmung der einbauposition von seltenen erden (Pr, Tb) und vanadium in zirkonpigmenten. *Zeitschrift für anorganische und allgemeine Chemie* **1990**, *583*, 67–77.

[5] Mursic, Z.; Vogt, T.; Boysen, H.; Frey, F. Single-crystal neutron diffraction study of metamict zircon up to 2000 K. *Journal of Applied Crystallography* **1992**, *25*, 519–523.

[6] Finch, R. J.; Hanchar, J. M.; Hoskin, P. W. O.; Burns, P. C. Rare-earth elements in synthetic zircon: Part 2. A single-crystal X-ray study of xenotime substitution. *American Mineralogist* **2001**, *86*, 681–689.

[7] Yu, S. C.; Tung, S. F.; Lee, J. S.; Bai, W. J.; Yang, J. S.; Fang, Q. S.; Zhang, Z. M. Kuo, C. T. Structural and spectroscopic features of mantle-derived zircon crystals from Tibet. *Western Pacific Earth Sciences* **2001**, *1*, 47–58.

[8] Hazen, R. M.; Finger, L. W. Crystal structure and compressibility of zircon at high pressure. *American Mineralogist* **1979**, *64*, 196–201.

[9] Kolesov, B. A.; Geiger, C. A.; Armbruster, T. The dynamic properties of zircon studied by single-crystal X-ray diffraction and Raman spectroscopy. *European Journal of Mineralogy* **2001**, *13*, 939–948.

[10] Hassel, O. XIV. Die Kristallstruktur einiger Verbindungen von der Zusammensetzung MRO4. I. Zirkon ZrSiO4. *Zeitschrift für Kristallographie-Crystalline Materials* **1926**, *63*, 247–254.

[11] Wyckoff, R. W. G.; Hendricks, S. B. IV. Die Kristallstruktur von Zirkon und die Kriterien für spezielle Lagen in tetragonalen Raumgruppen. *Zeitschrift für Kristallographie-Crystalline Materials* **1928**, *66*, 73–102.

[12] Binks, W. The crystalline structure of zircon. *Mineralogical magazine and journal of the Mineralogical Society* **1926**, *21*, 176–187.

[13] Krstanović, I. R. Redetermination of the oxygen parameters in zircon (ZrSiO4). *Acta Crystallographica* **1958**, *11*, 896–897.

[14] Torres, F. J.; Tena, M. A.; Alarcón, J. Rietveld refinement study of vanadium distribution in V+4–ZrSiO4 solid solutions obtained from gels. *Journal of the European Ceramic Society* **2002**, *22*, 1991–1994.

[15] Chaplot, S. L.;Mittal, R.; Busetto, E.; Lausi, A. Thermal expansion in zircon and almandine: Synchrotron x-ray diffraction and lattice dynamical study. *Physical Review B* **2002**, *66*, 064302.

[16] Kittiauchawal, T.; Mungchamnankit, A.; Sujinnapram, S.; Kaewkhao, J; Limsuwan, P. The Effect of Heat Treatment on Crystal Structure in Zircon Monitored by ESR and XRD. *Procedia Engineering* **2012**, 32, 706–713.

[17] Valéro, R.; Paillaud, J. L.; Durand, B.; Guth, J. L. ; Chopin, T. Rietveld refinement of two fluoro-hydroxy-zircons. *European Journal of Solid State and Inorganic Chemistry* **1998**, *10*, 735–743.

[18] Grüneberger, A. M.; Schmidt, C.; Jahn, S.; Rhede, D.; Loges, A; Wilke, M. Interpretation of Raman spectra of the zircon–hafnon solid solution. *European Journal of Mineralogy* **2016**, *28*, 721–733.

[19] Speer, J. A. and Cooper, B. J. Crystal structure of synthetic hafnon, HfSiO4, comparison with zircon and the actinide orthosilicates. *American Mineralogist* **1982**, *67*, 804–808.

[20] Fuhrmann, J. and Pickardt, J. Bildung von HfSiO4-Einkristallen durch chemische Transportreaktion. *Zeitschrift für anorganische und allgemeine Chemie* **1986**, *532*, 171–174.

[21] Cota, A.; Burton, B. P.; Cháın, P.; Pavón, E.; Alba, M. D. Solution properties of the system ZrSiO4-HfSiO4: a computational and experimental study. *The Journal of Physical Chemistry C* **2013**, *117*, 10013–10019.

[22] Ramakrishnan, S. S.; Gokhale, K. V. G. K.; Subbarao, E. C. Solid solubility in the system zircon-hafnon. *Materials Research Bulletin* **1969**, 4, 323–327.

[23] Estevenon, P.; Kaczmarek, T.; Rafiuddin, M. R.; Welcomme, É.; Szenknect, S.; Mesbah, A.; Moisy, P.; Poinssot, C.; Dacheux, N. Soft Hydrothermal Synthesis of Hafnon, HfSiO4. *Crystal Growth & Design* **2020**, *20*, 1820–1828.
